# Supplementary material for: Beyond the catalytic serine: selective protease engagement with covalent macrocyclic activity-based probes
Source: Chem Sci. 2026 Oct 9. Online ahead of print. doi: 10.1039/d6sc04245g (PMC13659818; doi:10.1039/d6sc04245g)
Supplement: Supplementary file 1 [file SC-OLF-D6SC04245G-s002.pdf]

Supplementary Information (SI) for Chemical Science.
This journal is © The Royal Society of Chemistry

Supporting Information For:

Beyond the Catalytic Serine: Selective Protease Engagement with Covalent Macrocyclic Activity-based Probes

Marina Barrueco[1], Ellie Hyde[1], Ella Sawtell[1], Jack V. Mills[1], Vaia Nikoloudi[1], Marianne Enget[1], Maisem Laabei[2], Markus Lakemeyer[3] & Scott Lovell[1,*]

[1]Department of Life Sciences, University of Bath, Bath, United Kingdom

[2]School of Biochemistry and Biomedical Sciences, University of Bristol, Bristol, United Kingdom

[3] Cluster of Excellence Balance of the Microverse and Institute for Organic Chemistry and Macromolecular Chemistry (IOMC), Friedrich Schiller University, 07743 Jena, Germany

Table of Contents

1 Materials and Methods *5*

1.1 Chemicals and Reagents 5

1.2 Equipment List 5

2 Biological and Biochemical Methods *6*

2.1 Biotinylation of Human Plasma Kallikrein 6

2.2 Phage Production 6

2.3 On-Phage Peptide Cyclisation 8

2.4 Phage Panning 8

2.5 Sample Preparation for Next-Generation Sequencing 11

2.6 Next-Generation Sequencing Analysis 15

2.7 Enzyme Assays 16

2.8 In-gel Fluorescence 18

2.9 Intact Protein Mass Spectrometry 18

2.10 Expression of Plasma Kallikrein Mutants 19

2.11 Tryptic Digest LC–MS/MS Analysis 21

2.12 PC3 Cell Culture 23

2.13 Plasma Kallikrein Detection in Human Plasma 24
2.14 cmABP Stability Tests 24
3 Chemical Synthesis 26
3.1 Chemical Reagents 26
3.2 General Procedures 27
3.2.1 Automated Peptide Synthesis: 27
3.2.2 Flash Chromatography: 27
3.2.3 Analytical LC-MS: 28
3.2.4 Mass-Spectrometry: 28
3.2.5 Sample Drying: 28
3.3 Chemical Procedures 28
3.3.1 Installation of Fluorosulfate Using Fluorosulfate Tyrosine. 28
3.3.2 Synthesis of Fmoc-Tyrosine Fluorosulfate 29
3.3.3 DBMB/TBMB Peptide Cyclisation 31
3.3.4 Installation of Fluorosulfate Using AISF 32
3.3.5 Installation of Sulfonyl Fluoride Using 5-(Fluorosulfonyl)-2-methoxybenzoic acid 32
3.3.6 Installation of Sulfonyl Fluoride Using 3/4/5-Benzylsulfonyl Fluoride 33
3.3.7 Synthesis of Diphenylphosphonate 29 34
4 Supplementary Figures 39
4.1 Volcano Plot of NGS Data 39
4.2 Labelling of Plasma Kallikrein Mutants with FP-TAMRA 40
4.3 Inhibition Constant Plots for 10 and 12 40
4.4 Enzyme Activity Assay of 23 with WT and K192R PKa 41
4.5 Tryptic-Digest LC-MS/MS 42
4.6 Intact-protein mass spectrometry analysis of covalent modification kinetics for cyclic and linear probe. 43
4.7 In-gel Fluorescence Profiling Against Serine Protease Panel 44
4.8 PC3 Cell Media Labelling with FP TAMRA 46
5 Probe Analytical Data 46
6 References 81

Supplementary Figures

Figure S1. Volcano Plot of NGS Data against Plasma Kallikrein................................38

Figure S2. In-gel fluorescence mutant labelling of FP-TAMRA................................38

Figure S3. Enzymatic Inhibition Study of 10 and 12................................39

Figure S4. Enzyme Activity Assay of 23 with WT and K192R PKa................................40

Figure S5. MS1 analysis of apo and 23-conjugated plasma kallikrein peptides................................41

Figure S6. EThcD MS2 localisation of 23 modification to Lys192................................42

Figure S7. Intact-protein mass spectrometry analysis of cyclic and linear probe................................42

Figure S8. In-gel fluorescence profile labelling of 21................................43

Figure S9. In-gel fluorescence profile labelling of FP-TAMRA................................44

Figure S10. Labelling of PC3 Cell Media with FP-TAMRA................................45

Supplementary Tables

Table S1. Washing steps in the phage panning experiment......................................10

Table S2. List of primers used in NGS sample preparation.......................................11

Table S3. Reagents used in primary PCR for NGS sample preparation.....................12

Table S4. Conditions used in primary PCR for NGS sample preparation...................13

Table S5. Reagents used in secondary PCR for NGS sample preparation.................13

Table S6. Conditions used in secondary PCR for NGS sample preparation..............14

Table S7. Specific barcoded-primers used for each NGS sample analysed.............14

Table S8. Unnatural amino acids for synthesis of cmABPs.......................................24

Table S9. Timetable for probe stability analysis by LC-MS.......................................25

Supplementary Schemes

Scheme S1. Solid-phase synthesis of compounds **9-16**..29

Scheme S2. Synthesis of Fmoc-fluorosulfate tyrosine (**5**).......................................28

Scheme S3. DBMB cyclisation..31

Scheme S4. Solid-phase synthesis of compounds **17-22**..32

Scheme S5. Solid-phase synthesis of compounds **23-24**..33

Scheme S6. Solid-phase synthesis of compounds **25-27**..34

Scheme S7. Synthesis of Diphenylphosphonate warhead..33

Scheme S8. Synthesis of **29**..36

1 Materials and Methods

1.1 Chemicals and Reagents

All chemicals and reagents were purchased from commercial suppliers and used without further purification. Rink Amide resin (100-200 mesh) and 2-Chlorotrityl chloride resin (100-200 mesh, 1% DVB) were obtained from Merck. Natural Fmoc-amino acids were obtained from Fluorochem. Amicon 3 kDa and 10 kDa centrifugal filters were obtained from Merck. Zeba spin columns (7 kDa) were obtained from Thermo Fisher Scientific. Purified plasma kallikrein was obtained from Innovative Research. Human urokinase-type plasminogen activator was obtained from AcroBiosystems. Streptavidin- and neutravidin-coated magnetic beads were purchased from Thermo Fisher Scientific. 2YT and agar media were purchased from Merck. All remaining biological products and chemical reagents were obtained from Fluorochem, VWR, Sigma Aldrich, Thermo Fisher Scientific or Merck.

1.2 Equipment List

DNA amplification was performed using a SimpliAmp Thermal Cycler (Fisher). Gel electrophoresis was performed using an mPAGE Mini Gel Tank (Millipore) and PowerPac™ Basic (BioRad). Fluorescent plate-based enzymatic assays were carried out using a VANTAstar Plate reader (BMG Labtech) in kinetic mode for a minimum of 5 min. In-gel fluorescence-based assays were imaged on a BioRad ChemiDoc™ Imaging System. Peptide synthesis was performed on a Multipep 2 (CEM). Flash Chromatography was performed on either a CombiFlash NextGen 300 (Teledyne ISCO) or ECS28P01 Compact Preparative System (ECOM). Sample drying was performed using a Genevac EZ-2 or Edwards Modulyo Freeze Dryer. High resolution mass spectrometry data was collected using an Agilent 1260 Infinity II coupled to a Triple Quadrupole Agilent 6545Q+TOF ESI.

2 Biological and Biochemical Methods

2.1 Biotinylation of Human Plasma Kallikrein

Human plasma kallikrein was biotinylated using EZ-Link-Sulfo-NHS-LC-Biotin (Thermo Scientific). Briefly, 0.2 mg of plasma kallikrein (10 µM in PBS, pH 7.4) was incubated with a 10-fold molar excess of freshly prepared EZ-Link-Sulfo-NHS-LC-Biotin for 60 min at RT. Unreacted biotinylation reagent was subsequently removed via size-exclusion chromatography using a disposable Zeba spin desalting column. The final concentration of the biotinylated target was determined using a BCA protein assay.

To confirm successful biotinylation, the target protein was subjected to a magnetic bead capture assay. Biotinylated plasma kallikrein (1 µg) and non-biotinylated plasma kallikrein (1 µg) were incubated with 25 µL of streptavidin-coated magnetic beads for 60 min at RT. The bead fractions (post-boiling) and the unbound supernatant fractions for both samples were then analysed by SDS-PAGE followed by Coomassie Brilliant Blue staining. Efficient biotinylation was visually verified by the presence of the biotinylated protein exclusively in the boiled bead fraction, whereas the non-biotinylated control remained entirely in the supernatant.

2.2 Phage Production

An $AXCX_7C$ peptide phage library was utilised for peptide selection against plasma kallikrein.[1] The phage particles feature a disulfide-free pIII coat protein, to prevent reaction of linker with cysteines on the pIII protein, which significantly lowers the phage infectivity. An aliquot (1 mL) of the phage peptide library in TG1 *E.coli* bacteria was thawed on ice and used to inoculate 0.5L of 2YT rich medium supplemented with 30 µg ml^{-1} chloramphenicol. The culture was incubated for 16 h at 30 °C with vigorous shaking (250 rpm). Bacteria were pelleted by centrifugation at 8,500 rpm for 30 min at 4 °C and the supernatant was collected.

The supernatant was combined with a 25% (v/v) precipitation buffer (20% w/v PEG 8000, 2.5 M NaCl) and incubated for 60 min at 4 °C with gentle swirling every 15 min to maximise phage recovery. Precipitated phage particles were then pelleted by centrifugation at 9,000 rpm for 60 min at 4 °C. The supernatant was discarded and the inner walls of the tube were carefully dried to maximise PEG removal without disturbing the phage pellet. The pellet was resuspended in 10 mL of sterile PBS and centrifuged at 16,000 rpm for 10 min at 4 °C to remove residual cell debris. The cleared supernatant was transferred to a fresh tube containing 25% (v/v) of the PEG precipitation solution and incubated at 4 °C for an additional 60 min. Purified phage particles were collected by precipitation at 9,000 rpm for 60 min at 4 °C. The supernatant was discarded, and the final pellet was resuspended in a 50% (v/v) glycerol in PBS solution to a final 1 mL volume. A final purification step was performed by centrifugation at 18,000 rpm for 20 min at 4 °C in a bench-top centrifuge to remove cell debris. The purified phage stock was stored at -20 °C until further use.

To monitor phage production, serial 10-fold dilutions of the stock were prepared in PBS. Separately, a 5 mL 2YT culture from a single colony of *E.coli* TG1 cells was grown overnight and used to prepare a fresh culture in exponential growth phase (OD_{600} = 0.4). To allow for phage infection, 20 μL of the highly diluted phage samples (e.g., 10^6, 10^7, 10^8) were mixed with 180 μL of the exponential phase TG1 cells. The infection mixtures were incubated for 90 min at 37 °C. Subsequently, 10 μL of each mixture was spotted in triplicate onto 2YT agar plates supplemented with 30 μg ml^{-1} chloramphenicol and incubated overnight at 37 °C. The following day, visible colonies were counted, and the number of phage particles per mL of solution was determined using the following formula:

$$\frac{\text{phage}}{\text{mL}} = \frac{x}{10} \times 10^{y} \times 10^{3}$$

where x is the average number of colonies across three replicates within a specific titer and y is the absolute dilution factor of the titer yielding countable colonies.

2.3 On-Phage Peptide Cyclisation

Phage-displayed peptides were cyclised using 1,3-Bis(bromomethyl)benzene. First, peptide cysteines were reduced by incubating the phage library with 1 mM TCEP in 50% (v/v) glycerol-PBS for 30 min at RT. The reduced phage particles were then precipitated from the media using the previously described PEG/NaCl buffer; due to the smaller working volume, incubation and centrifugation times were reduced to 30 min. Following supernatant removal, the phage pellet was resuspended in alkylation buffer (50 mM NH_4HCO_3, 5 mM EDTA, pH 8.5) to facilitate cysteine alkylation. A pre-made solution of DBMB in MeCN was subsequently added to achieve a final linker concentration of 40 μM and 20% (v/v) MeCN in a total reaction volume of 1 mL. The reaction was incubated with continuous mixing for 60 min at 30 °C.

Following alkylation, a final phage precipitation was performed using PEG/NaCl as described above. The cyclised phage pellet was then resuspended in 50% (v/v) glycerol in PBS for storage at -20 °C, or, if proceeding directly to biopanning experiments, in plasma kallikrein activity buffer (10 mM Tris-Cl, 150 mM NaCl, 10 mM $MgCl_2$, 1 mM $CaCl_2$, pH 7.4) supplemented with 0.3% (v/v) Tween-20 and 3% (w/v) BSA.

2.4 Phage Panning

To enrich for high-affinity peptide binders, three rounds of biopanning were performed against immobilised target protein. Biotinylated plasma kallikrein was immobilised onto either streptavidin- or neutravidin-coated magnetic beads (Cytiva). To prevent the enrichment of matrix-specific background binders, the capture matrix was alternated between rounds (e.g., neutravidin beads were utilised in the third round). To increase selection stringency for successive panning rounds, the quantity of immobilised protein was reduced from 5 μg for the first round to 2 μg for rounds two and three. Each panning round included a parallel negative control, wherein half of the input phage was panned against target-free magnetic beads to account for non-specific binding.

The day prior to biopanning, a 5 mL 2YT culture was inoculated from a single colony of *E.coli* TG1. If the phage-displayed cyclic peptide library stock was stored in 50% (v/v) glycerol, it was first precipitated using the PEG/NaCl protocol and resuspended in panning buffer (plasma kallikrein activity buffer supplemented with 0.3% (v/v) Tween-20 and 3% (w/v) BSA).

Prior to target incubation, the cyclised phage library was subjected to a pre-clearing step to deplete non-specific bead binders. A 30 μL aliquot of a 1:1 mixture of streptavidin and neutravidin magnetic beads was washed three times with 200 μL with plasma kallikrein activity buffer. The phage library was added to these washed beads and incubated for 60 min at RT with continuous mixing.

Following the phage pre-clearing step, the beads were magnetically separated and discarded, while the supernatant containing the pre-cleared phage was retained. This phage library was equally divided: one half was combined with biotinylated plasma kallikrein (2-5 μg) in panning buffer to form the positive selection mixture, while the remaining half was combined with an equal volume of panning buffer without target to serve as the negative control. Both samples were then incubated at 37 °C with slow rotation for 30 min.

Simultaneously, two 25 μL aliquots of the selected magnetic beads (streptavidin or neutravidin) were washed three times with 200 μL of activity buffer, followed by resuspension in 50 μL of panning buffer and blocking for 30 min at RT with rotation. Following the initial in-solution incubation, the pre-blocked magnetic beads were added to each library mixture and incubated for 30 min at RT with slow rotation to capture the biotinylated target complexes.

After the target capture step, the beads were isolated using a magnetic rack and washed ten times with PBS-T (PBS containing 0.1% (v/v) Tween-20). To increase selection stringency, the duration of the individual wash steps was progressively extended across successive panning rounds (See Table 1).

Supplementary Table 1: Washing steps in the phage panning experiment

Panning Round	PBS-T
1st round	10 x 2 min
2nd round	4 x 2 min, 2 x 10 min, 4 x 2 min
3rd round	3 x 2 min, 4 x 20 min, 3 x 2 min

To minimise the retention of non-specific, plastic-binding phage, the samples were transferred to fresh microcentrifuge tubes after every two washes. After the final wash, the buffer was removed, the beads resuspended in 100 μL elution buffer (50 mM Glycine, pH 2.2) and rotated at RT for 10 min. The beads were then captured and the eluted phage supernatants transferred to new tubes containing 50 μL of neutralisation buffer (1 M Tris-Cl, pH 8.0). A 10 μL aliquot from both the target and negative control panning outputs was reserved for phage titration. The remaining eluted phage from both conditions were used to inoculate two separate 50 mL cultures of *E.coli* TG1 cells in the exponential growth phase (OD_{600} = 0.4) in 2YT media. Following a 90 min infection period at 37 °C, the cells were pelleted by centrifugation at 4,000 rpm for 10 min at 4 °C. The cell pellets were resuspended in 1 mL of 2YT media and plated onto two large 2YT agar plates supplemented with chloramphenicol and incubated overnight at 37 °C. The following day, the amplified cells were harvested by scraping the plates with 2 mL of 2YT media per plate. Glycerol was added to a final concentration of 50% (v/v), and 1 mL aliquots were stored at -80 °C. To initiate the next round of selection, input phage particles were produced from these glycerol stocks following the procedure described in **Section 2.2**. The enrichment factor for each panning round was determined by calculating the ratio of the phage titre recovered from the target (plasma kallikrein) output against the titre from the negative control output.

2.5 Sample Preparation for Next-Generation Sequencing

Following three rounds of biopanning, DNA from both the phage enriched against plasma kallikrein and the negative control was prepared for Next-Generation Sequencing (NGS) analysis. All samples were processed in triplicate. A two-step PCR amplification strategy was employed: the primary PCR utilised a conserved set of five forward and five reverse primers to amplify the peptide-encoding region, while the secondary PCR step utilised unique combinations of forward and reverse primers to append sample-specific indices (barcodes) for multiplexed sequencing analysis (See **Supplementary Table 2**).

Supplementary Table 2: List of primers used in NGS sample preparation

Round	Fwd/Rvr	Primer	Sequence
I	Fwd.	1a	TCGTCGGCAGCGTCAGATGTGTATAAGAGACAGCCAGAGCCACCCTCGCTACCG
		1b	TCGTCGGCAGCGTCAGATGTGTATAAGAGACAG**N**CCAGAGCCACCCTCGCTACCG
		1c	TCGTCGGCAGCGTCAGATGTGTATAAGAGACAG**NN**CCAGAGCCACCCTCGCTACCG
		1d	TCGTCGGCAGCGTCAGATGTGTATAAGAGACAG**NNN**CCAGAGCCACCCTCGCTACCG
		1e	TCGTCGGCAGCGTCAGATGTGTATAAGAGACAG**NNNN**CCAGAGCCACCCTCGCTACCG
	Rvrs.	2a	GTCTCGTGGGCTCGGAGATGTGTATAAGAGACAGGCTATGCGGCCCAGCCGGCC
		2b	GTCTCGTGGGCTCGGAGATGTGTATAAGAGACAGG**N**CTATGCGGCCCAGCCGGCC
		2c	GTCTCGTGGGCTCGGAGATGTGTATAAGAGACAGG**NN**CTATGCGGCCCAGCCGGCC
		2d	GTCTCGTGGGCTCGGAGATGTGTATAAGAGACAGG**NNN**CTATGCGGCCCAGCCGGCC
		2e	GTCTCGTGGGCTCGGAGATGTGTATAAGAGACAGG**NNNN**CTATGCGGCCCAGCCGGCC
	F	S505	AATGATACGGCGACCACCGAGATCTACAC**GTAAGGA**GTCGTCGGCAGCGTC

2		S506	AATGATACGGCGACCACCGAGATCTACAC**ACTGCATA**TCGTCGGCAGCGTC
		S507	AATGATACGGCGACCACCGAGATCTACAC**AAGGAGTA**TCGTCGGCAGCGTC
	**Rvrs.**	N704	CAAGCAGAAGACGGCATACGAGAT**GCTCAGGA**GTCTCGTGGGCTCGG
		N705	CAAGCAGAAGACGGCATACGAGAT**AGGAGTCC**GTCTCGTGGGCTCGG

To isolate template DNA for the primary PCR, three 200 μL aliquots were sampled from the third-round plasma kallikrein panning output glycerol stock and the corresponding third-round negative control glycerol stock. Plasmid DNA was purified from these aliquots using the Monarch Plasmid Miniprep Kit (New England Biolabs) according to the manufacturer's instructions. For each subsequent primary PCR amplification, 200 ng of the purified plasmid DNA was utilised as the template (See **Supplementary Table 3** and **4**).

Supplementary Table 3: Reagents used in primary PCR for NGS sample preparation.

Reagent	Volume
1a	1 µL
1b	1 µL
1c	1 µL
1d	1 µL
1e	1 µL
2a	1 µL
2b	1 µL
2c	1 µL
2d	1 µL
2e	1 µL
dNTP mix	1 µL

Plasmid DNA	200 ng
5X HF buffer	10 μL
Phusion Enzyme	2 μL
dH_2O	Made up to 50 μL

Supplementary Table 4: Conditions used in primary PCR for NGS sample preparation.

Temperature (°C)	Time	Cycles
95	5 min	1
95	30 sec	
72	45 sec	25
72	1 min	
72	5 min	1
4	∞	

The unpurified amplicon from the primary PCR was utilised directly as the template for the secondary indexing PCR, which was assembled as follows (See Table 5, 6, 7).

Supplementary Table 5: Reagents used in secondary PCR for NGS sample preparation.

Reagent	Volume
Fwd.	1 μL
Rvrs.	1 μL
dNTP mix	1 μL

PCR 1 DNA	2 μL
5X HF buffer	10 μL
Phusion Enzyme	2 μL
dH_2O	Made up to 50 μL

Supplementary Table 6: Conditions used in secondary PCR for NGS sample preparation.

Temperature (°C)	Time	Cycles
95	5 min	1
95	30 sec	
61	45 sec	25
72	1 min	
72	5 min	1
4	∞	

Supplementary Table 7: Specific barcoded-primers used for each NGS sample analysed.

Fwd.	Rvrs.	Sample
S505	N704	Control-1
S506	N704	Control -2
S507	N704	Control -3
S505	N705	Protein-1

S506	N705	Protein -2
S507	N705	Protein-3

The secondary PCR products were resolved by electrophoresis on a 2% (w/v) agarose gel in Tris-Borate-EDTA (TBE) buffer at 100 V for 30 min, utilising a Low Molecular Weight DNA Ladder (New England Biolabs) as a reference standard. Amplicons corresponding to the expected size of 270 bp were excised from the gel and purified using the Monarch DNA Gel Extraction Kit (New England Biolabs) according to the manufacturer's instructions. Finally, equimolar amounts of the purified, barcoded amplicons were then pooled together to generate the final library for Next Generation Sequencing (NGS) analysis.

2.6 Next-Generation Sequencing Analysis

Raw sequencing data were first processed to ensure high read quality. Sequences containing excessive errors, ambiguous bases, or lacking the correct peptide sequence were discarded. The remaining high-quality peptide sequences were arranged as count matrices for downstream statistical analysis.

To identify peptides specifically enriched in the plasma kallikrein samples compared to the control group, differential enrichment analysis was conducted using the DESeq2 package in R. Count data were normalised, and statistical significance was calculated using the default DESeq2 pipeline. Peptides were defined as significantly enriched if they demonstrated a $\log_2$ (Fold Change) > 1.5 and an adjusted p-value (p_{adj}) < 0.05.

The significantly enriched peptides identified via DESeq2 were clustered based on sequence similarity using the Gibbs clustering algorithm (GibbsCluster version 2.0). This grouped the related peptide families and determined consensus sequence motifs.

Custom scripts utilised for sequence filtering, quality control, and data analysis are available from the corresponding author upon request.

2.7 Enzyme Assays

The inhibitory activity of the cyclic peptides was evaluated by incubating the target proteases with varying concentrations of the inhibitors and subsequently quantifying residual enzyme activity using specific fluorogenic substrates. Briefly, the peptide inhibitors were pre-incubated with the respective target protein in assay buffer at 37 °C for 30 min. Following the addition of the substrate, enzyme activity was determined by measuring fluorescence intensity at 1 min intervals over a 30 min period. The inhibitory constant (K_i) was calculated according to the Cheng–Prusoff equation: $K_i = IC50/(1+([S]_0/K_M))$, where IC50 is the functional strength of the inhibitor, $[S]_0$ is the total substrate concentration, and K_M is the Michaelis–Menten constant.[2] For covalent inhibitors, values obtained using this assay are reported as apparent inhibitory constants ($K_{i,\ app}$), as covalent modification may occur during the 30 min pre-incubation period.

Plasma Kallikrein Assay conditions

- **Buffer:** 50 mM Tris-HCl (pH 7.4), 150 mM NaCl, 10 mM $MgCl_2$, 1 mM $CaCl_2$, 0.01% (w/v) BSA, 0.01% (v/v) Triton X-100, 5% (v/v) DMSO.
- **Enzyme:** Purified Human Plasma Kallikrein (Innovative Research), 10 nM
- **Substrate:** Cbz-Phe-Arg-AMC (Thermo Fisher Scientific), 50 μM

Urokinase-Type Plasminogen Activator (uPA) Assay conditions

- **Buffer:** 50 mM Tris-HCl (pH 7.4), 150 mM NaCl, 10 mM $MgCl_2$, 1 mM $CaCl_2$, 0.01% (w/v) BSA, 0.01% (v/v) Triton X-100, 5% (v/v) DMSO.

- **Enzyme:** Human PLAU/uPA protein (AcroBiosystems), 1.5 nM
- **Substrate:** Cbz-Gly-Gly-Arg-AMC (SantaCruz Biotechnologies), 50 μM.

Trypsin Assay conditions

- **Buffer:** 50 mM ammonium bicarbonate (pH 8.0).
- **Enzyme:** Sequencing Grade Modified Trypsin (Promega), 50 nM.
- **Substrate:** Cbz-Phe-Arg-AMC, 50 μM.

Thrombin Assay conditions

- **Buffer:** 50 mM Tris-HCl (pH 7.5), 10 mM $CaCl_2$, 150 mM NaCl, 0.05% (w/v) Brij-35.
- **Enzyme:** Recombinant Human Coagulation Factor II/Thrombin (R&D Systems), 0.6 nM.
- **Substrate:** BOC-VPR-AMC, 50 μM.

Plasmin Assay conditions

- **Buffer:** 0.1 M Tris-HCl (pH 7.5), 0.1 M NaCl.
- **Enzyme:** Human Plasminogen Protein (R&D Systems), 6 nM.
- **Substrate:** SUC-Ala-Phe-Lys-AMC, 50 μM.

KLK2 Assay conditions

- **Buffer:** 50 mM Tris-HCl (pH 7.5), 150 mM NaCl, 10 mM $CaCl_2$, 0.05% (w/v) Brij-35.
- **Enzyme:** Recombinant Human Kallikrein 2 (AcroBiosystems),12 nM.
- **Substrate:** Morpholin-Val-Dab-4Pal-Arg-ACC, 10 μM.

KLK14 Assay conditions

- **Buffer:** 50 mM Tris-HCl (pH 8.0), 150 mM NaCl, 0.05% (w/v) Brij-35.
- **Enzyme:** Recombinant Human Kallikrein 14 (AcroBiosystems), 4 nM.
- **Substrate:** BOC-VPR-AMC, 100 μM.

2.8 In-gel Fluorescence

The concentrations of the target proteins and covalent probes, as well as the duration of the incubation periods, were optimised for each specific target-probe pair. Control samples were treated with an equivalent volume of DMSO. All primary incubations were performed in PBS (pH 7.4) at 37 °C and in a final reaction volume of 25 μL.

Following the initial incubation, the samples were subjected to copper-catalysed azide-alkyne cycloaddition (CuAAC) for 60 min via the addition of 2.5 μL of a freshly prepared 'click' chemistry cocktail. The final concentrations in the conjugation reactions were 100 μM TAMRA-azide, 1 mM $CuSO_4$, 1 mM TCEP and 100 μM TBTA (Tris((1-benzyl-4-triazolyl)methyl)amine). The reactions were subsequently quenched with 4X SDS Laemmli Loading Buffer with 5% β-Mercaptoethanol and denatured by heating at 95 °C for 10 min. Proteins were resolved via SDS-PAGE, and in-gel TAMRA fluorescence was visualised using the Cy3 excitation/emission channels on a Gel Doc Imaging System (Bio-Rad).

2.9 Intact Protein Mass Spectrometry

To facilitate intact mass analysis, a non-glycosylated variant of the plasma kallikrein protease domain was utilised. GenScript expressed this protein in Sf9 cells followed by purification using a HisTrap column. Specifically, to prevent heterogeneous glycosylation and eliminate unpaired thiols, the native sequence was engineered with the following substitutions: three N-linked glycosylation sites were mutated to glutamic acid (N377E, N434E, and N475E, highlighted in blue), and two specific cysteine residues were mutated to serine (C364S and C484S, highlighted in red). Prior to use, recombinant plasma kallikrein protease-domain constructs were activated by incubation with sequencing-grade trypsin (2 μg trypsin per mg protein) for 60 min at 37 °C, following the procedure described previously.[3]

^{357}NTGDNSVSTTKTSTRIVGGTESSWGEWPWQVSLQVKLTAQRHLCGGSLIGHQ
WVLTAAHCFDGLPLQDVWRIYSGILELSDITKDTPFSQIKEIIIHQNYKVSEGNHDI

ALIKLQAPLEYTEFQKPISLPSKGDTSTIYTNCWVTGWGFSKEKGEIQNILQKVNIP
LVTNEECQKRYQDYKITQRMVCAGYKEGGKDACKGDSGGPLVCKHNGMWRLV
GITSWGEGCARREQPGVYTKVAEYMDWILEK^{612}TQSSDGKAQMQSPA^{622}HHHHH
H

To evaluate covalent-adduct formation, the engineered non-glycosylated plasma kallikrein (5 μM) was incubated with a 10-fold molar excess of covalent probe in assay buffer (10 mM Tris-HCl, 150 mM NaCl, pH 7.4). The reactions were incubated at 37 °C with continuous rotation for varying time intervals. The extent of covalent modification was analysed by intact-protein mass spectrometry utilising an Agilent Q-TOF LC/MS instrument. Data processing was performed by biomolecular deconvolution using Agilent MassHunter BioConfirm Version 10.0.

2.10 Expression of Plasma Kallikrein Mutants

Target nucleophilic residues for mutagenesis were selected based on their spatial proximity to the active site of the plasma kallikrein protease domain, as determined from the published crystal structure (PDB: 2ANY). The corresponding mutant constructs (mutant residue highlighted in green) were expressed and purified by GenScript using the TurboCHO platform and HisTrap columns, respectively.

H40N

^{357}NTGDNSVSTTKTSTRIVGGTESSWGEWPWQVSLQVKLTAQRNLCGGSLIGHQ
WVLTAAHCFDGLPLQDVWRIYSGILELSDITKDTPFSQIKEIIIHQNYKVSEGNHDI
ALIKLQAPLEYTEFQKPISLPSKGDTSTIYTNCWVTGWGFSKEKGEIQNILQKVNIP
LVTNEECQKRYQDYKITQRMVCAGYKEGGKDACKGDSGGPLVCKHNGMWRLV
GITSWGEGCARREQPGVYTKVAEYMDWILEK^{612}TQSSDGKAQMQSPA^{622}HHHHH
H

Y94F

^{357}NTGDNSVSTTKTSTRIVGGTESSWGEWPWQVSLQVKLTAQRHLCGGSLIGHQ
WVLTAAHCFDGLPLQDVWRIYSGILELSDITKDTPFSQIKEIIIHQNFKVSEGNHDI
ALIKLQAPLEYTEFQKPISLPSKGDTSTIYTNCWVTGWGFSKEKGEIQNILQKVNIP
LVTNEECQKRYQDYKITQRMVCAGYKEGGKDACKGDSGGPLVCKHNGMWRLV
GITSWGEGCARREQPGVYTKVAEYMDWILEK^{612}TQSSDGKAQMQSPA^{622}HHHHH
H

K147R

^{357}NTGDNSVSTTKTSTRIVGGTESSWGEWPWQVSLQVKLTAQRHLCGGSLIGHQ
WVLTAAHCFDGLPLQDVWRIYSGILELSDITKDTPFSQIKEIIIHQEYKVSEGNHDIA
LIKLQAPLEYTEFQKPISLPSKGDTSTIYTNCWVTGWGFSKERGEIQNILQKVNIPL
VTNEECQKRYQDYKITQRMVCAGYKEGGKDACKGDSGGPLVCKHNGMWRLVGI
TSWGEGCARREQPGVYTKVAEYMDWILEKTQSSDGKAQMQSPA^{622}HHHHHH

Y172F

^{357}NTGDNSVSTTKTSTRIVGGTESSWGEWPWQVSLQVKLTAQRHLCGGSLIGHQ
WVLTAAHCFDGLPLQDVWRIYSGILELSDITKDTPFSQIKEIIIHQEYKVSEGNHDIA
LIKLQAPLEYTEFQKPISLPSKGDTSTIYTNCWVTGWGFSKEKGEIQNILQKVNIPL
VTNEECQKRFQDYKITQRMVCAGYKEGGKDACKGDSGGPLVCKHNGMWRLVGI
TSWGEGCARREQPGVYTKVAEYMDWILEKTQSSDGKAQMQSPA^{622}HHHHHH

Y175F

^{357}NTGDNSVSTTKTSTRI^{372}VGGTESSWGEWPWQVSLQVKLTAQRHLCGGSLIG
HQWVLTAAHCFDGLPLQDVWRIYSGILELSDITKDTPFSQIKEIIIHQEYKVSEGNH
DIALIKLQAPLEYTEFQKPISLPSKGDTSTIYTNCWVTGWGFSKEKGEIQNILQKVNI
PLVTNEECQKRYQDFKITQRMVCAGYKEGGKDACKGDSGGPLVCKHNGMWRLV
GITSWGEGCARREQPGVYTKVAEYMDWILEK^{622}TQSSDGKAQMQSPAHHHHHH

K192R

[357]NTGDNSVSTTKTSTRIVGGTESSWGEWPWQVSLQVKLTAQRHLCGGSLIGHQ
WVLTAAHCFDGLPLQDVWRIYSGILELSDITKDTPFSQIKEIIIHQEYKVSEGNHDIA
LIKLQAPLEYTEFQKPISLPSKGDTSTIYTNCWVTGWGFSKEKGEIQNILQKVNIPL
VTNEECQKRYQDYKITQRMVCAGYKEGGKDACRGDSGGPLVCKHNGMWRLVGI
TSWGEGCARREQPGVYTKVAEYMDWILEKTQSSDGKAQMQSPA[622]HHHHHH

S195A

[357]NTGDNSVSTTKTSTRIVGGTESSWGEWPWQVSLQVKLTAQRHLCGGSLIGHQ
WVLTAAHCFDGLPLQDVWRIYSGILELSDITKDTPFSQIKEIIIHQEYKVSEGNHDIA
LIKLQAPLEYTEFQKPISLPSKGDTSTIYTNCWVTGWGFSKEKGEIQNILQKVNIPL
VTNEECQKRYQDYKITQRMVCAGYKEGGKDACKGDAGGPLVCKHNGMWRLVG
ITSWGEGCARREQPGVYTKVAEYMDWILEKTQSSDGKAQMQSPA[622]HHHHHH

2.11 Tryptic Digest LC–MS/MS Analysis

Experimental: 2 μM plasma kallikrein was incubated with 2 μM **23** or DMSO in buffer (10 mM Tris, 150 mM NaCl, 10 mM $MgCl_2$, 1 mM $CaCl_2$, pH 7.4) for 4 h at 37 °C. The samples were reduced (5 mM TCEP, room temperature for 10 min), alkylated (10 mM iodoacetamide, room temperature for 30 min), stopped (10 mM DTT, room temperature for 15 min) and then digested overnight at 37 °C using sequencing grade trypsin (Promega, 1:10 ratio). The resulting peptides were desalted using C18 STAGE-Tips. The eluate was concentrated to dryness under vacuum and resuspended in 1% formic acid.

LC-MS/MS analysis was performed with an UltiMate3000 Nano-HPLC system (Thermo Fisher Scientific) coupled to an Orbitrap Fusion Tribrid mass spectrometer (Thermo Fisher Scientific). Samples were analyzed using an Acclaim PepMap 100 C18 trap column (0.075 × 200 mm; Thermo Fisher Scientific) and an Aurora Ultimate XT C18 UHPLC analytical emitter column (0.075 × 250 mm; Ionopticks, Collingwood, Australia) in an EASY Spray source setting with HeatSync Column Heater (Ionopticks, Collingwood, Australia). Samples were loaded onto the

trap column, washed with 0.1% TFA in water, and after 7 min transferred to the analytical column. Separation was performed at a flow rate of 400 nL min⁻^{1} (buffer A: water with 0.1% FA, buffer B: MeCN with 0.1% FA) with the following gradient: 5 % B held to 7 min (trap loading), 5→28 % B from 7 to 37 min, 28→35 % B from 37 to 42 min, a ramp to 90 % B (42.0–42.1 min) held to 52 min for column washing, and re-equilibration at 5 % B from 52.1 to 62 min (total method time 62 min). Peptides were ionized by nano-electrospray in positive mode at a spray voltage of 1.8 kV with an ion-transfer-tube temperature of 275 °C. Spectra were acquired data-dependently over a 3 s cycle with EASY-IC internal calibration. MS1 survey scans were recorded in the Orbitrap at 120,000 resolution over m/z 300–1500 (AGC target 4×10⁵, maximum injection time 50 ms, RF lens 60 %, profile mode). Precursors of charge state 4–7 above an intensity threshold of 1×10^{3} were selected (MIPS peptide filter; dynamic exclusion after 2 selections for 60 s, ±10 ppm, isotopes excluded) and isolated in the quadrupole with a 1.6 m/z window. Fragmentation was by EThcD: ETD (reaction time 25 ms, reagent target 5×10⁵, maximum reagent injection time 250 ms) with supplemental HCD activation at 30 % normalized collision energy. Product-ion scans were acquired in the Orbitrap at 60,000 resolution (AGC target 2.5×10⁵, maximum injection time 118 ms, centroid mode).

Data Analysis: Proteomics data analysis was performed using FragPipe (v23.1) with MSFragger (v4.4.1) against the plasma kallikrein sequence plus common contaminants and reversed decoys. Trypsin (stricttrypsin) was specified with up to three missed cleavages; precursor and fragment mass tolerances were 20 ppm with mass calibration and parameter optimization enabled, isotope-error 0/1/2/3, and b/y/c/z ion series. Variable modifications were carbamidomethyl (C), oxidation (M), and the expected covalent ligand **23** in its native, single- and double-oxidized forms to account for potential thioether-oxidation (Δm 1540.605 / 1556.600 / 1572.595 Da) on C, K, Y, S, T, H. PSMs were validated with PeptideProphet (Cmd line opts: --nonparam --expectscore --decoyprobs --masswidth 1000.0 --clevel -2) and proteins assembled with ProteinProphet (Philosopher; 1% protein-level FDR), and modification sites were localized with PTMProphet

using c/z and b/y ions (Cmd line opts: NOSTACK KEEPOLD STATIC FRAGPPMTOL=15 EM=1 NIONS=b,c CIONS=y,z MINPROB=0.5 C:57.02146, CK:1540.60498, CK:1556.59985, CK:1572.59485, M:15.9949). Label-free quantification used IonQuant (MS1, MaxLFQ) with match-between-runs and cross-run normalization disabled.

Covalent modification by cyclic peptide **23** was confirmed at the intact-peptide (MS1) level by extracted-ion chromatograms (XICs) generated from the MS1 survey scans using a ±10 ppm window around the monoisotopic m/z of each species. For each target peptide the unmodified ("apo", doubly-carbamidomethylated) and **23**-conjugated forms were extracted and compared between the **23**-treated and control (DMSO-treated) samples. The conjugate was detected only in the ligand-treated sample, whereas the apo peptide and total ion chromatogram (TIC) were comparable between conditions, confirming that adduct formation is dependent on ligand treatment. The most abundant **23**-conjugates were observed without carbamidomethylation on either cysteine (0×CAM). XICs were therefore extracted for these species (e.g. m/z 820.11 [4+] for the 18-mer (EGGKDACKGDSGGPLVCK) and 727.31 [4+] for the 14-mer (DACKGDSGGPLVCK)) and plotted using GraphPad Prism (see **Fig. S5**).

The site assignment was corroborated by analysis of the EThcD MS2 spectra, in which the c/z and b/y fragment-ion ladders were annotated by FragPipe (**Fig. S6**). The ligand **23** in its oxidized form was localized to Lys192 of plasma kallikrein from the EThcD spectra of the doubly-carbamidomethylated conjugate DAC(cam)K(**23**)GDSGGPLVC(cam)K (PSM probability 1.0; PTMProphet site-localization probability 0.93-0.98), in which both cysteines were carbamidomethylated (probability 1.0).

2.12 PC3 Cell Culture

PC3 cells were seeded into a T175 culture flask and cultured in Gibco™ RPMI 1640 Medium supplemented with 10% foetal bovine serum (FBS) and 1% penicillin/streptomycin. Cells were

maintained at 37 °C in humidified atmosphere of 5% CO_2 until reaching approximately 80% confluency. At 80% confluency, the growth medium was aspirated, and the cell monolayer was washed three times with PBS (pH 7.4) to remove residual serum proteins. The cells were incubated in 15 mL of serum-free Gibco™ RPMI 1640 Medium (without phenol red, supplemented with 1% penicillin/streptomycin) for 24 h at 37 °C, 5% CO_2. Following incubation, the conditioned medium was collected and centrifuged at 300 x *g* for 10 min at 4 °C to remove detached cells and debris. The cleared supernatant was collected in 1 mL aliquots, snap-frozen, and stored at -70 °C until downstream use.

2.13 Plasma Kallikrein Detection in Human Plasma

Human plasma was obtained from a single healthy donor using BD vacutainer sodium citrate tubes. Plasma was obtained following centrifugation of blood tubes at 1500 x g for 10 min at 4 °C. Plasma was then aliquoted and stored at -70 °C. The volunteer provided written informed consent, and all methods and experimental protocols were conducted in accordance with and approved by the NHS Research Ethics Committee (REC permit number 25/NI/0035). Prior to incubation with the covalent probes, the plasma was diluted 1:10 in PBS (pH 7.4).

Reaction mixtures (25 μL) were prepared using the diluted plasma, which was spiked with 1 μM blood-purified human plasma kallikrein and 1 μM **23** or FP-TAMRA. To induce contact pathway activation, 2 μg of kaolin (Thermo Fisher Scientific) was added to the plasma samples. The mixtures were incubated at 37 °C for varying time intervals. Following incubation, the samples were conjugated with TAMRA-azide using the CuAAC protocol described above, and protein labelling assessed by in-gel fluorescence.

2.14 cmABP Stability Tests

The hydrolytic stability of cmABPs was evaluated in phosphate-buffered saline (PBS, pH 7.4) at 37 °C. Each covalent peptide was prepared at a final concentration of 250 μM in the presence of

Fmoc-Tyr(OtBu)-OH (250 μM), a control which served as an internal standard for quantitative LC-MS analysis.

To capture the degradation kinetics, incubation times were sampled at 0, 4, 8, 12, 16, 24 and 48 h.

Supplementary Table 8: Timetable for probe stability analysis by LC-MS.

**Time (min)**	**Solvent A, %**<br>**(H_2O + 0.1 FA)**	**Solvent B %**<br>**(MeCN + 0.1 FA)**
0.00	95	5
10.00	95	5
100.00	5	95
105.00	5	95
110.00	95	5
120.00	95	5

The relative quantity of the remaining intact peptide at each timepoint was determined by integrating the area under the curve (AUC) of the UV signal at 254 nm and normalising it against the AUC of the internal standard. Data processing was performed using Agilent OpenLab CDS Data Analysis Software Version 2.8. To calculate the hydrolytic half-life ($t_{1/2}$), the normalised percentage of remaining intact peptide was plotted against time and fitted to a one-phase exponential decay model using GraphPad Prism.

3 Chemical Synthesis

3.1 Chemical Reagents

Supplementary Table 9: Unnatural amino acids for synthesis of cmABPs.

Cmp.	Amino Acid	Structure	Supplier
**17**	Fmoc-*m*-fluoro-L-tyrosine	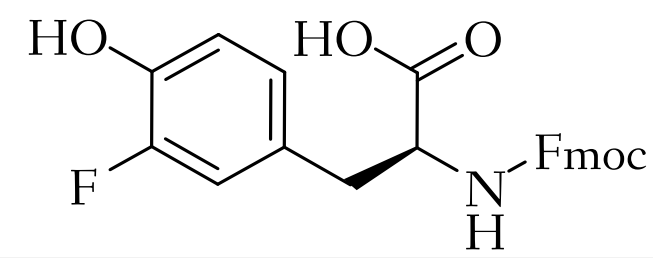	Merck
**18**	Fmoc-3,5-difluoro-L-tyrosine	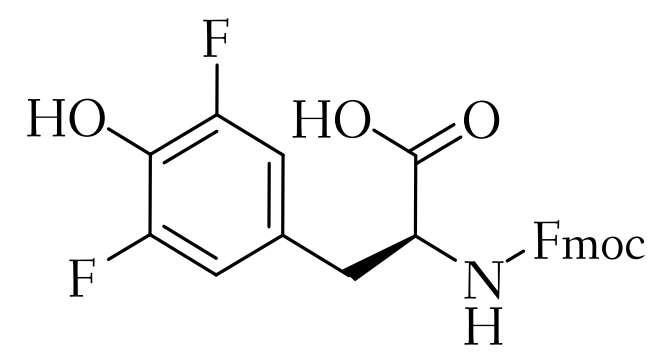	MedChem Express
**19**	Fmoc-3-nitro-L-tyrosine	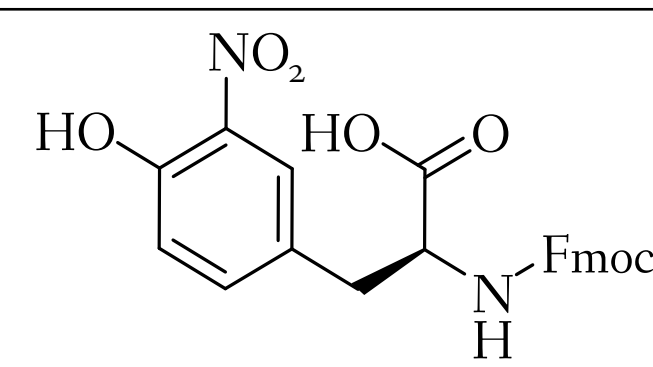	Fluorochem
**20**	Fmoc-4-hydroxyphenylglycine	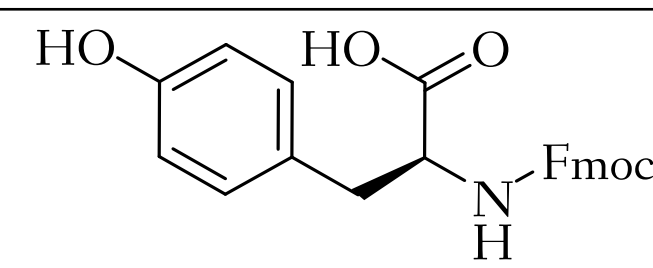	Merck
**21**	Fmoc-5-hydroxy-L-tryptophan	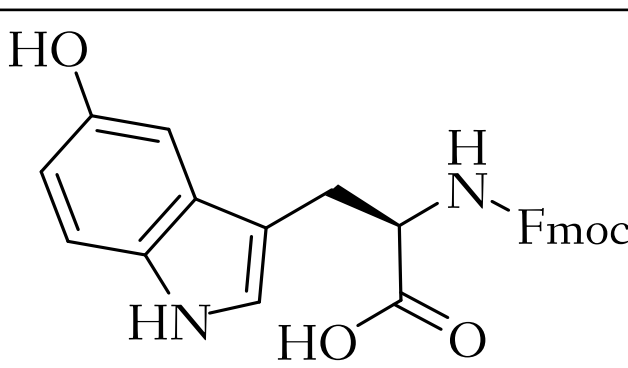	Merck
**22**	Fmoc-L-homotyrosine	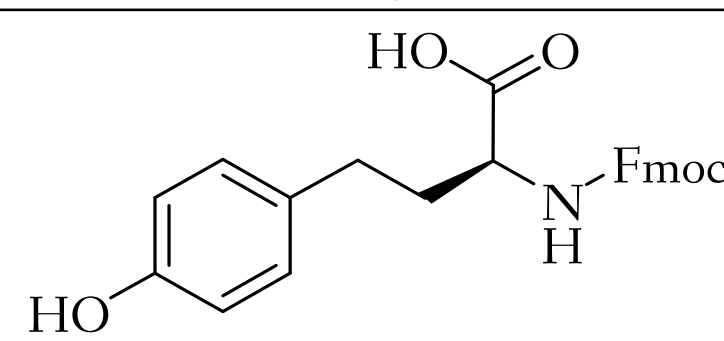	Santa Cruz. Biotech.

Fmoc-Dap(Mtt)-OH (Fluorochem), Fmoc-Cys(STmp)-OH (Merck), 5-(fluorosulfonyl)-2-methoxybenzoic acid (Enamine Ltd), AISF (Merck)

3.2 General Procedures

3.2.1 Automated Peptide Synthesis:

Peptide synthesis was performed on a 0.1 mmol scale by a Multi Pep 2 peptide synthesizer (CEM) utilising Fmoc SPPS. 150 mg of rink amide resin was pre-swelled in DCM prior to synthesis. The automated double coupling method followed repeated cycles of the following steps. Fmoc deprotection: incubation with 20% 4-methylpiperidine in DMF (1 x 5 min, then 1 x 7 min). Washing: DMF (x 7) after each deprotection. Amino acid double coupling – incubation with amino acid (0.4 mmol, 4 equiv.), HBTU (0.5 mmol, 5 equiv.) and DIPEA (0.5 mmol, 5 equiv.) in DMF (2 x 30 min), RT with shaking (350 rpm). Washing: DMF (x 3) after each amino acid double coupling. Cycles were repeated until the peptide chain was complete, followed by a final Fmoc deprotection and a final wash with ethanol.

3.2.2 Flash Chromatography:

Purification of the crude peptides was carried out using a CombiFlash NextGen 300 flash chromatography system (Teledyne ISCO) or ECS28P01 Compact Preparative System (ECOM). Separations were achieved utilising reversed-phase C18 columns (Teledyne ISCO) of varying capacities (5 g, 15 g, or 30 g), selected according to the scale of the synthesis. For closely eluting products, a ACCQ Prep 150 (Teledyne ISCO) was used with a RediSep Prep C18 column for >30 mg product or an Agilent Eclipse XDB-C18 column for <30 mg product. For all systems, the mobile phase consisted of Milli-Q H_2O (Solvent A) and HPLC-grade MeCN (Solvent B), both supplemented with 0.1% (v/v) formic acid (FA). Peptides were eluted using a standardised 25-min linear gradient from 5% to 95% MeCN. Flow rates were scaled appropriately based on column dimensions following the manufacturer's instructions. Fraction collection was guided by UV absorbance monitoring at 220 nm.

3.2.3 Analytical LC-MS:

Analysis of cmABPs was carried out on an Agilent 1260 Affinity II LC/MS coupled to a single quadrupole Agilent InfinityLab LC/MSD iQ (ESI SQ) using the following elution method: gradient of H_2O and MeCN, supplemented with 0.1% formic acid: 0-10 min 5-95% MeCN with a Jupiter 4 μm Proteo 90 Å, LC C18 column (150 x 4.6 mm). A flow rate of 1 mL/min was used.

3.2.4 Mass-Spectrometry:

High-resolution mass spectrometry was conducted using an Agilent 1260 Infinity II Quat pump HPLC coupled to a triple quadrupole Agilent 6545Q-TOF mass-spectrometer (ESI).

3.2.5 Sample Drying:

Purified peptide fractions were concentrated using a Genevac EZ-2 series evaporator. The concentrated fractions were subsequently pooled, dissolved in a mixture of MeCN and H_2O, and lyophilised to a dry powder using an Edwards Modulyo freeze-dryer.

3.3 Chemical Procedures

3.3.1 Installation of Fluorosulfate Using Fluorosulfate Tyrosine.

For **Compounds 9-16** and **31-34**, SPPS was performed (general procedure in **Section 3.2**). After incorporating Fmoc-fluorosulfate tyrosine, removal of Fmoc groups was achieved by resin treatment with 20% (v/v) 2-methylpiperidine (3 x 5 mL) in DMF for 3 min per treatment.

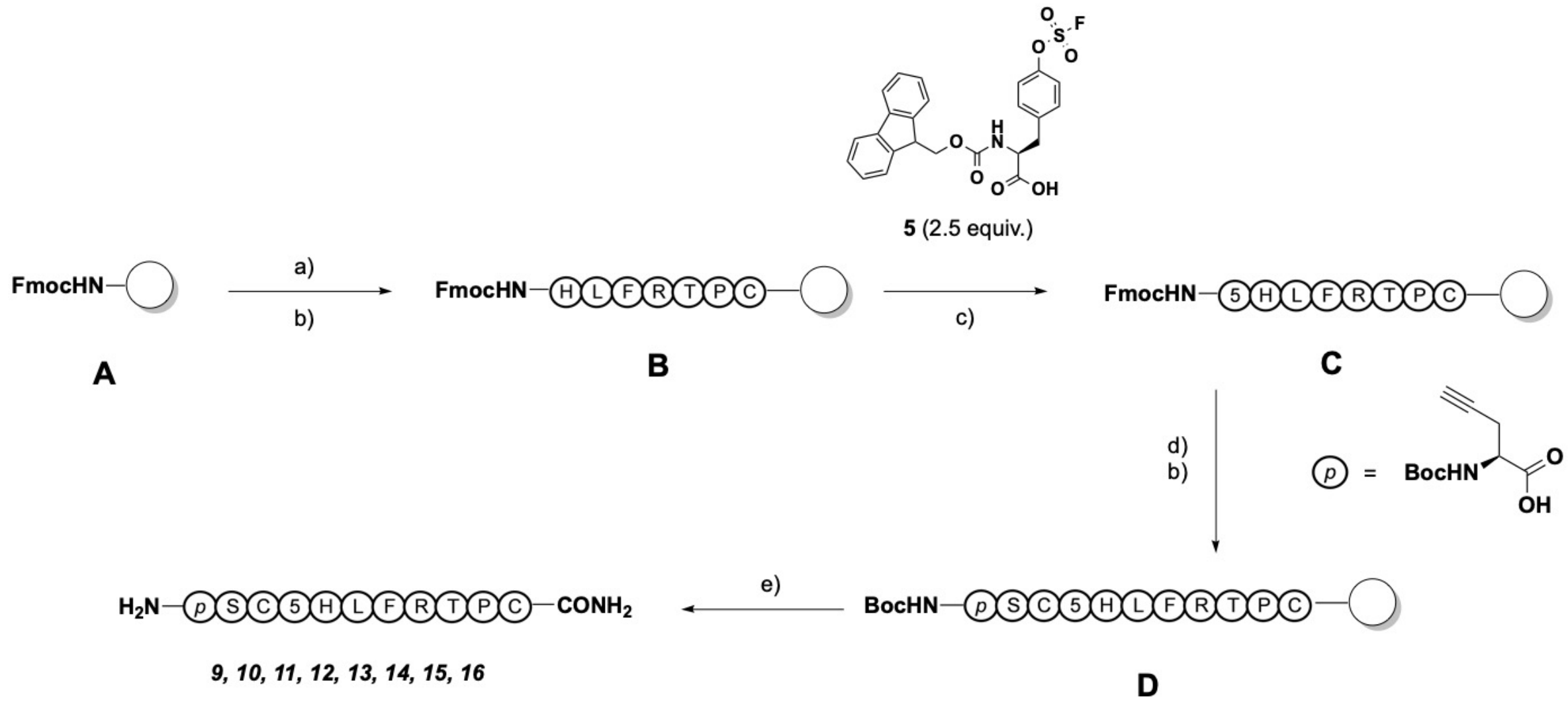

Supplementary Scheme 1: Solid-phase synthesis of compounds 9-16. *a) 20% 4-methyl piperidine, DMF, 3 x 3 min, RT. b) Fmoc-Xaa-OH (5 equiv.), HBTU (5 equiv.), DIPEA (10 equiv.), 1 h, RT. c) Fmoc-5 (2.5 equiv.), HBTU (2.5 equiv.), DIPEA, (5 equiv.), DMF, 90 min, RT. d) 20% 2-methyl piperidine, DMF, 3 x 3 min, RT. e) TFA (82.5%), H_2O (5%), thioanisole (5%), phenol (5%) and DODt (2.5%), 2 h, RT.*

3.3.2 Synthesis of Fmoc-Tyrosine Fluorosulfate

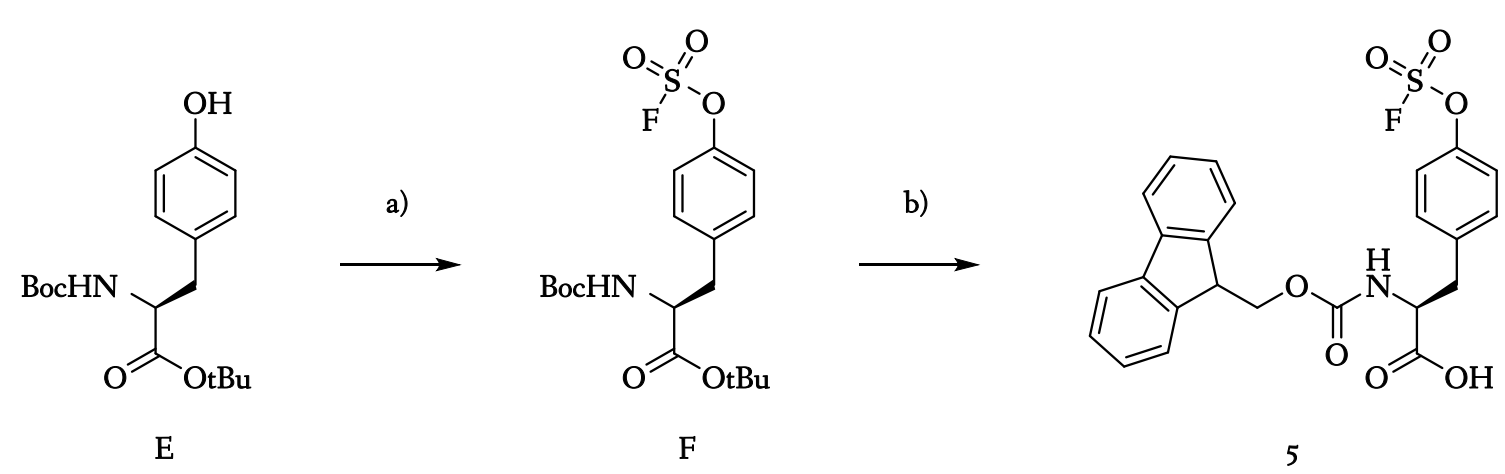

Supplementary Scheme 2: Synthesis of Fmoc-fluorosulfate tyrosine (5). Boc-Tyr(OtBu)-OH, AISF, DBU, THF, RT, 15min. b) i. 50% TFA in DCM, RT, 1 h. ii. Fmoc-succinimide, 1,4-dioxane/H_2O, RT, 1 h.

tert-Butyl(S)-2-((tert-butoxycarbonyl)amino)-3-(4-((fluorosulfonyl) oxy)phenyl)propanoate (Intermediate F)

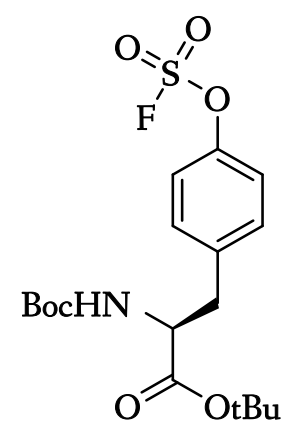

F

To a solution of *tert*-butyl (*tert*-butoxycarbonyl)-L-tyrosinate (**Intermediate E, Supplementary Scheme 2**) (1.79 g, 5.30 mmol, 1.0 equiv.) and AISF (2.00 g, 6.36 mmol, 1.2 equiv.) in THF (25 mL) was added 1,8-Diazabicyclo[5.4.0]undec-7-ene (1.74 mL, 11.70 mmol, 2.2 equiv.) (dropwise over 30 sec). Following complete addition, the reaction mixture was stirred for a further 15 min at RT before being diluted with 100 mL EtOAc and washed sequentially with 20 mL of 0.5 M HCl (x 2) and brine (x 1). The organic layer was dried ($MgSO_4$), filtered, and concentrated *in vacuo*. Purification *via* flash column chromatography on silica gel in petroleum ether/EtOAc using a gradient of 0–30% over 15 min followed by a gradient of 30–70% over 10 min afforded **Intermediate F**, as a white solid (1.60 g, 70%).

^{1}H NMR (400 MHz, DMSO-d_6) δ 7.55 – 7.39 (m, 4H), 7.25 (d, J= 8.1 Hz, 1H), 4.07 (td, J = 9.0, 5.9 Hz, 1H), 3.01 (dd, J = 13.8, 5.8 Hz, 1H), 2.91 (dd, J = 13.7, 9.8 Hz, 1H), 1.34 (s, 18H).

tert-Butyl 2-((((9H-fluoren-9-yl)methoxy)carbonyl)amino)-3-(4-((fluorosulfonyl)oxy)phenyl)propanoate (5)

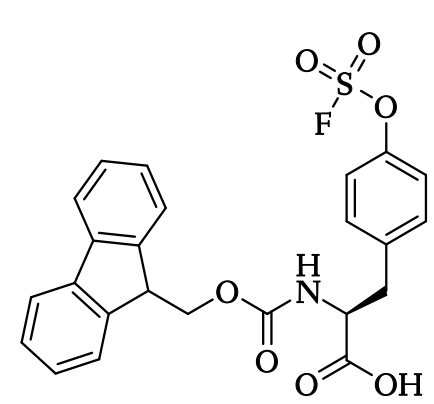

5

Intermediate F (1.12 g, 4.26 mmol, 1.0 equiv.) was dissolved in a 1:1 (v/v) mixture of DCM/TFA (20 mL) and stirred for 1 h at RT. Upon complete deprotection (monitored by TLC analysis), the reaction mixture was concentrated *in vacuo* and the resulting residue dissolved in 1:1 (v/v) 1,4-

dioxane/H_2O (20 mL). The pH was adjusted to 8.5 using $NaHCO_3$ and then Fmoc-OSu (1.43 g, 4.26 mmol, 1.0 equiv.) was added and stirred at RT for 1 h. The reaction mixture was acidified to pH 2 with 1 M HCl and extracted with 50 mL EtOAc (x3). The combined organic layers were washed with 20 mL of 1 M HCl (x 2) and brine (x 1), dried ($MgSO_4$), filtered, and concentrated *in vacuo*. Purification *via* flash column chromatography on silica gel in MeOH/DCM using a gradient of 0–5% afforded **5** as a white solid (1.05 g, 51%).

^{1}H NMR (400 MHz, DMSO-d_6) δ 12.82 (s, 1H), 7.89 (d, J = 7.5 Hz, 2H), 7.78 (d, J = 8.5 Hz, 1H), 7.64 (dd, J = 7.6, 3.3 Hz, 2H), 7.49 (d, J = 1.9 Hz, 3H), 7.42 (td, J = 7.4, 2.1 Hz, 2H), 7.38 – 7.25 (m, 2H), 5.76 (d, J = 0.7 Hz, 1H), 4.28 – 4.13 (m, 4H), 3.17 (dd, J = 13.9, 4.4 Hz, 1H), 2.94 (dd, J = 13.9, 10.8 Hz, 1H).

3.3.3 DBMB/TBMB Peptide Cyclisation

To a solution of purified linear peptide (30 mg) in DMF (1 mL) was added α,α'-dibromo-*m*-xylene (DBMB) (1.5 equiv.) or 1,3,5-tris(bromomethyl)benzene (TBMB) (1.5 equiv.), followed by DIPEA (4 equiv.). The reaction mixture was stirred at 37 °C for 30 min, and the progress of reaction monitored by LC-MS analysis. Upon completion, the crude reaction was concentrated *in vacuo* and purified by reversed-phase flash column chromatography on C18 silica gel. The purified cyclic peptides were then lyophilised and subsequently prepared in DMSO (10 mM) followed by storage at –80 °C.

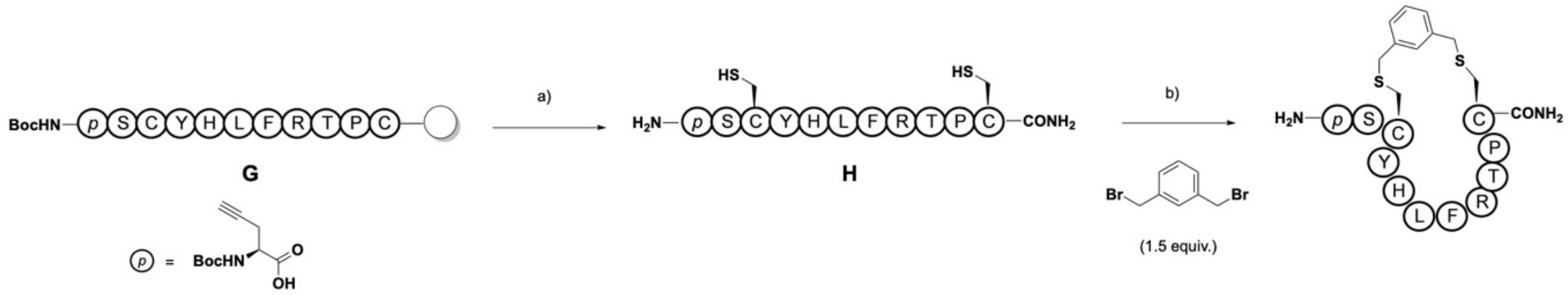

***Supplementary Scheme 3: DBMB cyclisation**. a) TFA (82.5%), H_2O (5%), thioanisole (5%), phenol (5%) and DODt (2.5%), 2 h, RT. b) DBMB or TBMB (1.5 equiv.), DIPEA (4 equiv.), DMF, 30 min, 37 °C. p* = propargyl glycine

3.3.4 Installation of Fluorosulfate Using AISF

For **Compounds 17–22**, incorporation of the Fmoc-Tyr-OH or unnatural amino acids (see **Supplementary Table** 8) was performed using conditions in **Section 3.2.1**, followed by deprotection with 20% (v/v) 4-methylpiperidine in DMF (3 x 5 mL washes, 3 min per treatment). The resin was then washed sequentially with DMF (2 x 5 mL), DCM (2 x 5 mL), and DMF (2 x 5 mL). A solution of 4-(acetylamino)phenylimidodisulfuryl difluoride (3 equiv.) in THF (4 mL) was then added, followed by 1,8-diazabicyclo[5.4.0]undec-7-ene (4 equiv.). The resin was agitated at RT for 30 min before being washed sequentially with DMF (x 2), DCM (x 2), and DMF (x 2). Following resin cleavage, the incorporation of the fluorosulfate group was confirmed by LC/MS analysis.

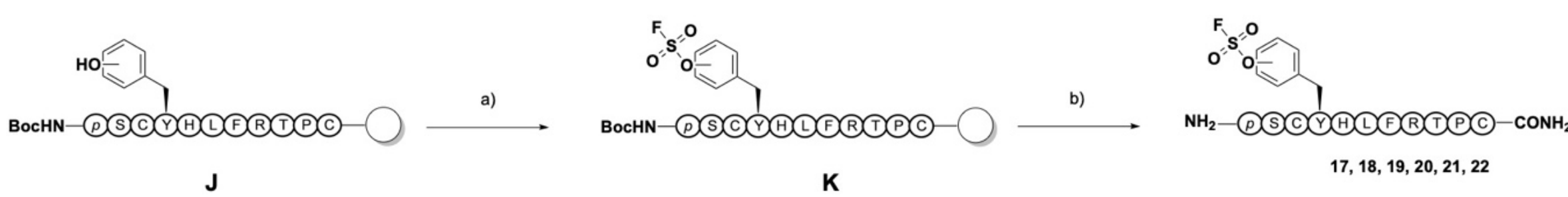

Supplementary Scheme 4: Solid-phase synthesis of compounds 17-22. *a) AISF (3 equiv.), DBU (4 equiv.), THF, 30 min, RT. b) TFA (82.5%), H_2O (5%), thioanisole (5%), phenol (5%) and DODt (2.5%), RT, 2 h.*

3.3.5 Installation of Sulfonyl Fluoride Using 5-(Fluorosulfonyl)-2-methoxybenzoic acid

For **Compounds 23–24**, selective deprotection of incorporated Dap(Mtt)-OH was performed on resin following chain completion. The Mtt protecting group was removed using 3% (v/v) TFA and 5% (v/v) triisopropylsilane (TIS) in DCM (3 x 5 mL, 15 min per treatment; the resin was washed with 2 × 5 mL DCM between treatments). After deprotection, the resin was washed sequentially with DMF (x 2), DCM (x 2), and DMF (x 2). The resin was then treated with 5-(fluorosulfonyl)-2-methoxybenzoic acid (1.5 equiv.), DIPEA (3 equiv.), and PyBOP (3 equiv.) in 2 mL DMF and agitated for 1 h at RT. The resin was then washed with DMF (x 2), DCM (x 2), DMF (x 2), and DCM (x 2). Sulfonyl fluoride warhead incorporation was confirmed *via* LC-MS analysis.

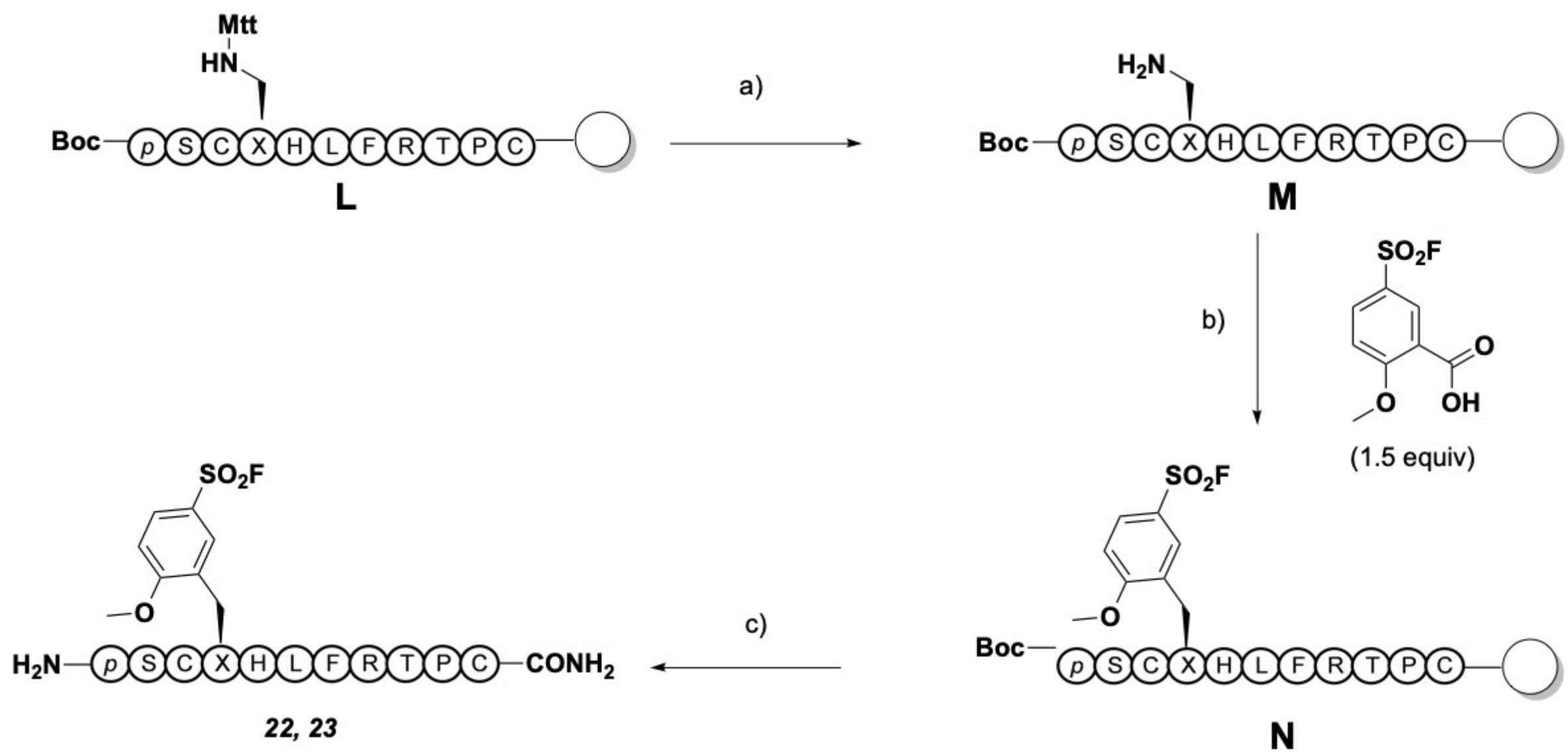

Supplementary Scheme 5: Solid-phase synthesis of compounds 23-24. *a) 3% (v/v) TFA, 5% (v/v) TIS, DCM, 3 x 15 min, RT. b) 5-(Fluorosulfonyl)-2-methoxybenzoic acid (1.5 equiv.), PyBOP (3 equiv.), DIPEA (3 equiv.), 2 mL DMF, 60 min, RT. c) TFA (82.5%), H_2O (5%), thioanisole (5%), phenol (5%) and DODt (2.5%) 2 h, RT.*

3.3.6 Installation of Sulfonyl Fluoride Using 3/4/5-Benzylsulfonyl Fluoride

For **Compounds 25–27**, selective deprotection of incorporated Cys(STmp)-OH was performed on resin by treatment with 5% (w/v) DTT and *N*-methylmorpholine (NMM) (0.1 M) in DMF (5 mL, 3 x 5 min per treatment; the resin was washed with DMF (3 × 5 mL) between each treatment). After completion, the resin was washed with DMF (2), DCM (x 2), and DMF (x 2). The resin was then treated with a solution of substituted (bromomethyl)benzenesulfonyl fluoride (1.2 equiv.) and DIPEA (2.5 equiv.) in DMF (5 mL), followed by agitation at RT for 30 min. Incorporation of the sulfonyl fluoride warhead was confirmed *via* LC-MS analysis.

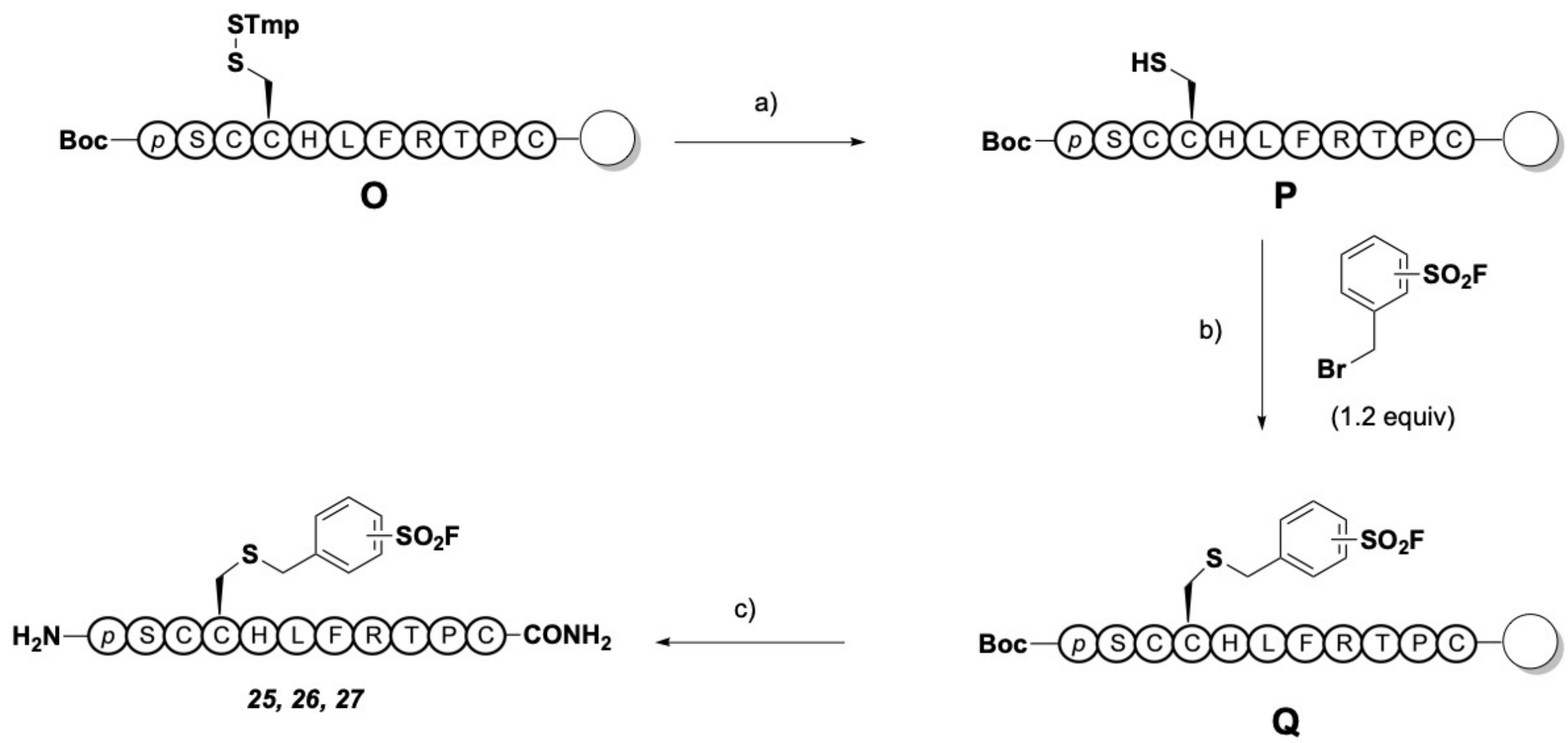

Supplementary Scheme 6: Solid-phase synthesis of compounds 25-27. *a) 5% (v/v) DTT, 0.1 M N-methylmorpholine, DMF, 3 x 5 min, RT. b) (bromomethyl)benzenesulfonyl fluoride (1.2 equiv.), DIPEA (2.5 equiv.), DMF, 1 h, RT. c) TFA (82.5%), H_2O (5%), thioanisole (5%), phenol (5%) and DODt (2.5%), 2 h, RT.*

3.3.7 Synthesis of Diphenylphosphonate 29

2-Chlorotrityl chloride resin (150 mg, loading 1.22 mmol/g) was swollen in DCM for 10 min, followed by washing with DCM (3 x 5 mL). Fmoc-Phe-OH (5 equiv.) and DIPEA (10 equiv.) in DCM was then added and the reaction mixture was agitated for 2 h at RT. After 2 h, MeOH (500 μL) was added and the mixture agitated for a further 30 min at RT. The resin was then washed sequentially with DMF (x 2), DCM (x 2), and DMF (x 2). Removal of the Fmoc group was achieved by treating the resin with a 20% (v/v) solution of 4-methylpiperidine in DMF (3 x 5 mL, 3 min per treatment). The resin was then sequentially washed with DMF (x 2), DCM (x 2), and DMF (x 2).

Fmoc-Leu-OH, Fmoc-His(Trt)-OH and Fmoc-Aminohexanoic acid (Fmoc-Ahx-OH_ were coupled to the resin using standard SPPS procedures as described above. The peptide was capped with fluorescein (3 equiv.), DIC (3 equiv.) and Oxyma (3 equiv.) in 2 mL DMF overnight at RT, washed with DMF (x 2), DCM (x 2), DMF (x 2) and cleaved from the resin by treatment with 2

mL of a 1:3 (v/v) mixture of HFIP:DCM for 2 h at RT. The resultant filtrate was concentrated *in vacuo* followed by purification *via* reversed-phase flash column chromatography on C18 silica gel to afford **Intermediate W** (**Supplementary Scheme 8**).

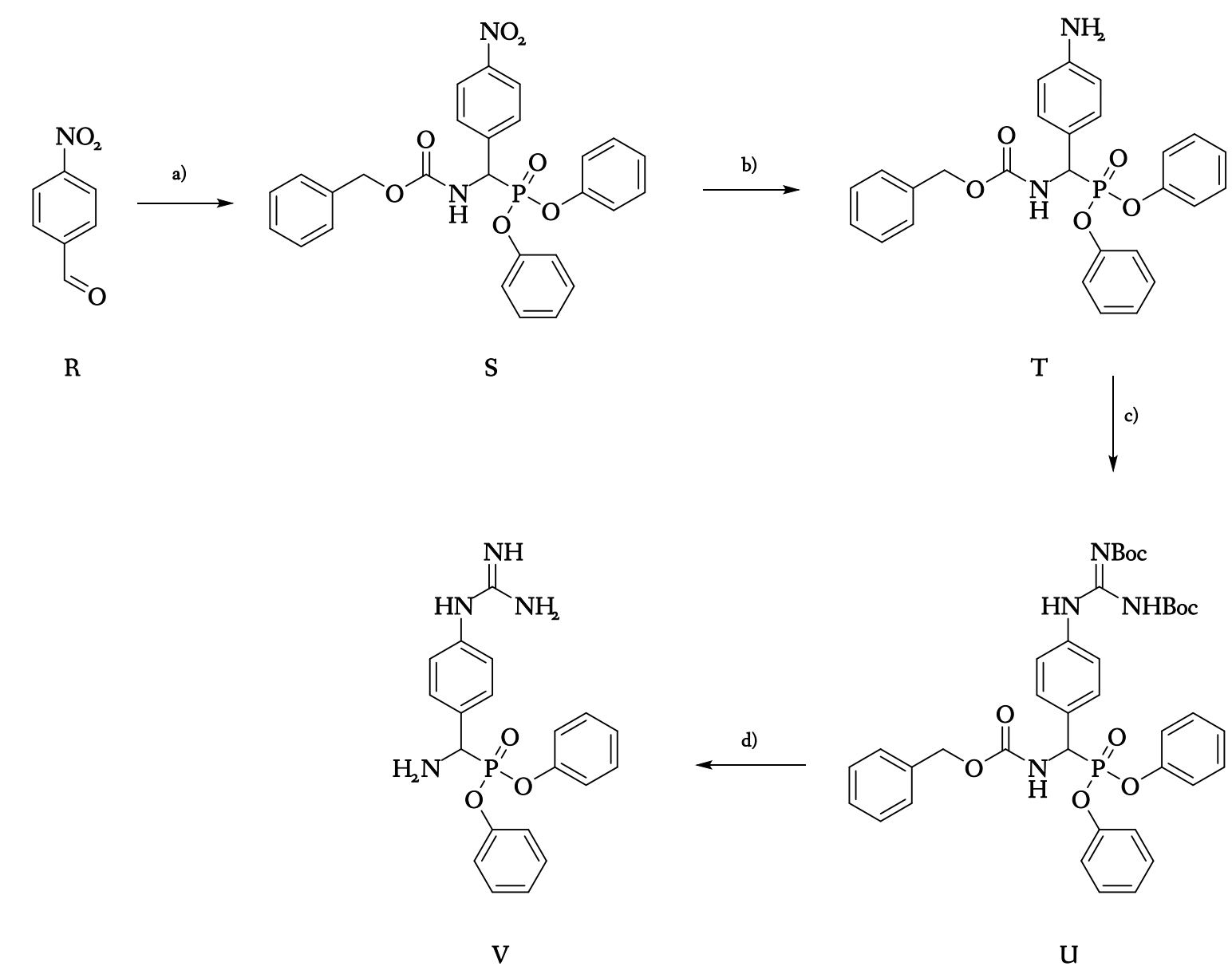

Supplementary Scheme 7: Synthesis of Diphenylphosphonate warhead. a) 4-nitrobenzaldehyde, $P(OPh)_3$, benzyl carbamate, AcOH, 90 °C, 2 h. b) Fe, AcOH, 70 °C, 2 h. *c. N,N'*-bis-Boc-1-guanylpyrazole, NEt_3, DCM, RT, 16 h. d) 33% HBr in AcOH, RT, 2 h.

Benzyl ((diphenoxyphosphoryl)(4-nitrophenyl)methyl)carbamate (Intermediate S)

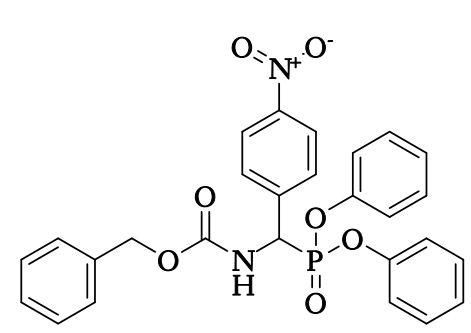

S

A solution of 4-nitrobenzaldehyde (**Intermediate R, Supplementary Scheme 7**) (0.91 g, 6.00 mmol, 1.5 equiv.), benzyl carbamate (1.24 g, 4.00 mmol, 1.0 equiv.), and triphenyl phosphite (1.05 mL, 4.00 mmol, 1.0 equiv.) in glacial acetic acid (1.2 mL) was stirred at 90 °C for 2 h. Upon completion (as monitored by TLC), the mixture was concentrated *in vacuo* and recrystalised from MeOH (washed with ice-cold 5 mL of MeOH and Et_2O) to afford **Intermediate S** as a white solid (1.16 g, 56%).

^{1}H NMR (500 MHz, DMSO-d_6) δ 9.09 (d, J = 10.1 Hz, 1H), 8.27 (d, J = 8.5 Hz, 2H), 7.94 (dd, J = 8.9, 2.2 Hz, 2H), 7.40 – 7.28 (m, 9H), 7.20 (td, J = 7.4, 3.9 Hz, 2H), 7.04 (dd, J = 16.7, 8.0 Hz, 4H), 5.91 – 5.81 (m, 1H), 5.14 (d, J = 12.5 Hz, 1H), 5.07 (d, J = 12.4 Hz, 1H).

Benzyl (*R*)-((4-aminophenyl)(diphenoxyphosphoryl)methyl)carbamate (Intermediate T)

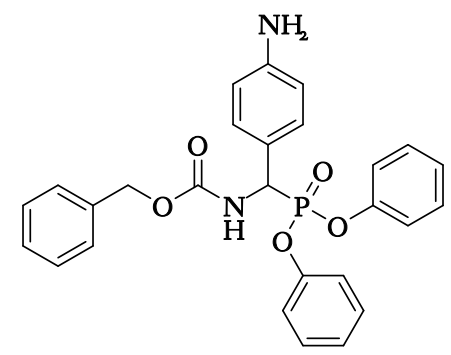

T

Intermediate S (3.00 g, 6.00 mmol, 1.0 equiv.) and iron powder (3.00 g, 53.7 mmol, 9.0 equiv.) was dissolved in glacial acetic acid (24 mL) and stirred at 70 °C for 2 h. The reaction mixture was concentrated *in vacuo* and the crude residue was suspended in 50 mL EtOAc. The suspension was centrifuged for 5 min at 3000 rpm and the supernatant was collected and concentrated *in vacuo* to afford **Intermediate T** as a brown solid (2.61 g, 5.34 mmol, 89%) which was carried forward without further purification.

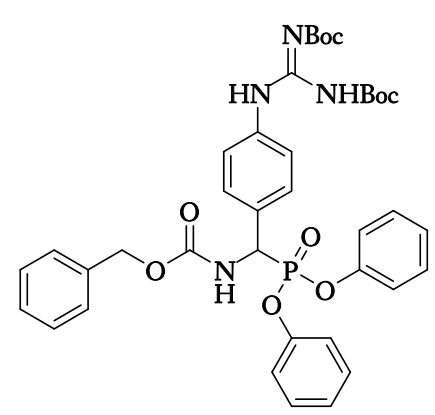

U

***ii*: Guanylation.** To a solution of **Intermediate T** (2.61 g, 5.34 mmol, 1.0 equiv.) in DCM (30 mL) was added *N,N*-bis-Boc-1-guanylpyrazole (1.66 g, 5.34 mmol, 1.0 equiv.) and Et_3N (1.49 mL, 10.70 mmol, 2.0 equiv.) which was allowed to stir at RT overnight. Following completion, the reaction mixture was concentrated *in vacuo*, dissolved in 50 mL EtOAc, and washed sequentially with 20 mL of 1 M HCl, sat. aq. $NaHCO_3$, and brine. The organic layer was dried ($MgSO_4$), filtered, and concentrated *in vacuo*. Purification *via* flash column chromatography on silica gel in 40-60° petroleum ether/EtOAc using a gradient of 10–100% afforded **Intermediate U** as a white solid (1.80 g, 45% over two steps).

^{1}H NMR (500 MHz, DMSO-d_6) δ 11.37 (s, 1H), 10.00 (s, 1H), 8.86 (d, J = 10.1 Hz, 1H), 7.57 (q, J = 8.7 Hz, 4H), 7.33 (p, J = 8.1 Hz, 8H), 7.18 (t, J = 7.4 Hz, 2H), 7.03 (d, J = 8.0 Hz, 2H), 6.96 (d, J = 8.0 Hz, 2H), 5.61 – 5.51 (m, 1H), 5.12 (d, J = 12.7 Hz, 1H), 5.04 (d, J = 12.3 Hz, 1H), 1.49 (s, 9H), 1.38 (s, 9H).

Diphenyl (amino(4-guanidinophenyl)methyl)phosphonate (Intermediate V)

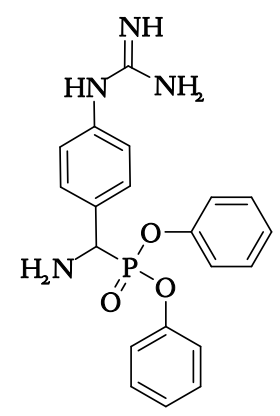

V

Intermediate U (0.75 g, 1.02 mmol) was treated with a 33% (w/w) solution of HBr in acetic acid (5 mL) and allowed to stir for 2 h at RT. After 2 h, the reaction mixture was concentrated *in vacuo* followed by purification by recrystallization from MeOH/Et_2O to afford **Intermediate V** as a white solid (0.34 g, 83%).

^{1}H NMR (500 MHz, DMSO-d_6) δ 9.88 (s, 1H), 9.41 (s, 3H), 7.73 (dd, J = 8.7, 2.1 Hz, 2H), 7.55 (s, 4H), 7.38 (dq, J = 15.3, 7.8 Hz, 5H), 7.24 (dt, J = 14.9, 7.4 Hz, 2H), 7.14 (d, J = 7.9 Hz, 2H), 7.02 (d, J = 8.0 Hz, 2H), 5.75 – 5.65 (m, 1H).

Diphenyl Phosphonate ABP (29)

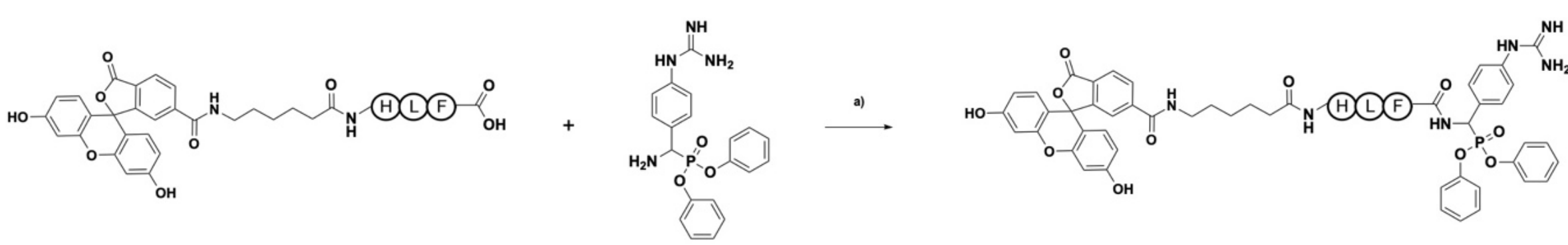

W V 29

Supplementary Scheme 8: Synthesis of 29. *a) HATU, 2,4,6-trimethylpyridine, 2 h, RT.*

To a solution of **Intermediate W,** (**Supplementary Scheme** 8) (25.0 mg, 29.7 μmol, 1 equiv.) in DMF (1 mL) was added HATU (11.3 mg, 29.7 μmol, 1 equiv.) and 2,4,6-trimethylpyridine (19.6 μL, 148.5 μmol, 5 equiv.). The reaction mixture was stirred for 3 min, then **Intermediate V** (**Supplementary Scheme** 8) (14.2 mg, 35.6 μmol, 1.2 equiv.) was added and the reaction mixture was stirred for 2 h at RT. After completion (as monitored by LC-MS analysis), the reaction mixture was concentrated *in vacuo* and the resulting residue was then treated with a 95:2.5:2.5 solution of TFA/TIS/H_2O (1 mL) and stirred for 2h at RT. The reaction

mixture was concentrated *in vacuo* and purification *via* reversed-phase flash column chromatography on C_{18} silica gel afforded **29** (2.00 mg, 6%).

4 Supplementary Figures

4.1 Volcano Plot of NGS Data

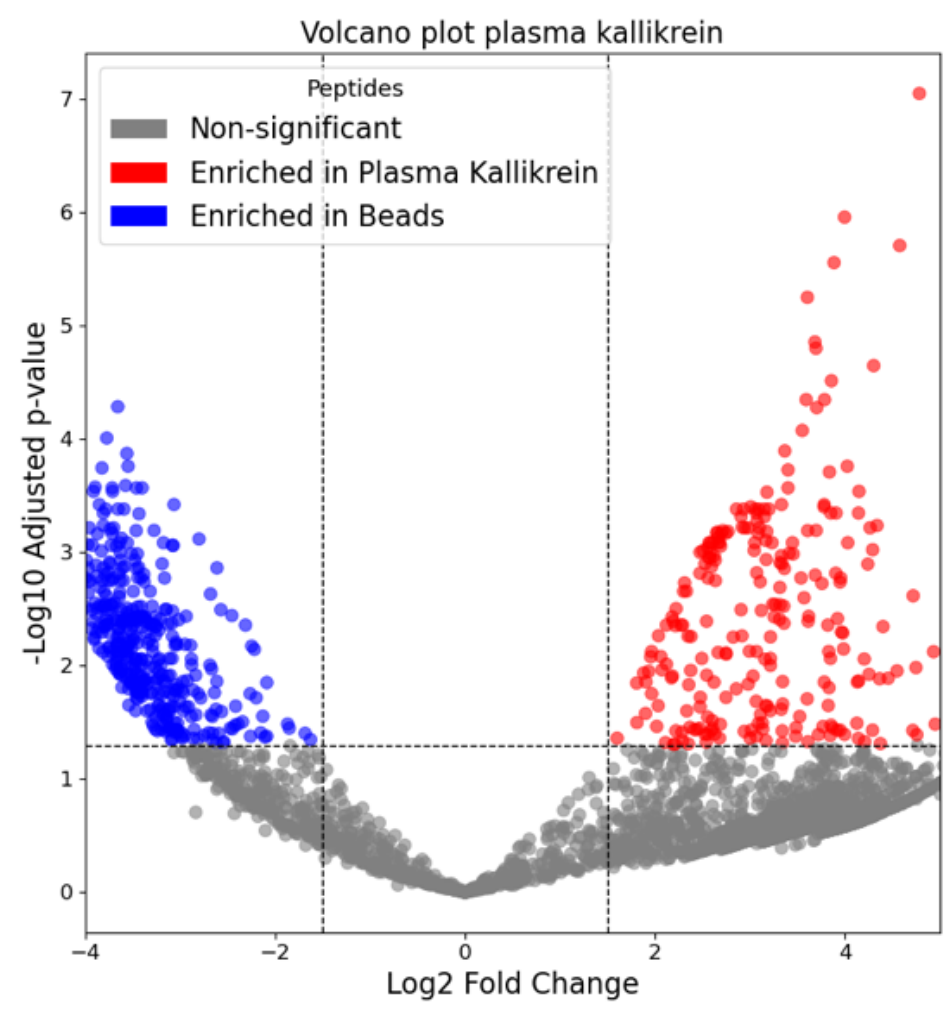

Supplementary Figure 1: Volcano plot showing enrichment of peptides from phage display against plasma kallikrein relative to a streptavidin bead control. *The x-axis represents log₂ fold change in peptide frequency, and the y-axis shows statistical significance (–log₁₀ adjusted p-value). Dashed lines indicate the significance thresholds (adj. p < 0.05, log₂FC > 1.5). Peptides enriched in plasma kallikrein and control samples are highlighted in red and blue, respectively; non-significant peptides are shown in grey.*

4.2 Labelling of Plasma Kallikrein Mutants with FP-TAMRA

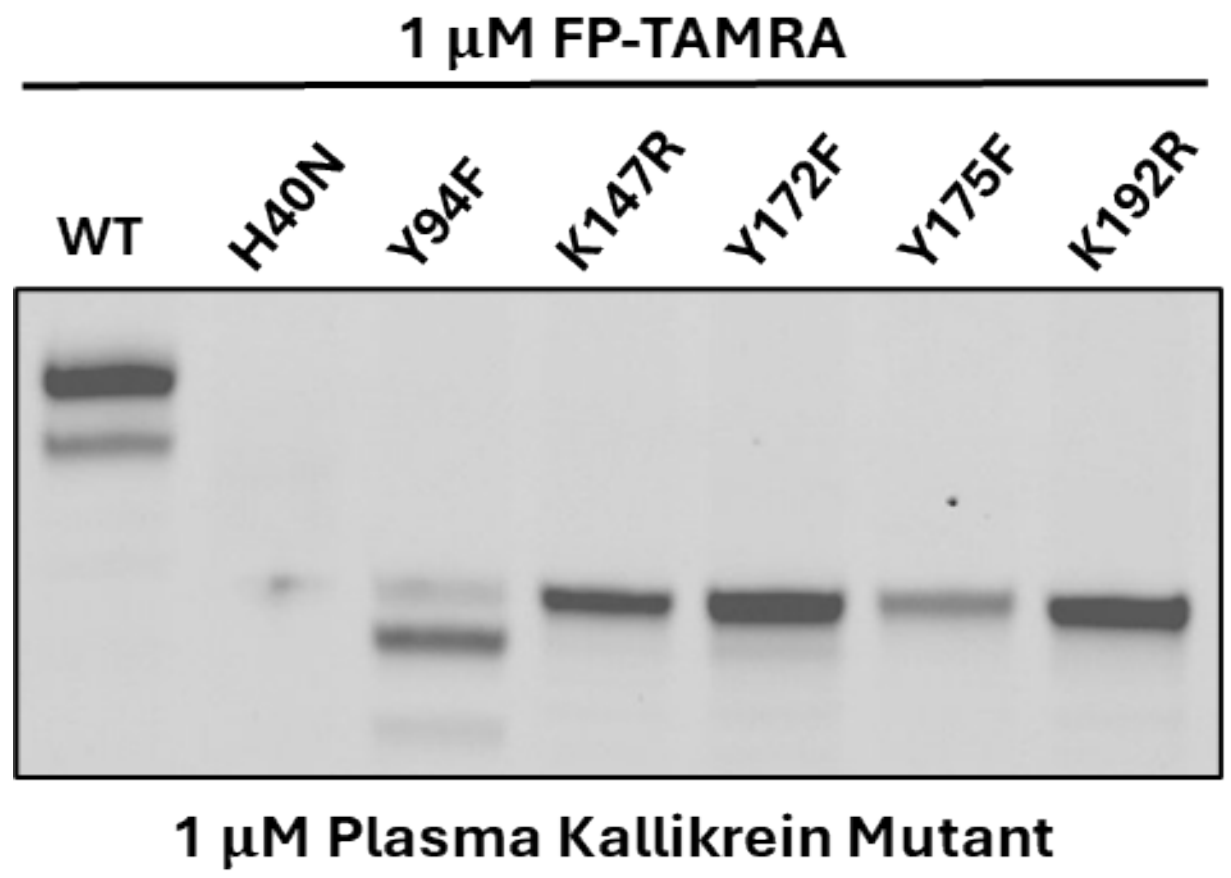

Supplementary Figure 2: Labelling of plasma kallikrein mutants by FP-TAMRA. *Mutants (1 μM) were incubated with the broad-spectrum activity-based probe FP-TAMRA (1 μM) in PBS (pH 7.4) for 60 min at 37 °C. Following SDS-PAGE resolution, covalent labelling was visualised via in-gel TAMRA fluorescence.*

4.3 Inhibition Constant Plots for 10 and 12

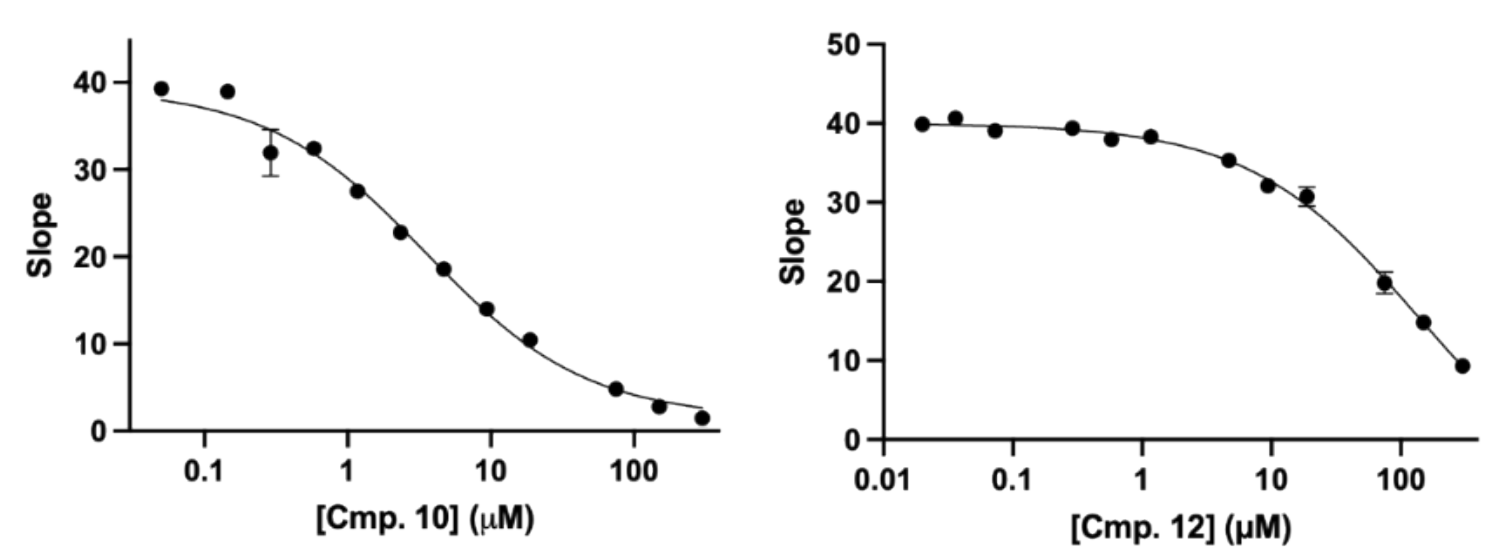

Compound	K_i (µM)
10	3.53 ± 0.7
12	>100

Supplementary Figure 3: Determination of apparent inhibition constants ($K_{i,app}$) for compounds 10 and 12. *Dose-response curves for the residual enzymatic activity of human plasma kallikrein in the presence of varying concentrations of compound 10 (left) and 12 (right). Enzymatic activity is represented by the slope of the kinetic progress curves. Data points indicate the mean ± SD of three independent replicates. Solid lines represent the non-linear regression fit to a standard dose-*

response model using GraphPad Prism. The accompanying table summarises the calculated ($K_{i,app}$) values for each compound.

4.4 Enzyme Activity Assay of 23 with WT and K192R PKa

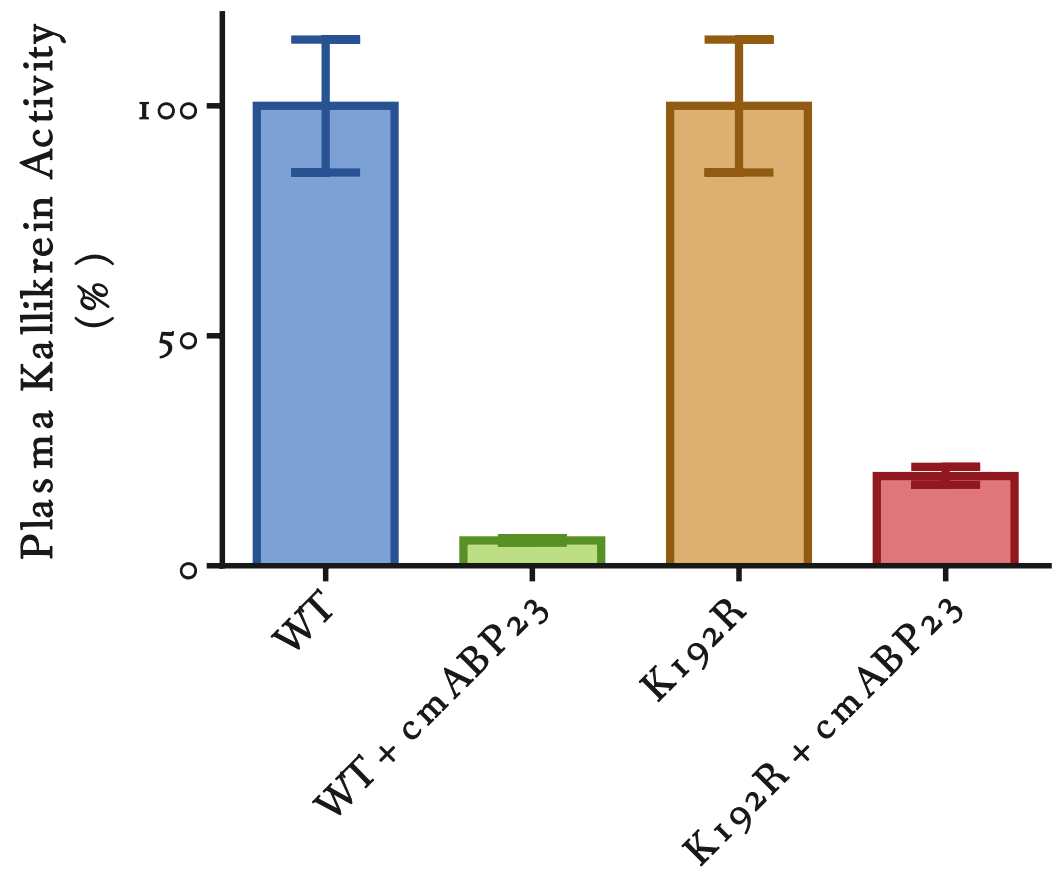

Supplementary Figure 4: Inhibition of wild-type and K192R plasma kallikrein by 23. *Wild-type or K192R plasma kallikrein was incubated with **23** (20 μM) for 60 min at 37 °C, and residual enzymatic activity was measured relative to the corresponding untreated control.*

4.5 Tryptic-Digest LC-MS/MS

a.

Peptide species	Mono. mass (Da)	z=2	z=3	z=4	z=5	z=6	z=7	Observation
18-mer apo (2×CAM): EGGKDAC(cam)KGDSGGPLV C(cam)K	1833.8349	917.9247	612.2856	459.4660	367.7743	306.6464	262.9837	both samples; RT ≈ 17.1 min
18-mer +**23** (oxidized, 0×CAM): EGGKDACKGDSGGPLVCK + **23**	3276.3919	1639.2032	1093.1379	820.1052	656.2856	547.0726	469.0633	**23**-treated only; RT ≈ 29.5 min
14-mer apo (2× CAM): DAC(cam)KGDSGGPLVC(cam)K	1462.6544	732.3345	488.5587	366.6709	293.5382	244.7830	209.9579	both samples; RT ≈ 18.9 min
14-mer +**23** (oxidized, 0×CAM): DACKGDSGGPLVCK + **23**	2905.2114	1453.6130	969.4111	727.3101	582.0496	485.2092	416.0375	**23**-treated only; RT ≈ 31.2 min

b.

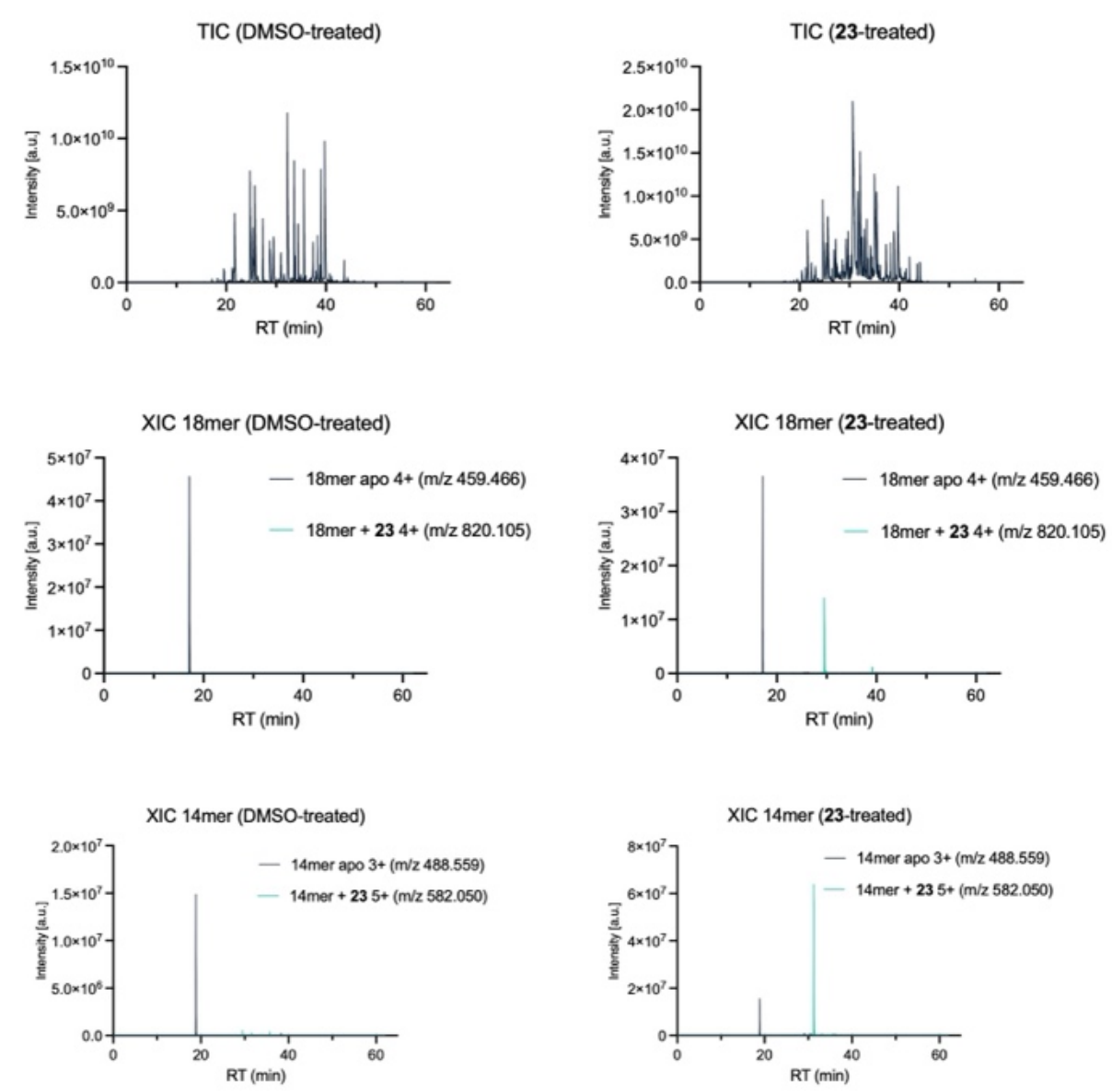

Supplementary Figure 5: *MS1 analysis of apo and **23**-conjugated plasma kallikrein peptides. (**a**) Summary of the expected monoisotopic masses for different charge states for unmodified and **23**-modified peptide ions, with identified signals highlighted in blue (CAM/cam = carbamidomethylation). (**b**) Total-ion chromatograms (TICs) and extracted-ion chromatograms (XICs) for DMSO (control)- and **23**-treated samples, showing detection of ions corresponding to the unmodified apo peptides in both samples, whereas ions corresponding to the oxidized **23**-modified peptides were detected exclusively in the **23**-treated sample.*

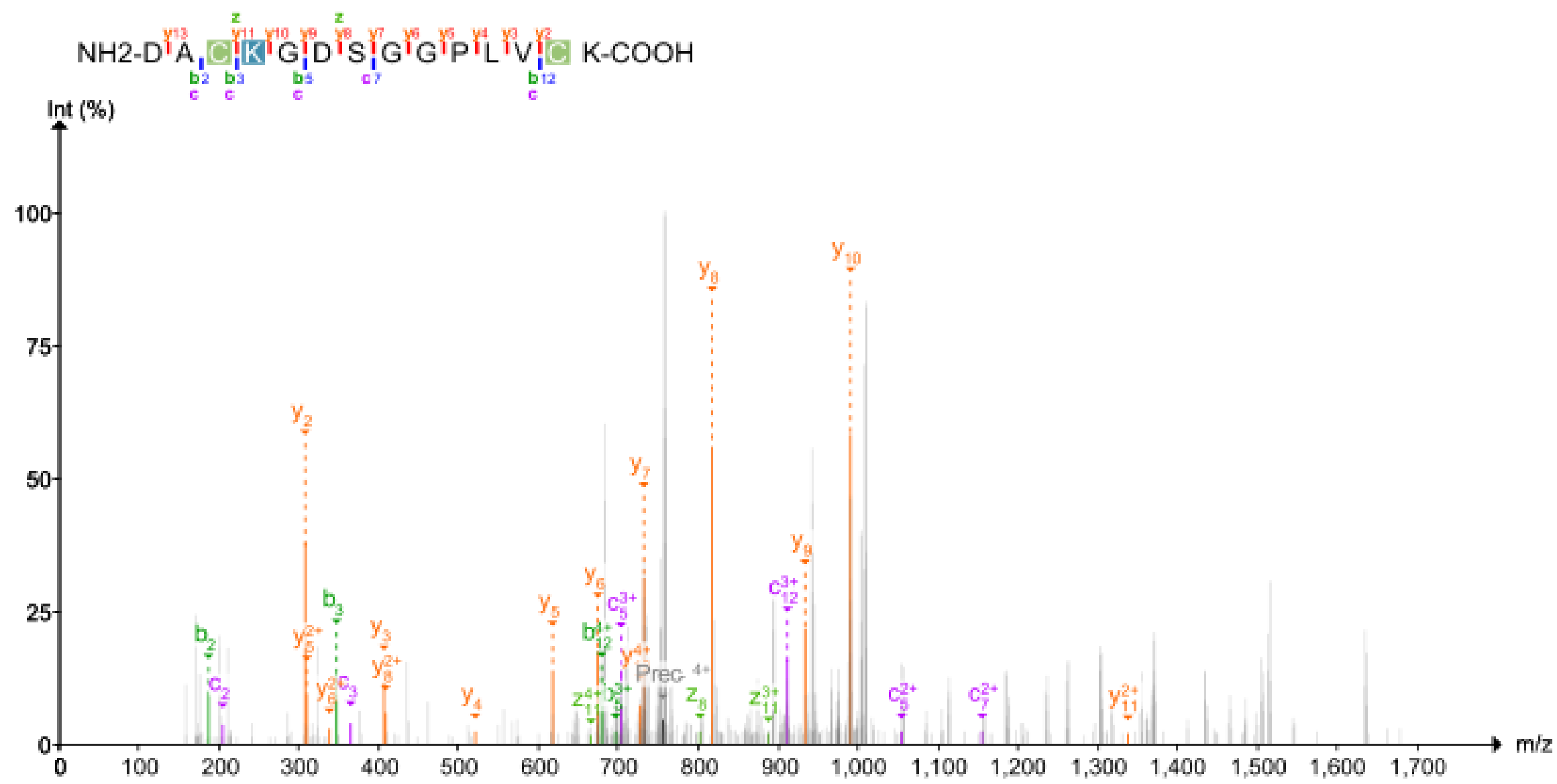

Supplementary Figure 6: **FragPipe (MSFragger) analysis identifies Lys192 as the residue modified by 23.** Representative EThcD MS2 spectrum of the tryptic 14-mer peptide (residues 182–195) in its doubly-carbamidomethylated (cam), **23**-conjugated form, DAC(cam)K(**23**)GDSGGPLVC(cam)K, carrying the mono-oxidized form of **23**. Both cysteines (Cys184 and Cys194) are carbamidomethylated (site probability 1.0), excluding a cysteine linkage, while PTMProphet localizes **23** to the single lysine (Lys192) with a probability of 0.93–0.98 across replicate EThcD spectra (PSM probability 1.0). The b/y and c/z fragment-ion ladders are consistent with this assignment.

4.6 Intact-protein mass spectrometry analysis of covalent modification kinetics for cyclic and linear probe.

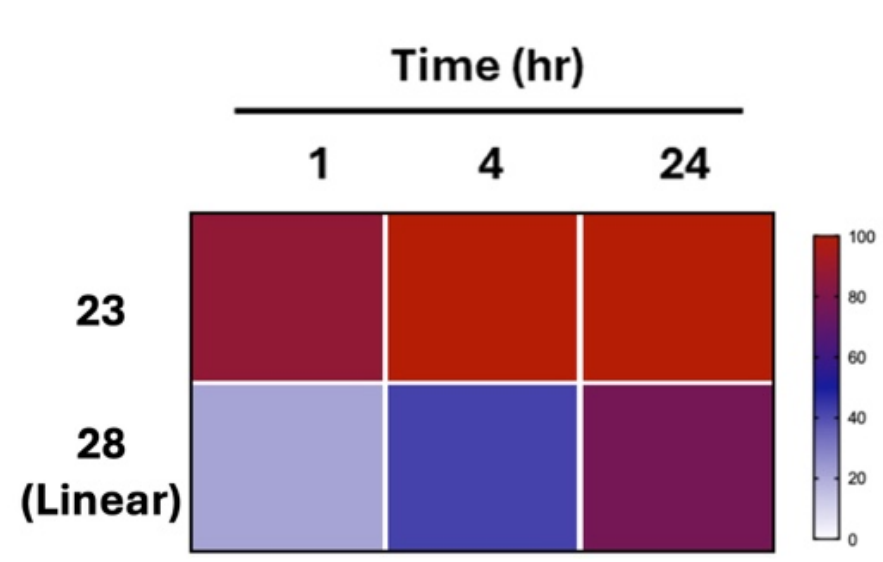

Supplementary Figure 7: Intact-protein mass spectrometry analysis of covalent modification kinetics for 23 and 28. *50 μM of probe* ***23*** *(cyclized) or* ***28*** *(linear) incubated with 5 μM PKa for 1, 4 or 24 h at 37 °C.*

4.7 In-gel Fluorescence Profiling Against Serine Protease Panel

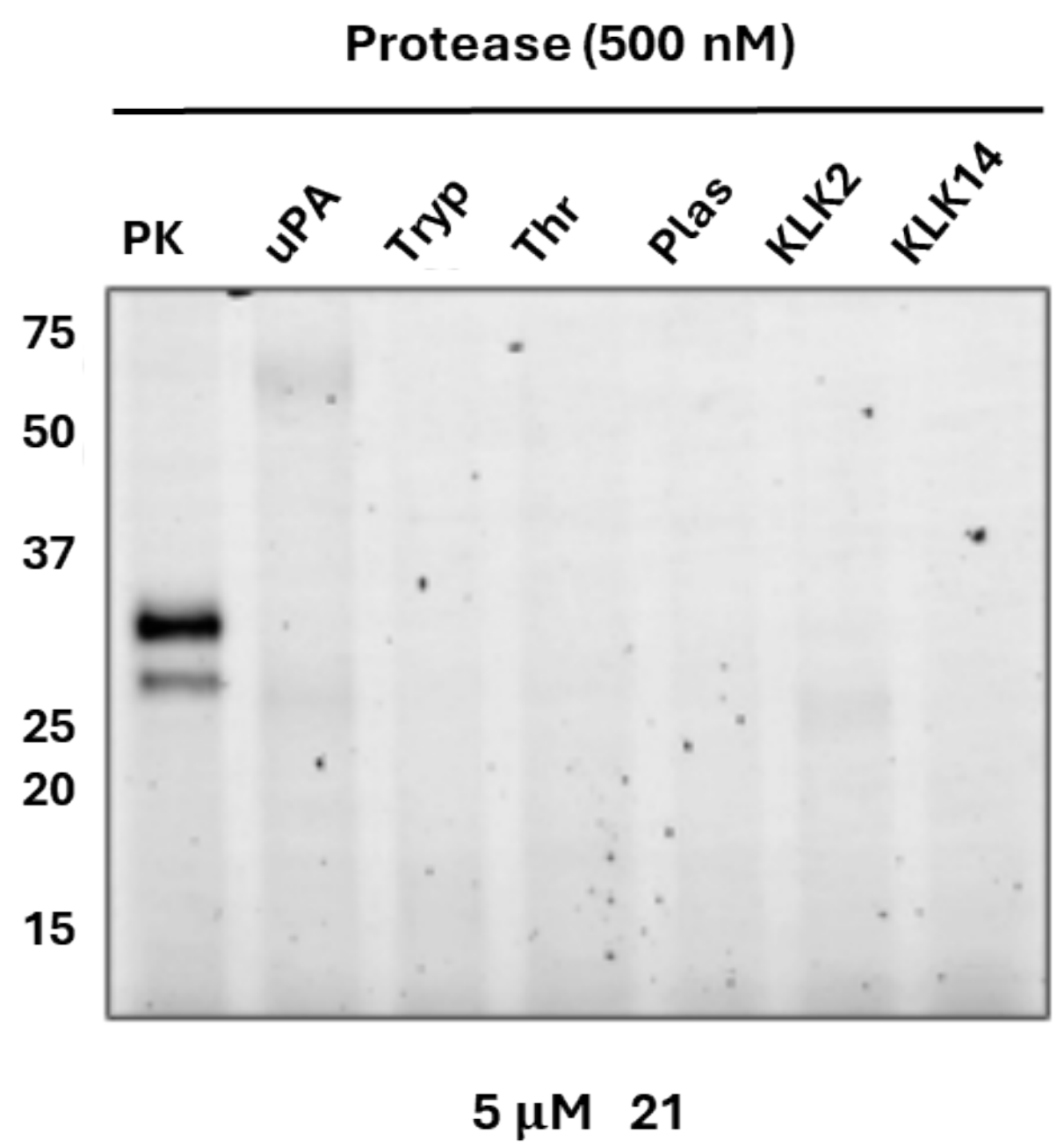

Supplementary Figure 8: In-gel Fluorescence profiling of 21 specificity across a serine protease panel. *Recombinant proteases (500 nM) were incubated with* ***21*** *(5 □M) in PBS (pH 7.4) for 16 h at 37 °C. Following SDS-PAGE resolution, covalent labelling was visualised via in-gel fluorescence following CuAAC conjugation to TAMRA-azide. Protease abbreviations: PK, plasma kallikrein; uPA, urokinase-type plasminogen activator; Tryp, trypsin; Thr, thrombin; Plas, plasmin; KLK2, kallikrein-2; KLK14, kallikrein-14. Molecular weight markers (in kDa) are indicated on the left.*

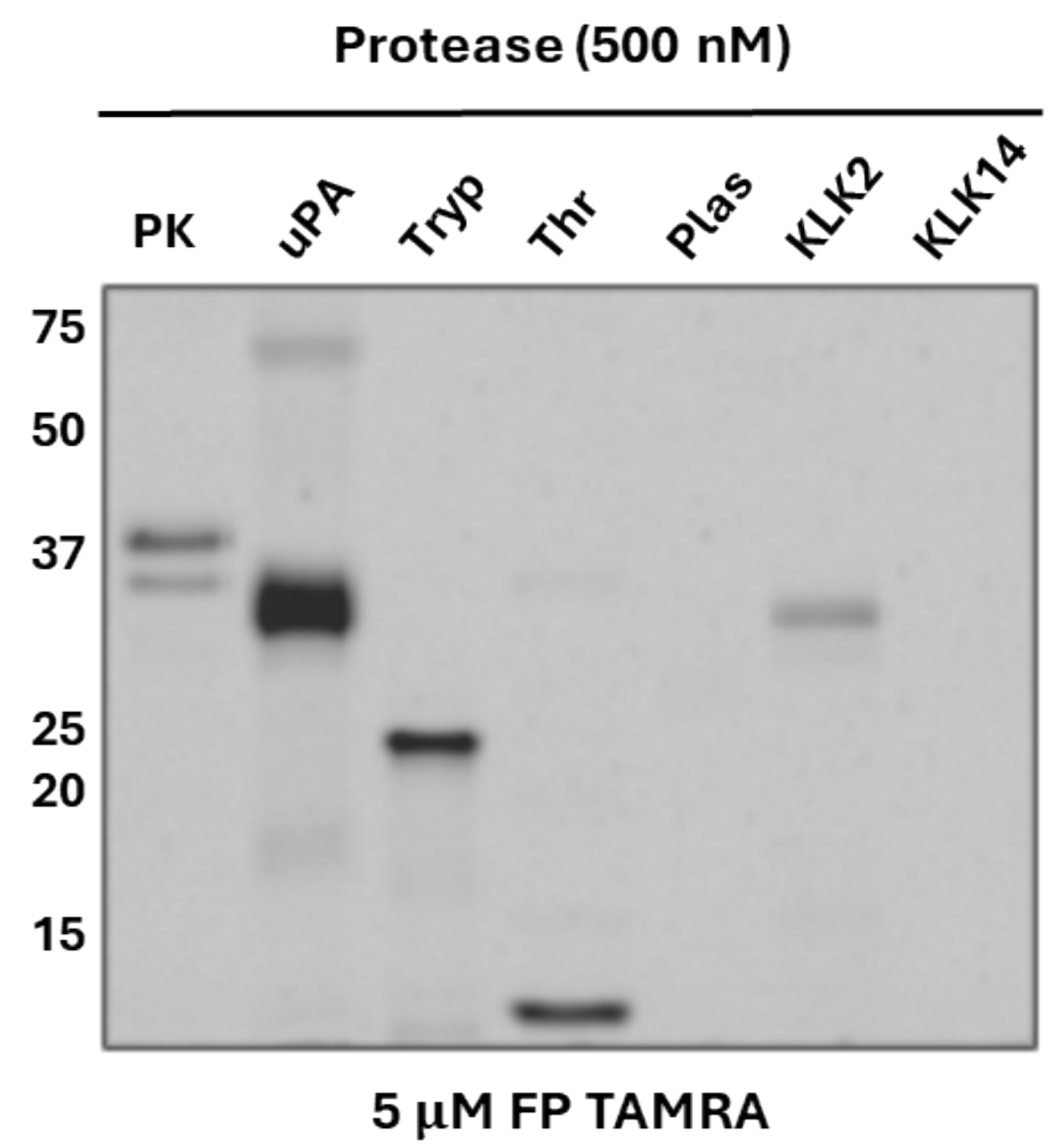

Supplementary Figure 9: In-gel fluorescence profiling of FP-TAMRA reactivity across a serine protease panel. *Recombinant proteases (500 nM) were incubated with the broad-spectrum activity-based probe FP-TAMRA (5 μM) in PBS (pH 7.4) for 60 min at 37 °C. Following SDS-PAGE resolution, covalent labelling was visualised via in-gel TAMRA fluorescence. Protease abbreviations: PK, plasma kallikrein; uPA, urokinase-type plasminogen activator; Tryp, trypsin; Thr, thrombin; Plas, plasmin; KLK2, kallikrein-2; KLK14, kallikrein-14. Molecular weight markers (in kDa) are indicated on the left.*

4.8 PC3 Cell Media Labelling with FP TAMRA

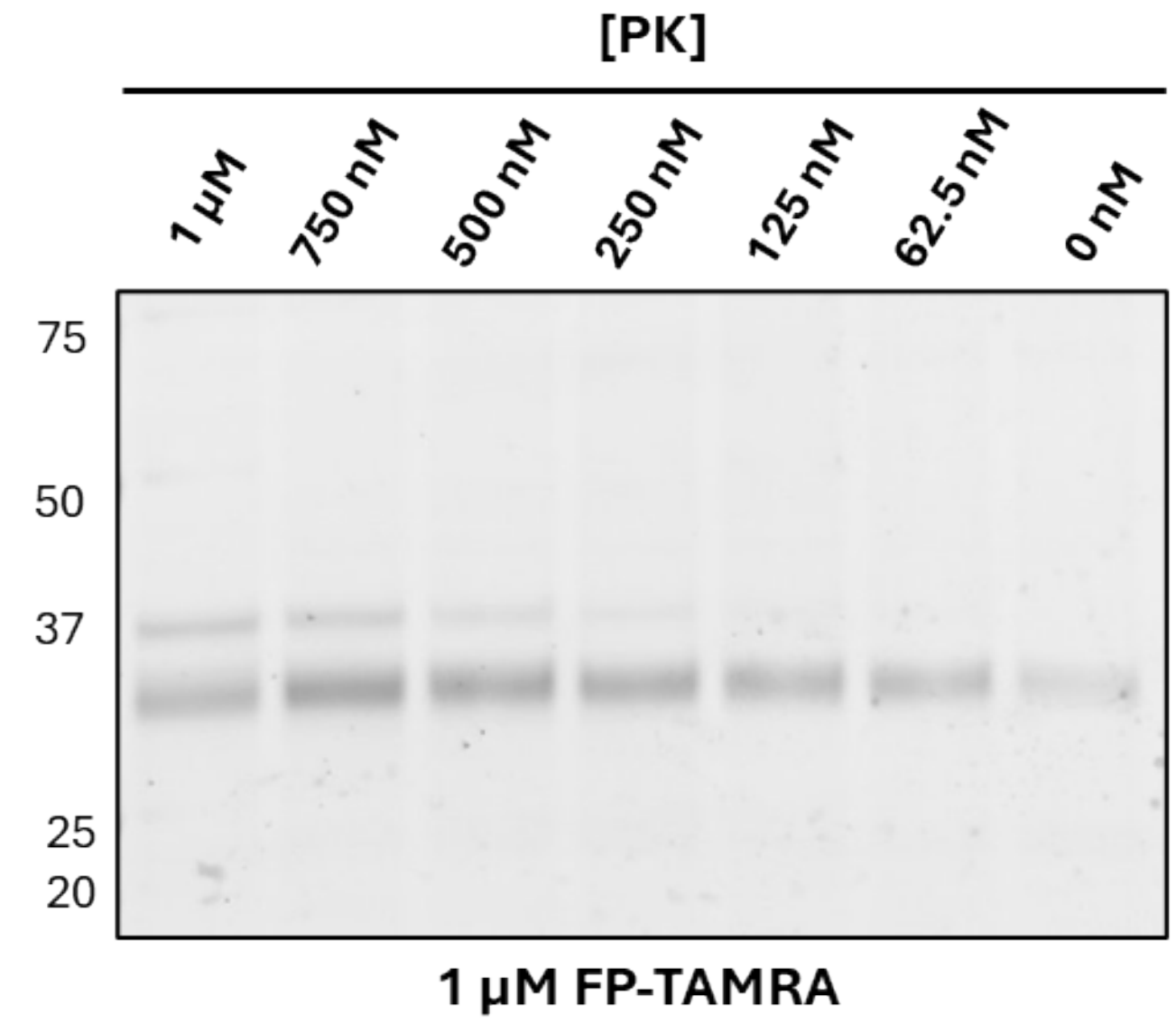

Supplementary Figure 10: Labelling of PC3 cell media with FP-TAMRA. *Conditioned Media (10 μg) was incubated with FP-TAMRA (5 μM) for 60 min at 37 °C. Following SDS-PAGE resolution, covalent labelling was visualised via in-gel TAMRA fluorescence.*

5 Probe Analytical Data

Compound 1

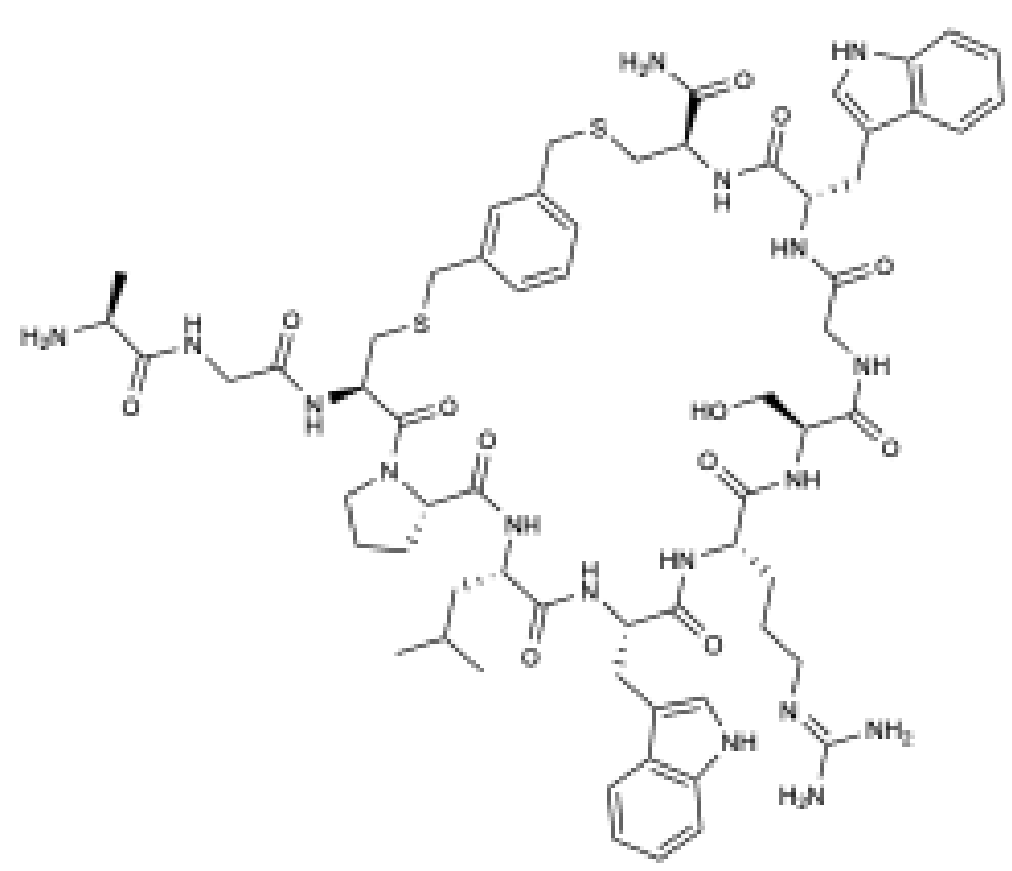

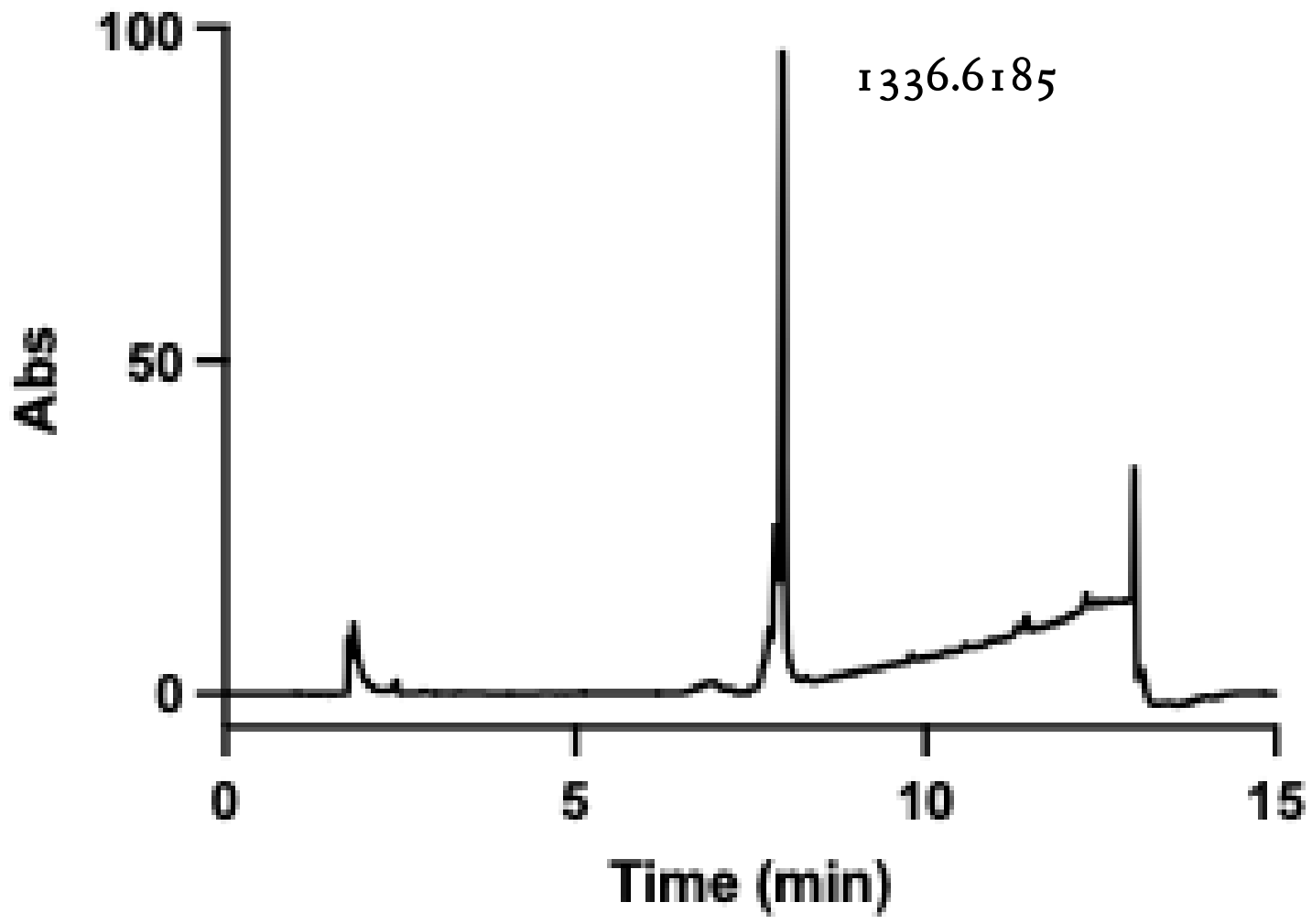

Chemical formula: $C_{63}H_{85}N_{17}O_{12}S_2^+$

Calculated $[M + H]^+ = 1336.60$

Observed $[M + H]^+ = 1336.6185$

Compound 2

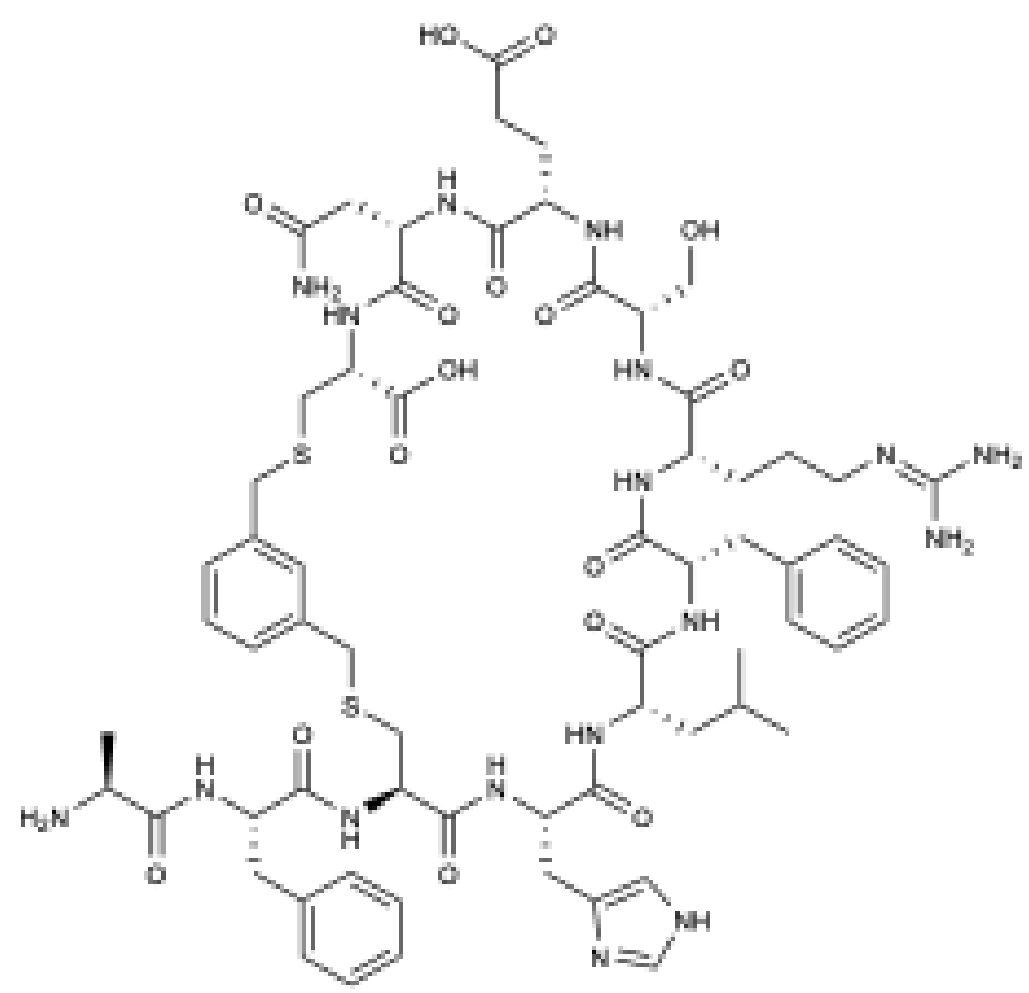

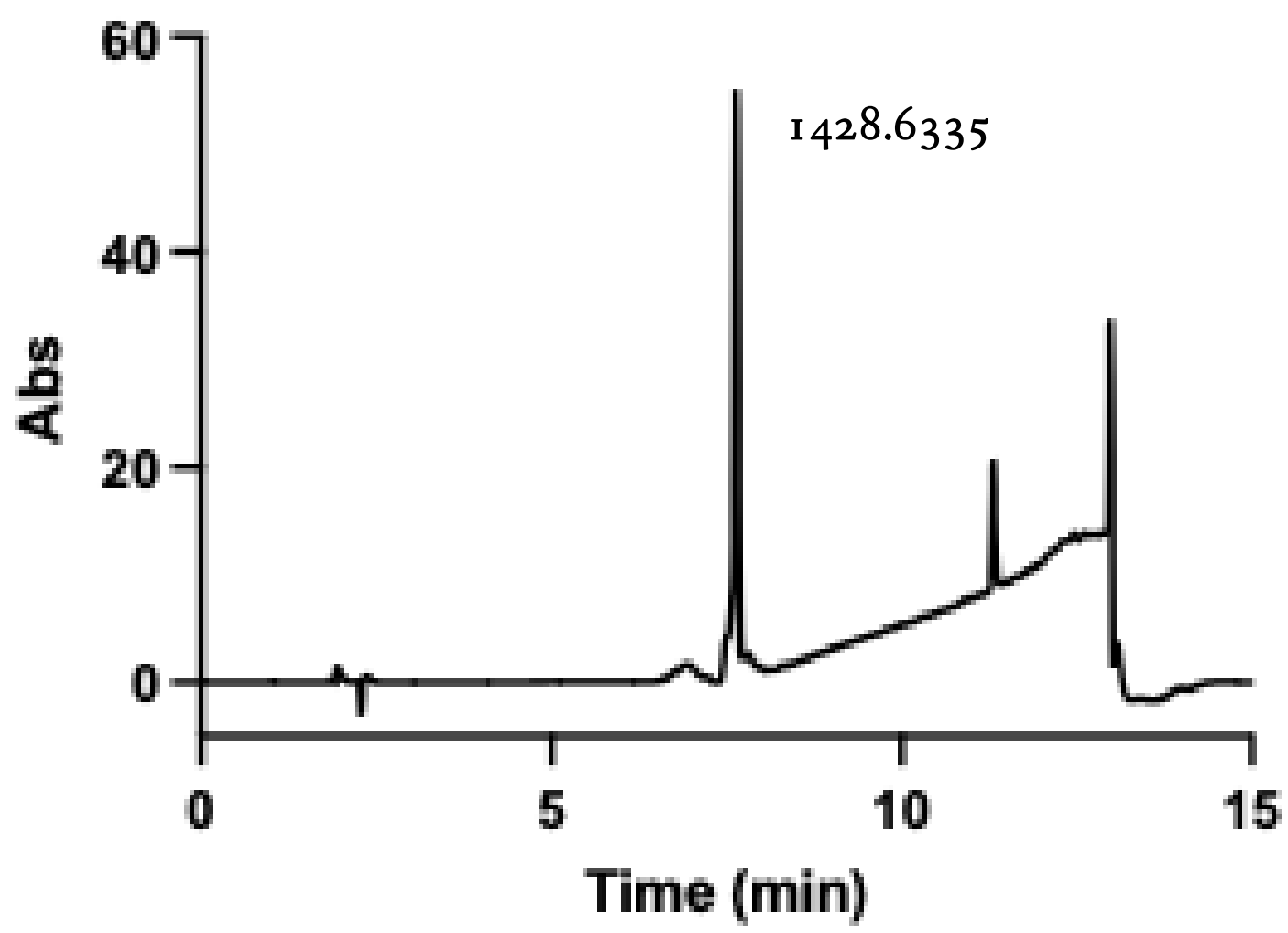

Chemical formula: $C_{65}H_{89}N_{17}O_{16}S_2^+$

Calculated $[M + H]^+ = 1428.61$

Observed $[M + H]^+ = 1428.6335$

Compound 3

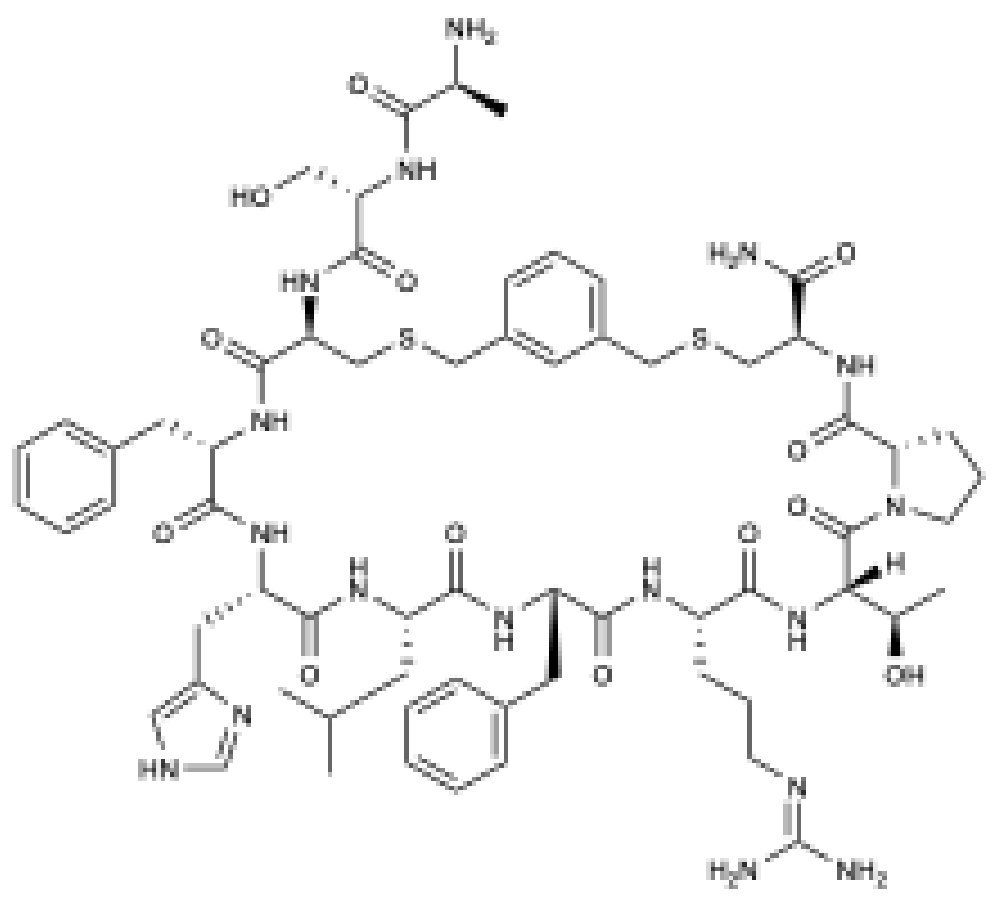

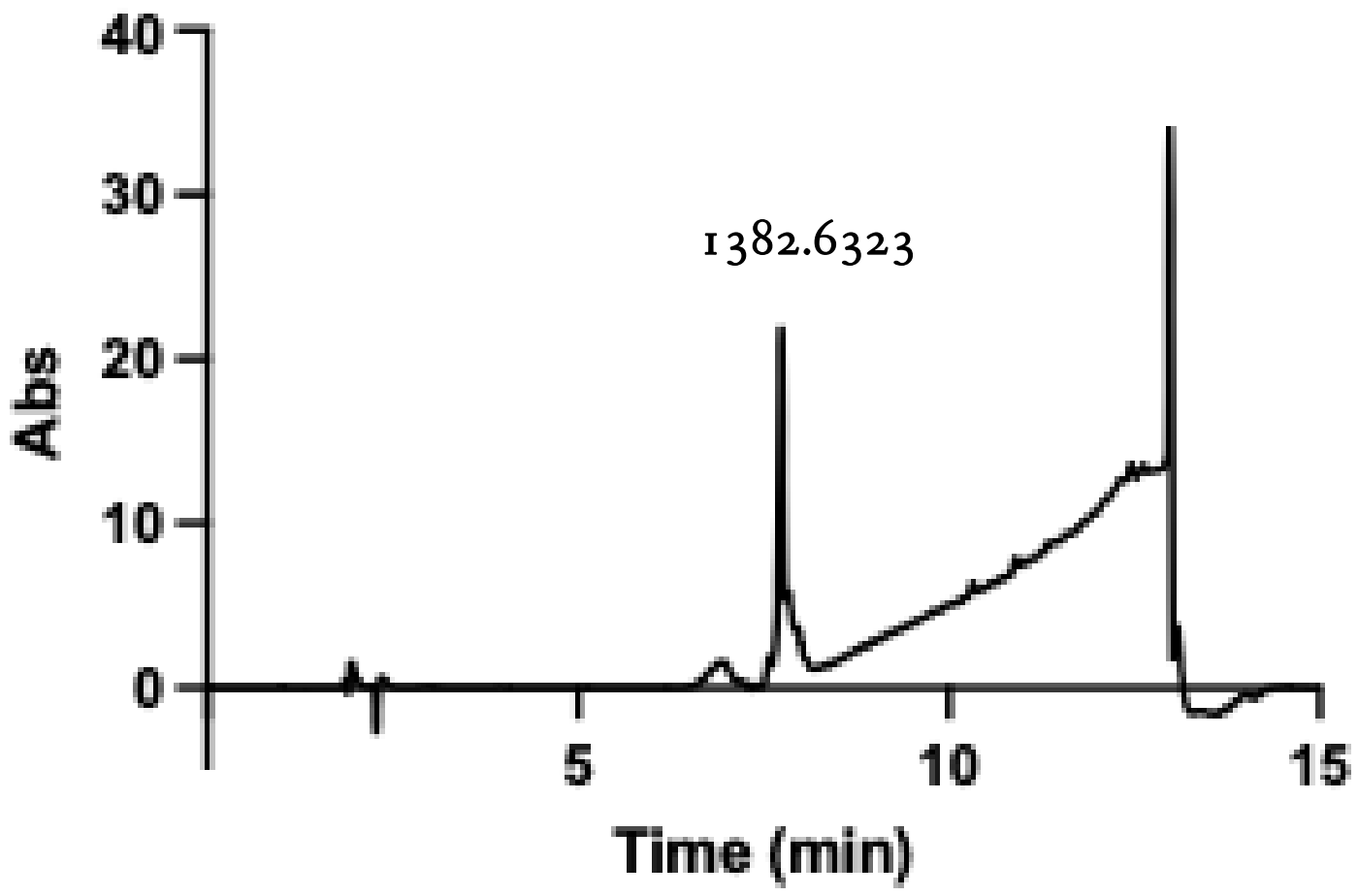

Chemical formula: $C_{65}H_{91}N_{17}O_{13}S_2^+$

Calculated $[M + H]^+$ = 1382.64

Observed $[M + H]^+$ = 1382.6323

Compound 4

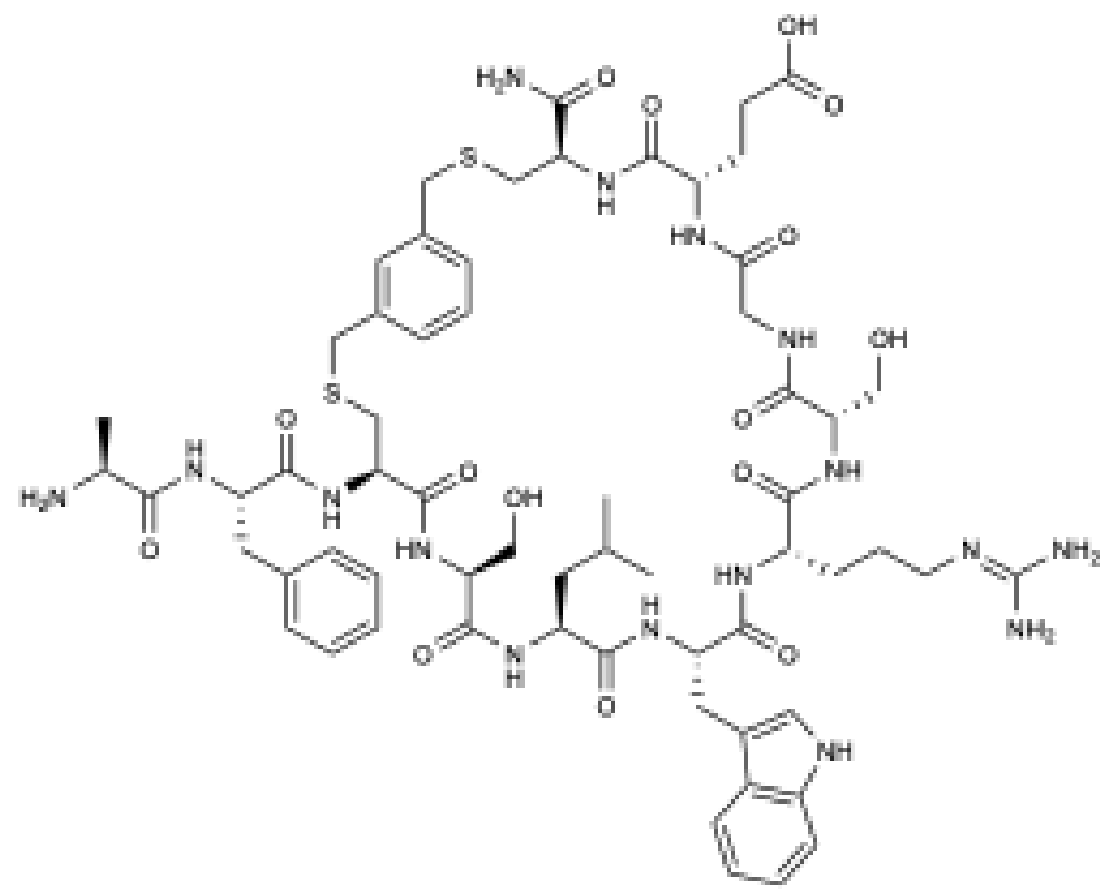

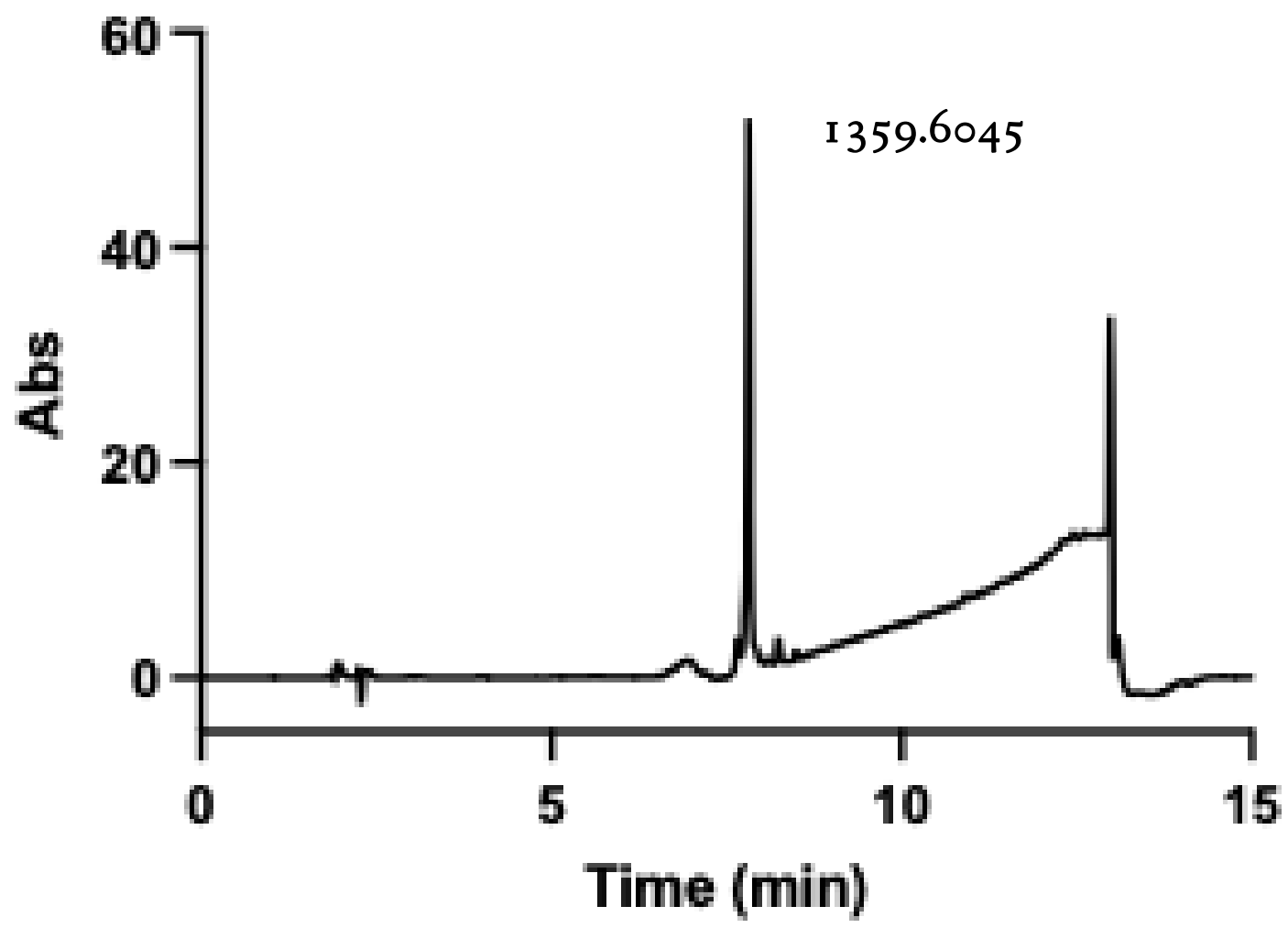

Chemical formula: $C_{62}H_{85}N_{17}O_{12}S_2^+$

Calculated $[M + H]^+$ = 1359.59

Observed $[M + H]^+$ = 1359.6045

Compound 5

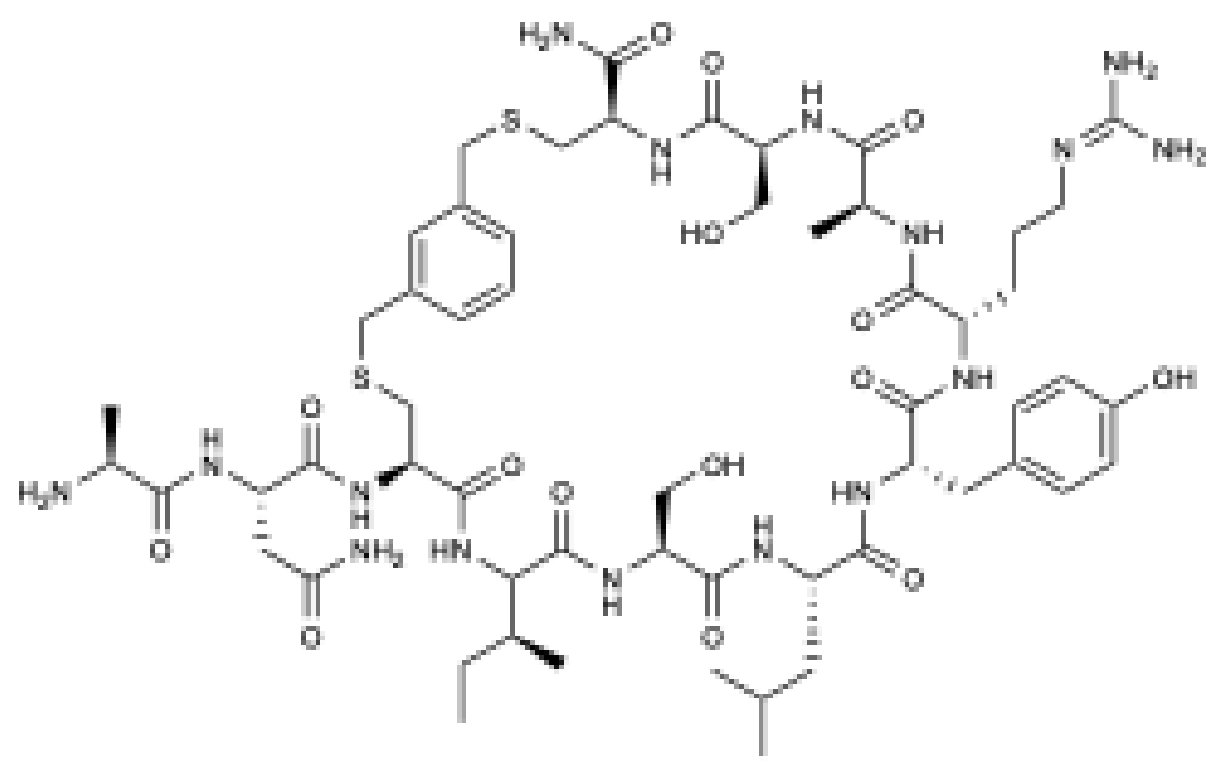

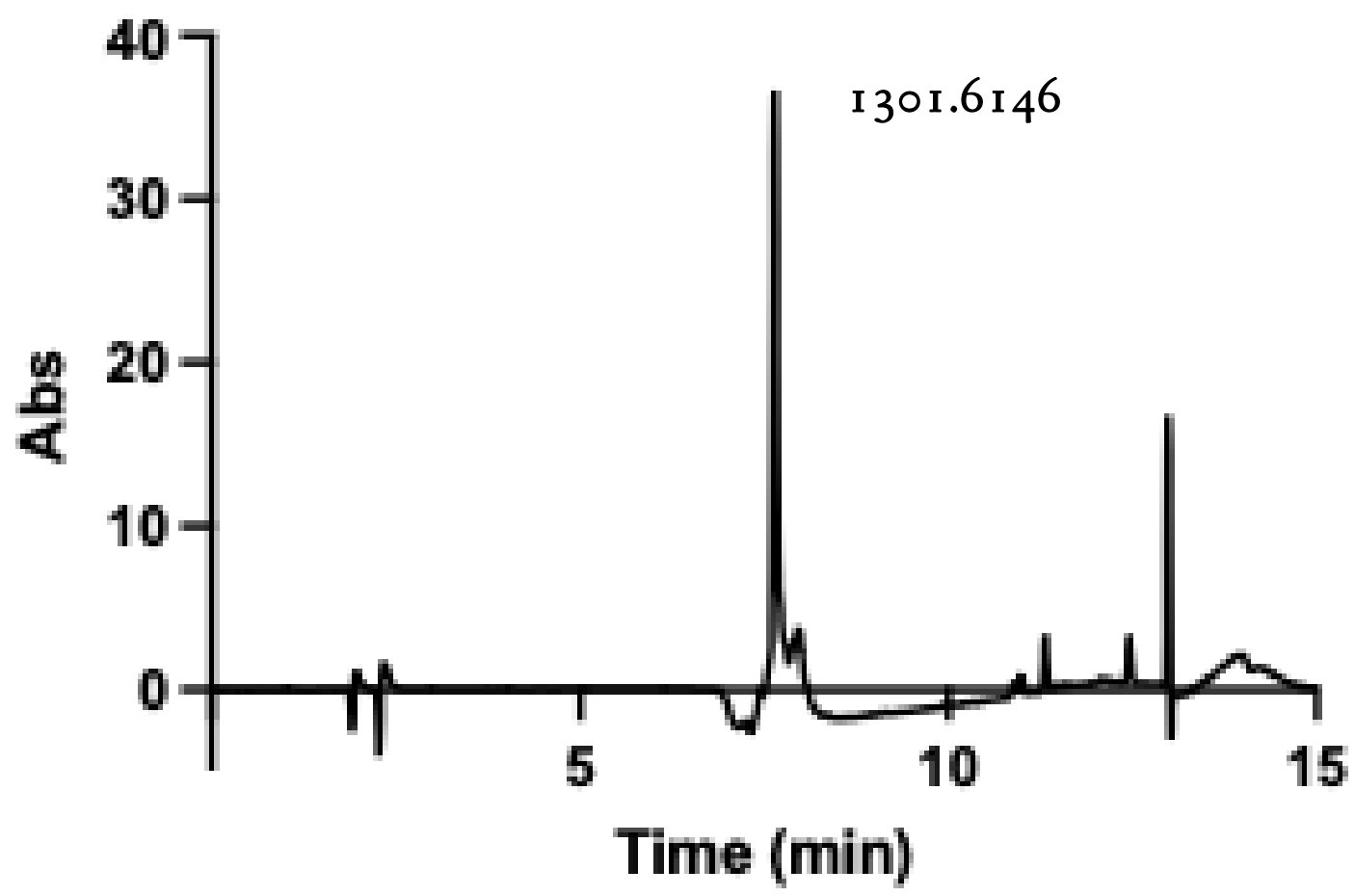

Chemical formula: $C_{57}H_{88}N_{16}O_{15}S_2^+$

Calculated $[M + H]^+$ = 1301.55

Observed $[M + H]^+$ = 1301.6146

Compound 6

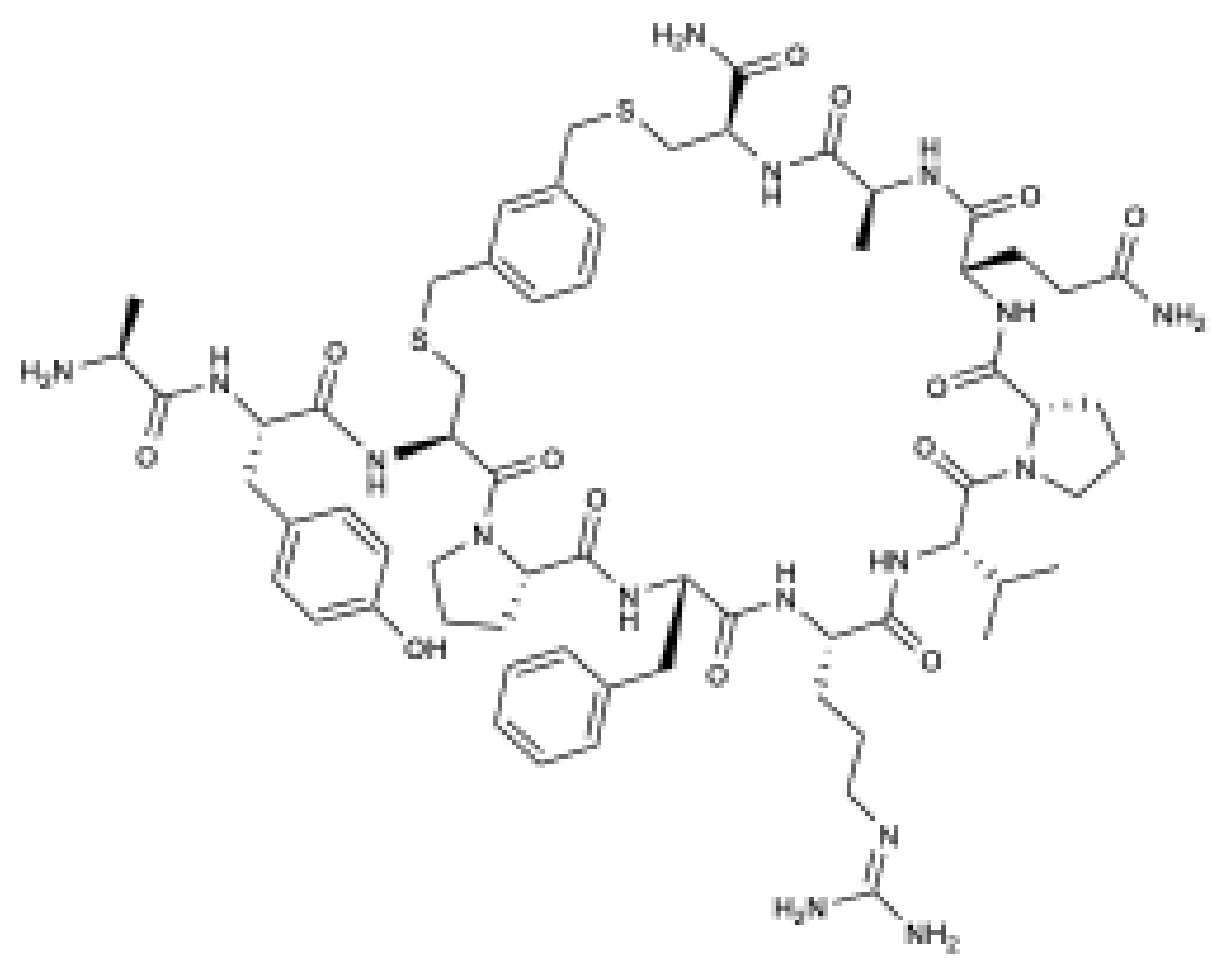

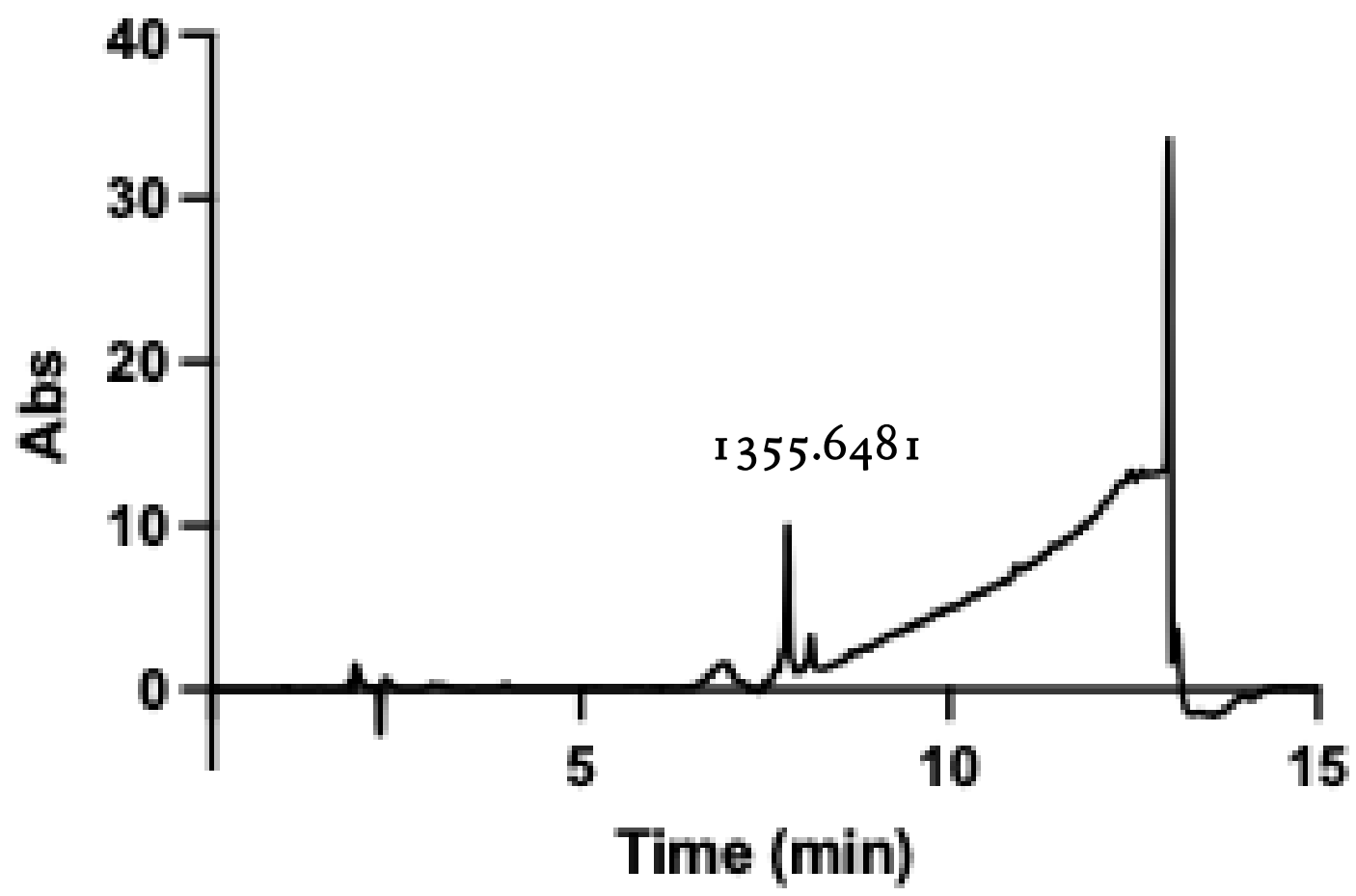

Chemical formula: $C_{64}H_{90}N_{16}O_{13}S_2^+$

Calculated $[M + H]^+ = 1355.64$

Observed $[M + H]^+ = 1355.6481$

Compound 7

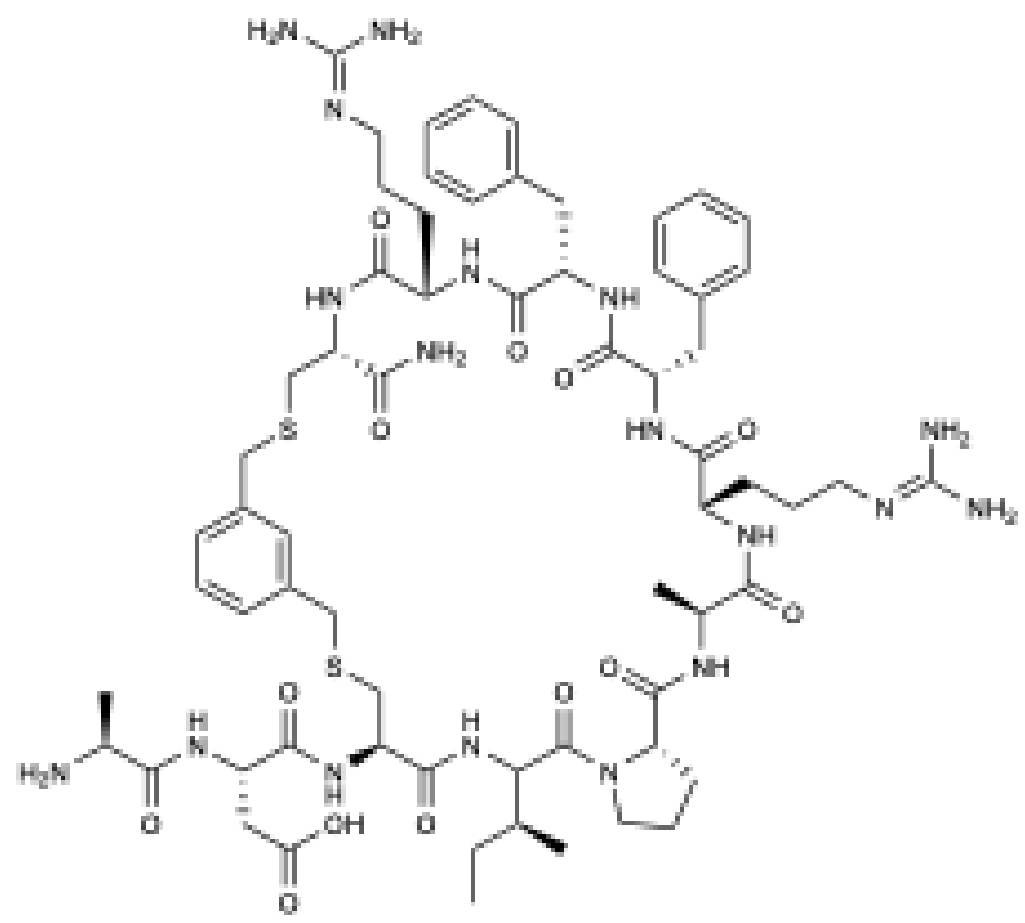

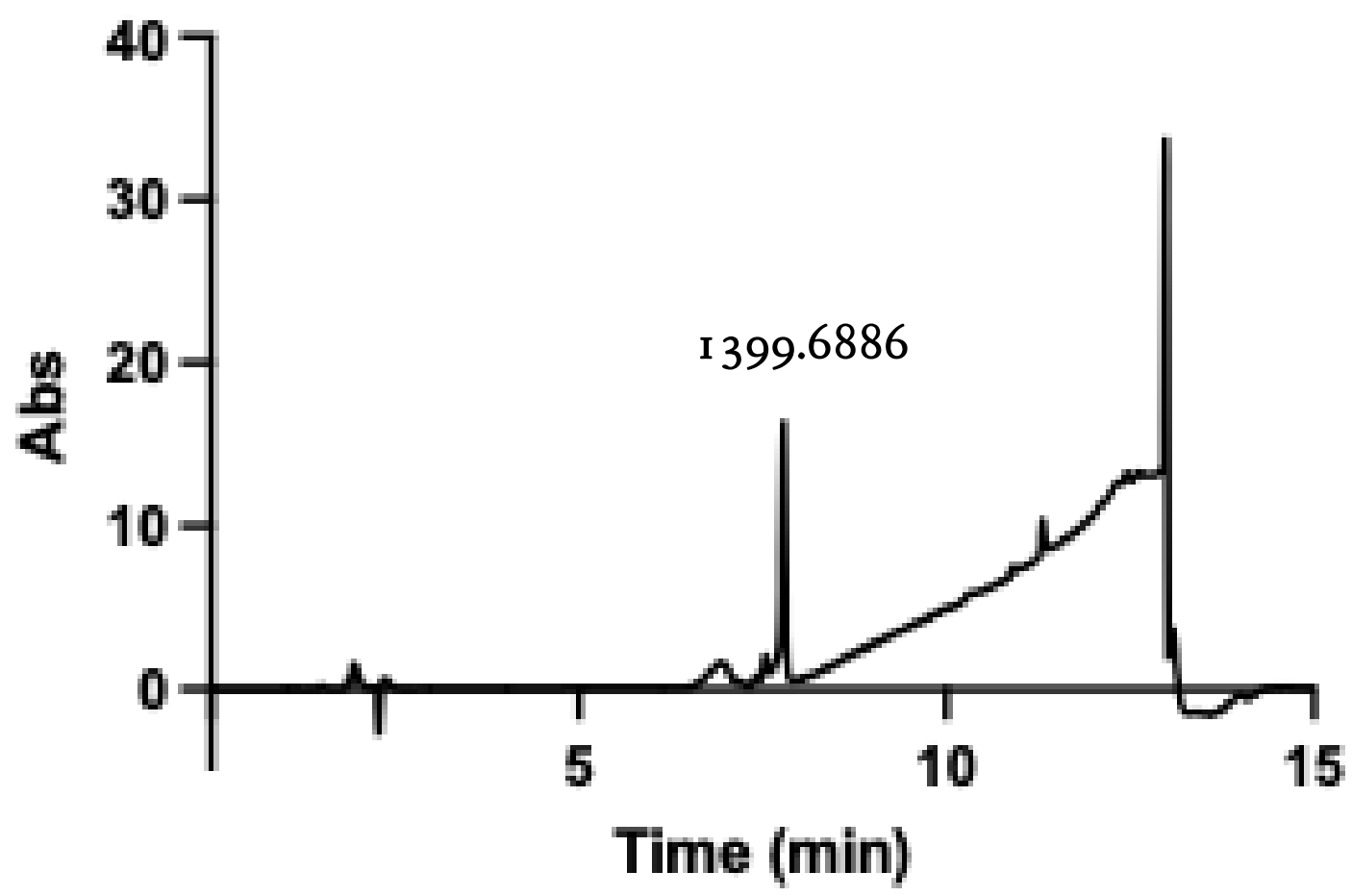

Chemical formula: $C_{65}H_{94}N_{18}O_{13}S_2^+$

Calculated $[M + H]^+$ = 1399.70

Observed $[M + H]^+$ = 1399.6886

Compound 8

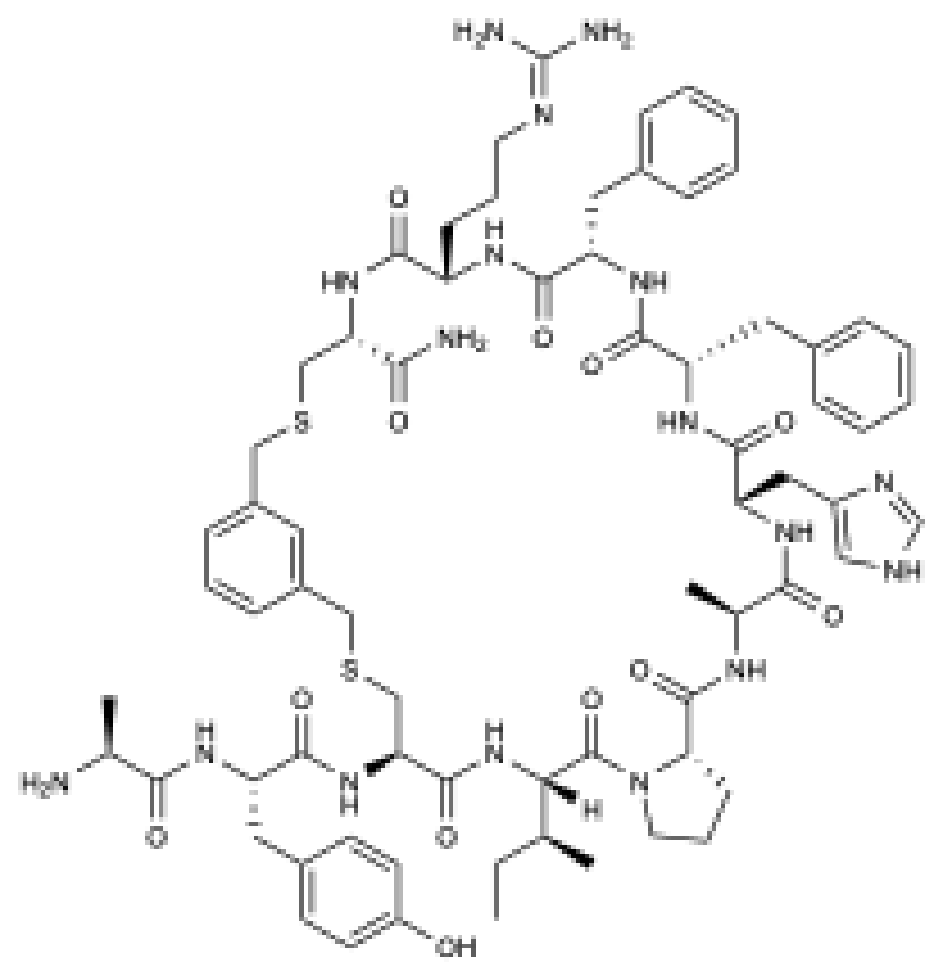

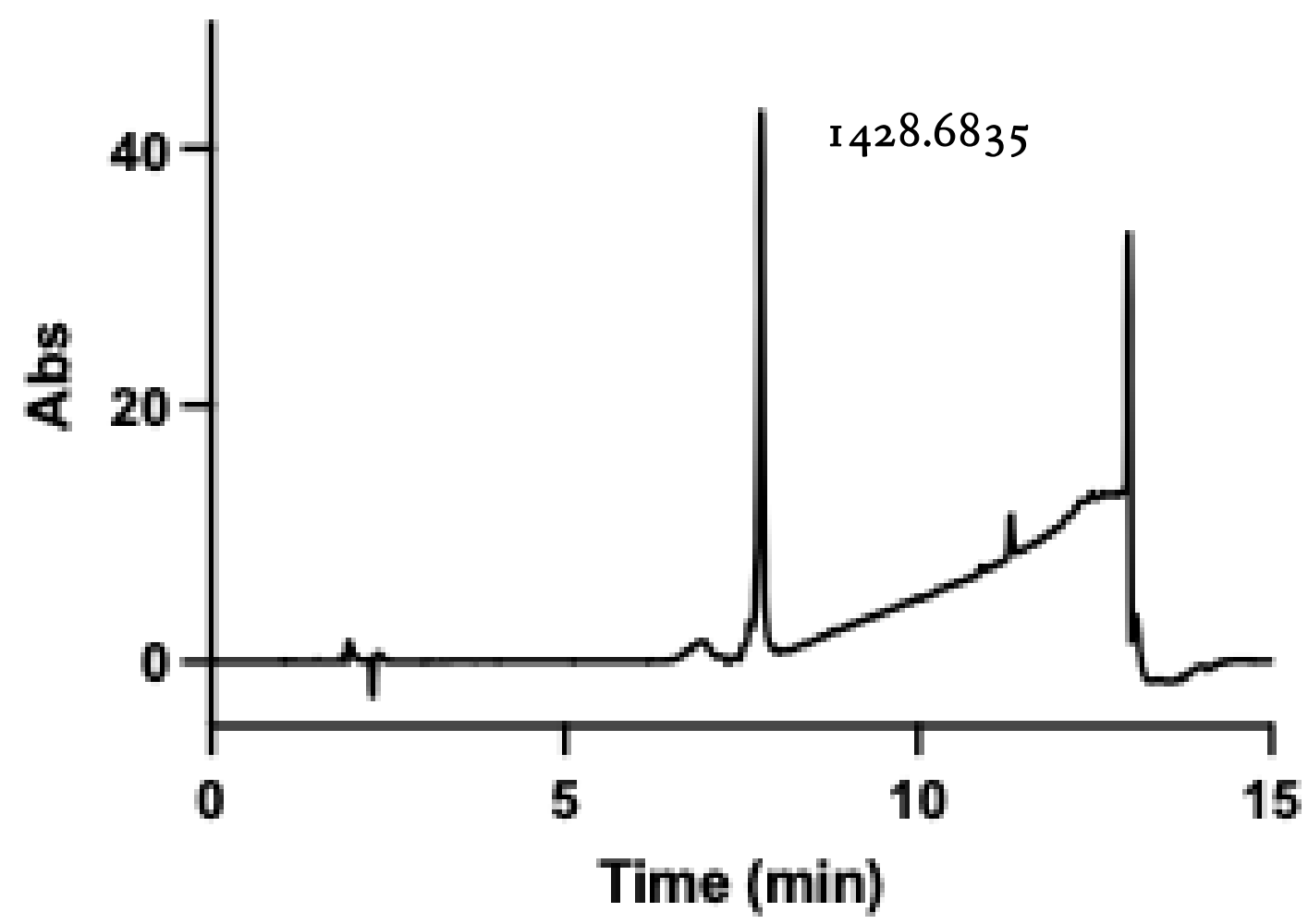

Chemical formula: $C_{70}H_{93}N_{17}O_{12}S_2^+$

Calculated $[M + H]^+$ = 1428.74

Observed $[M + H]^+$ = 1428.6835

Compound 9

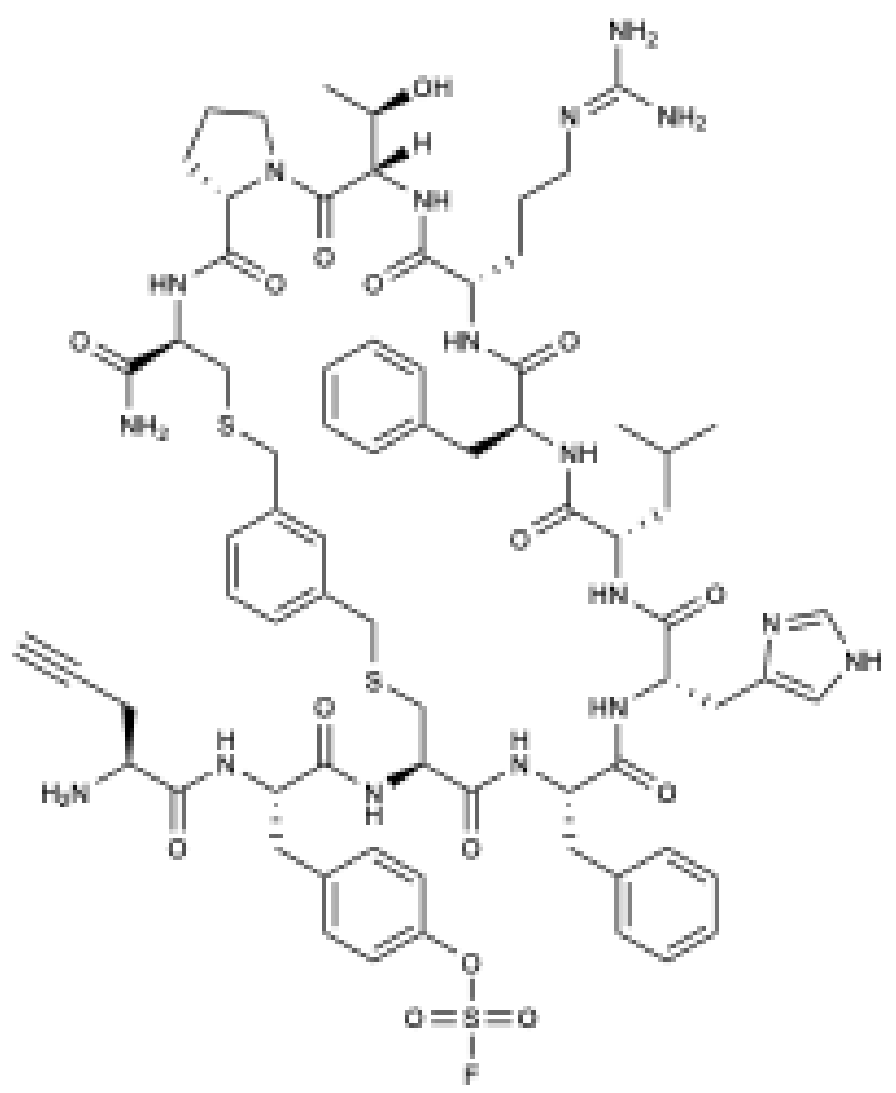

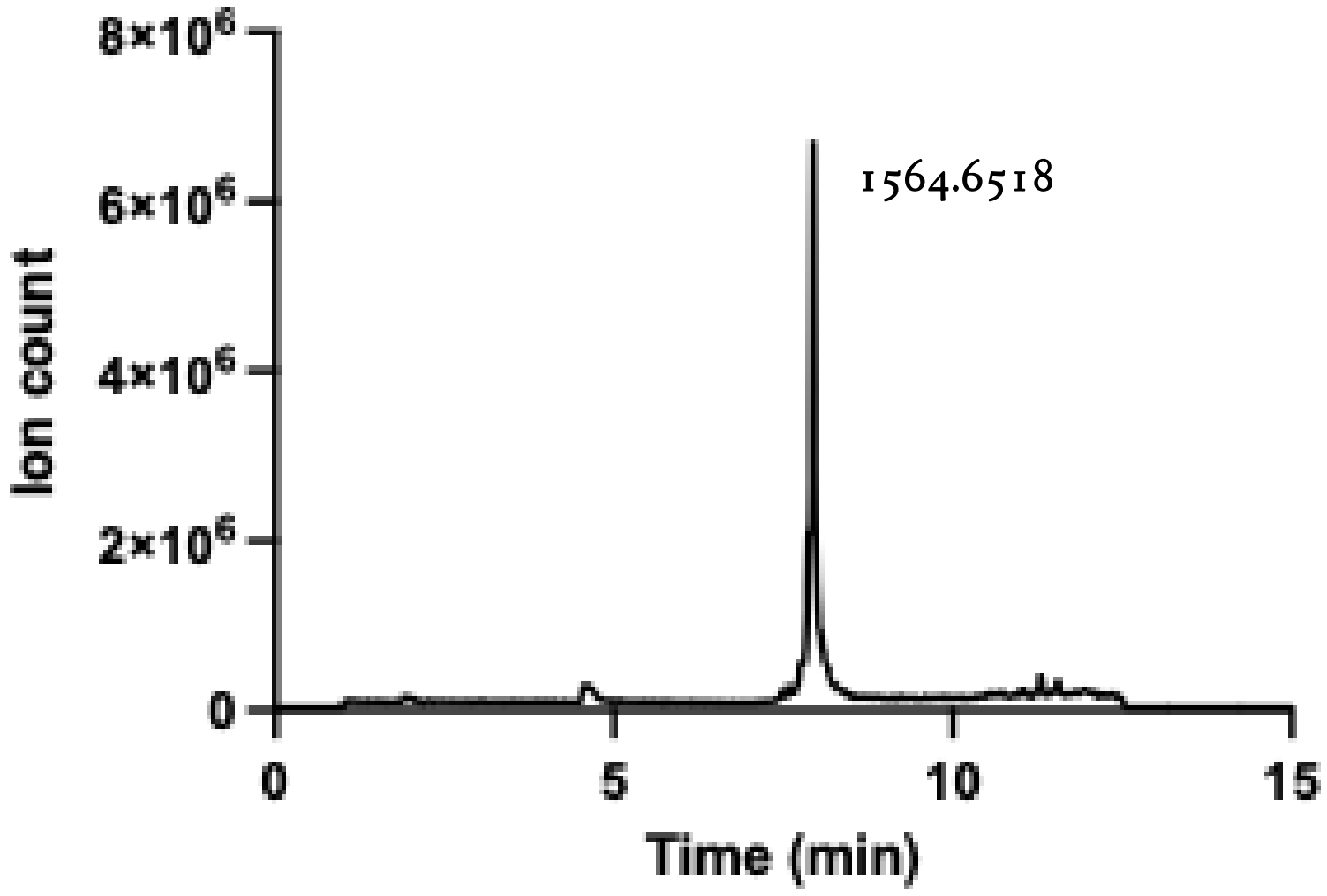

Chemical formula: $C_{73}H_{94}FN_{17}O_{15}S_3^+$

Calculated $[M + H]^+ = 1564.84$

Observed $[M + H]^+ = 1564.6518$

Compound 10

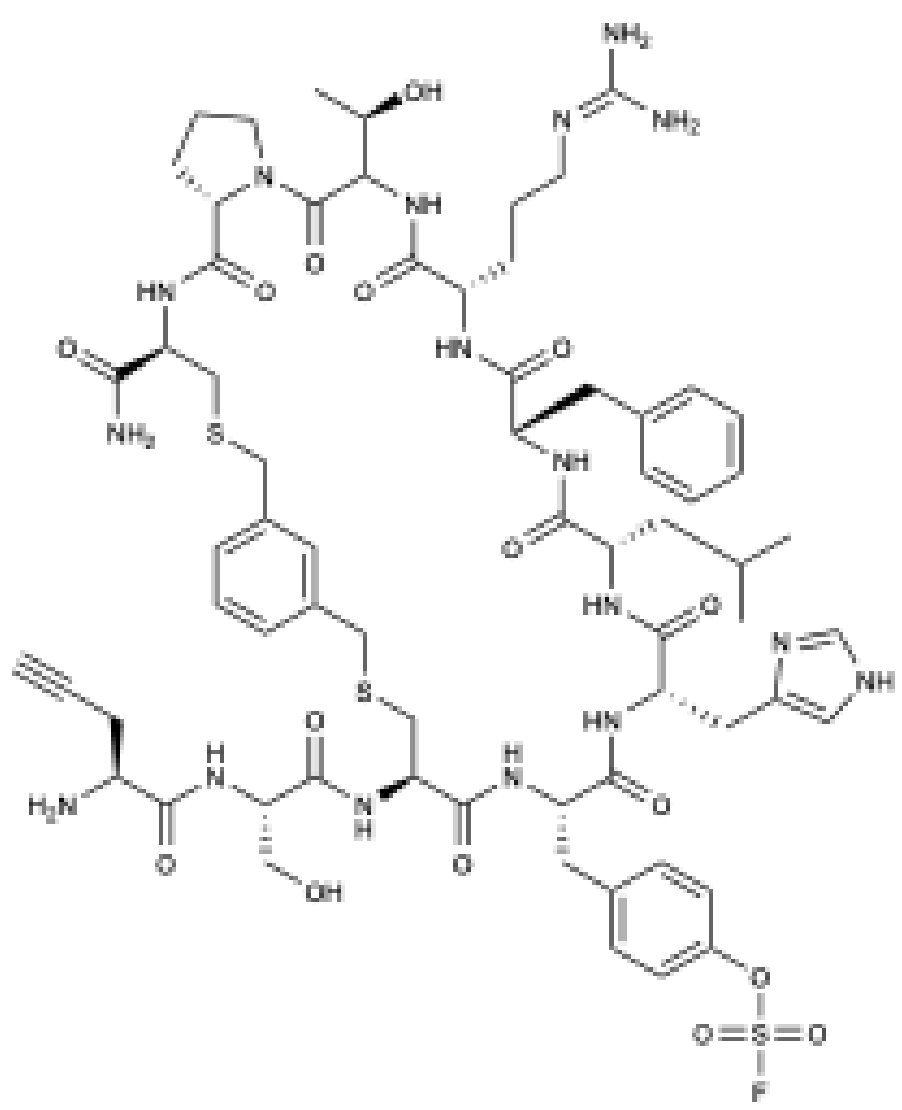

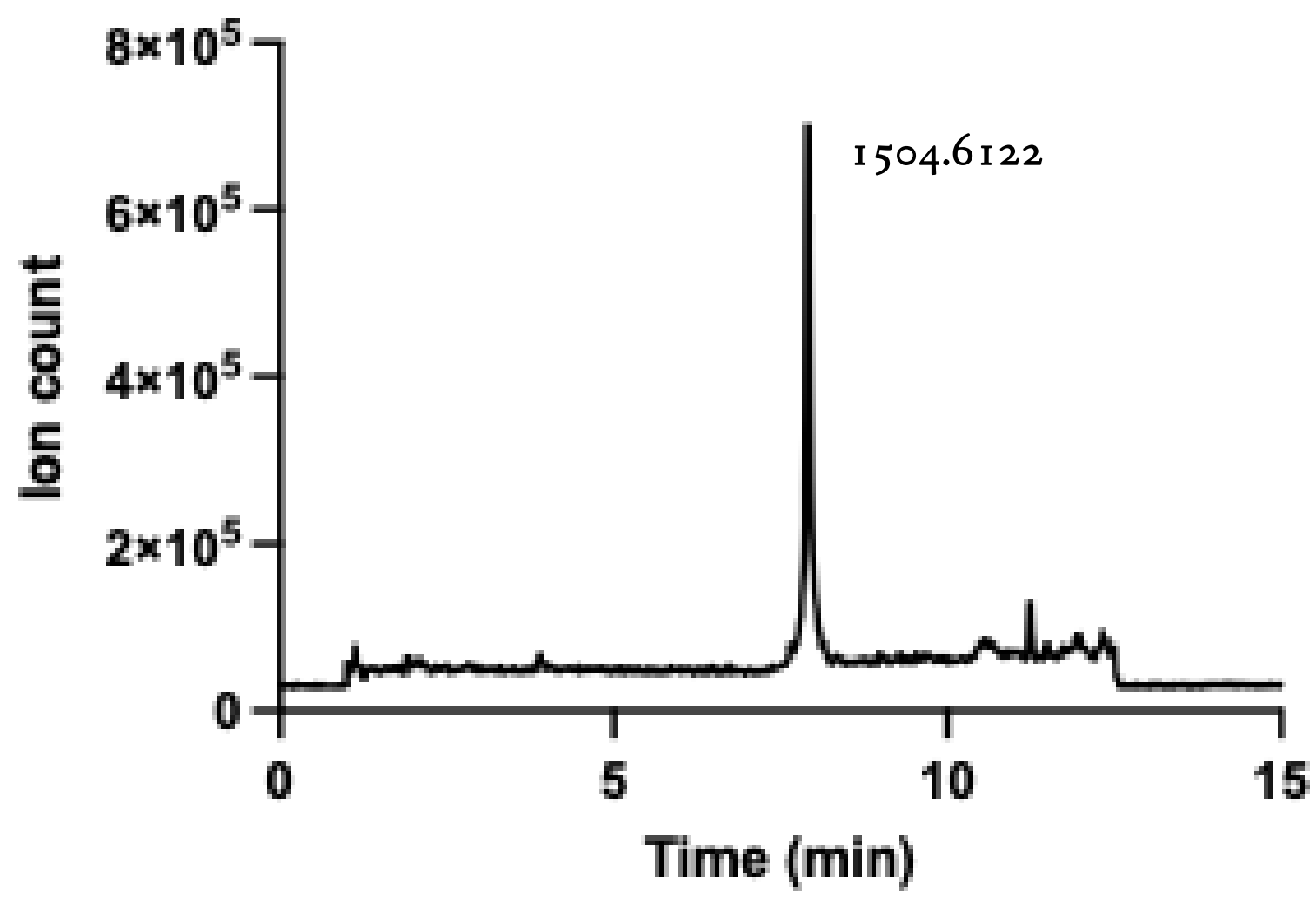

Chemical formula: $C_{67}H_{90}FN_{17}O_{16}S_3^+$

Calculated $[M + H]^+ = 1504.74$

Observed $[M + H]^+ = 1504.6122$

Compound 11

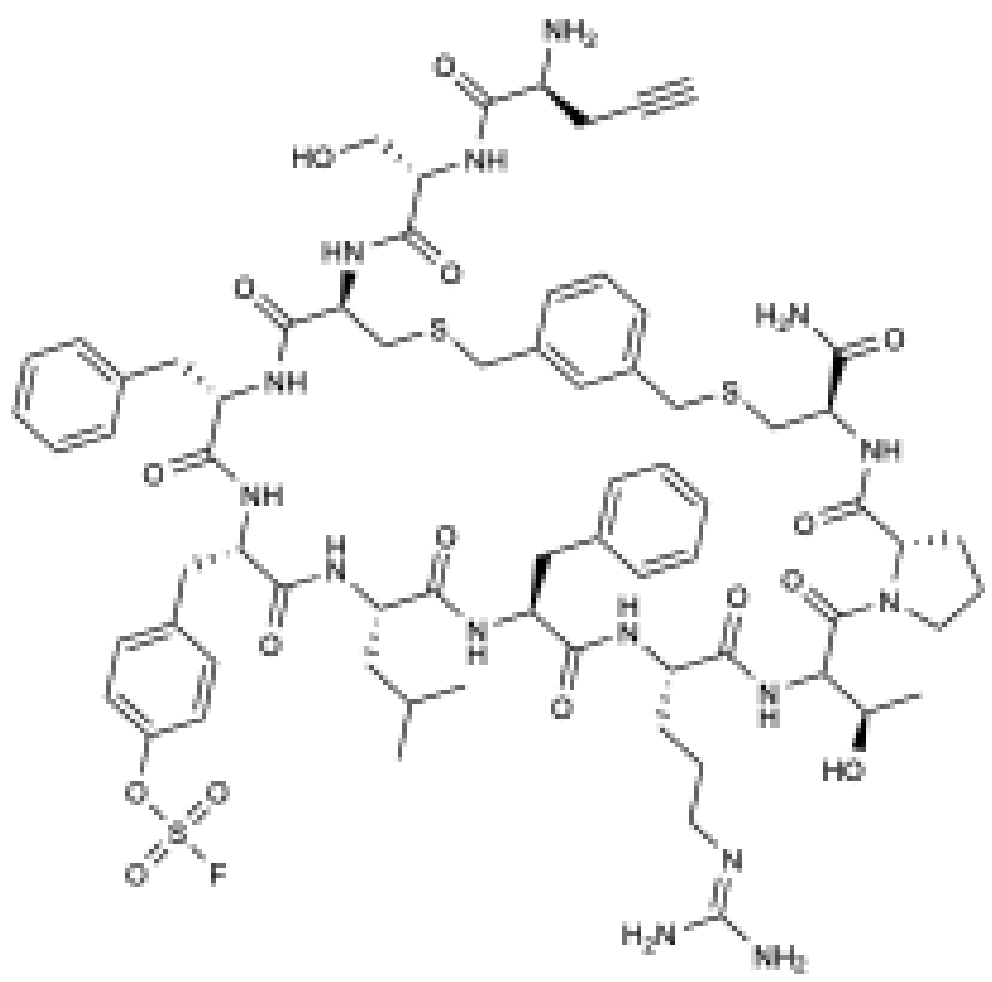

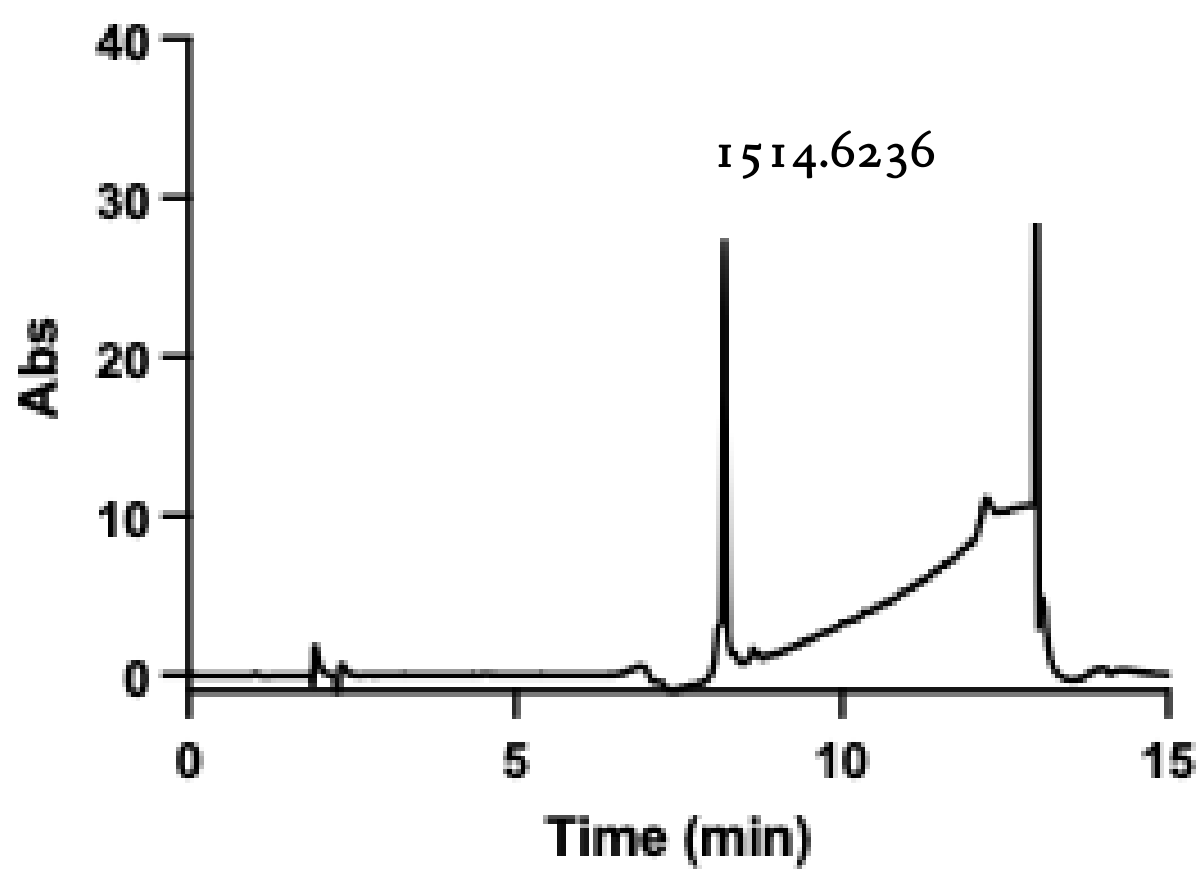

Chemical formula: $C_{70}H_{92}FN_{15}O_{16}S_3^+$

Calculated $[M + H]^+$ = 1514.77

Observed $[M + H]^+$ = 1514.6236

Compound 12

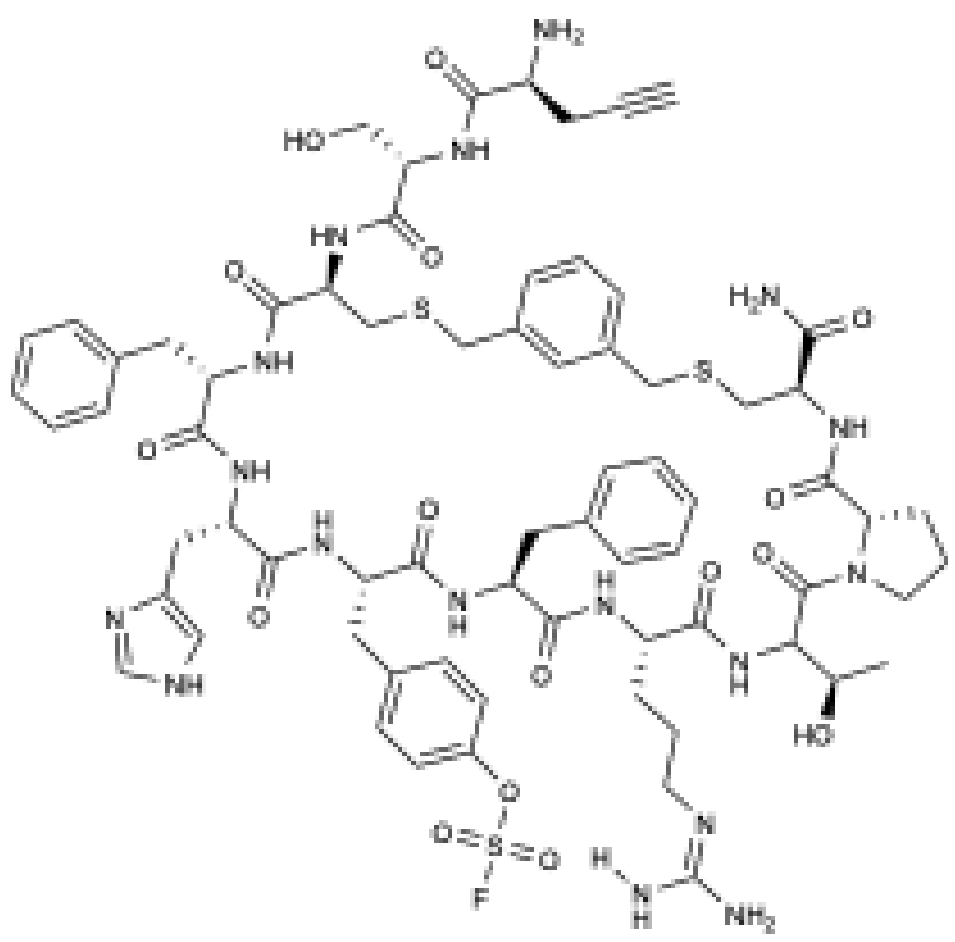

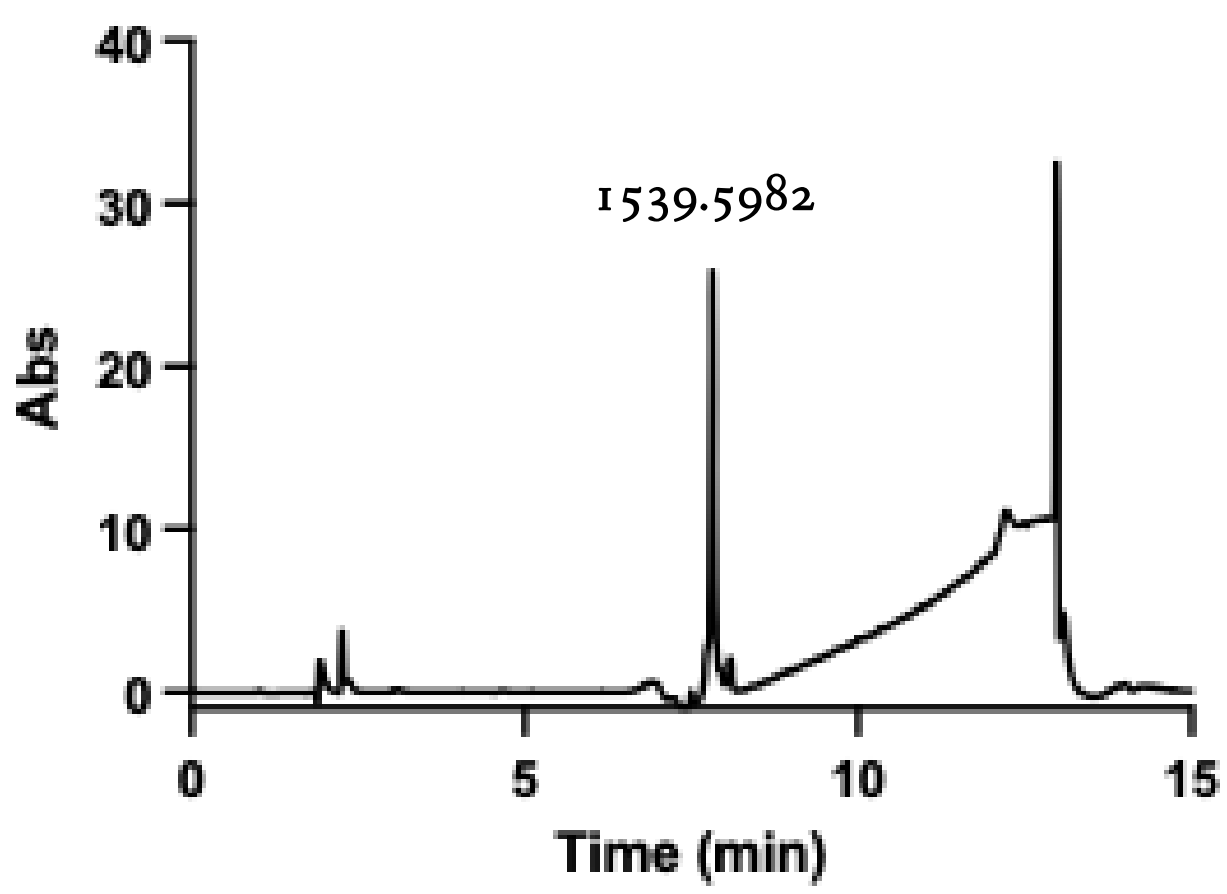

Chemical formula: $C_{70}H_{88}FN_{17}O_{16}S_3^+$

Calculated $[M + H]^+ = 1539.76$

Observed $[M + H]^+ = 1539.5982$

Compound 13

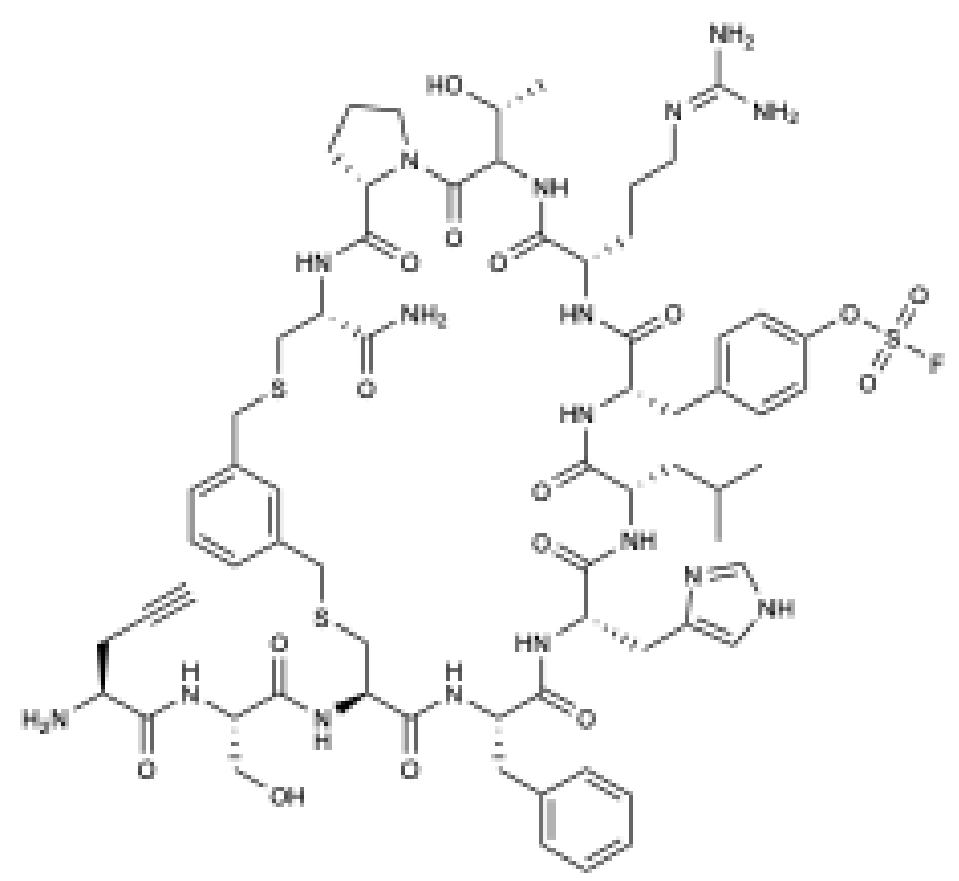

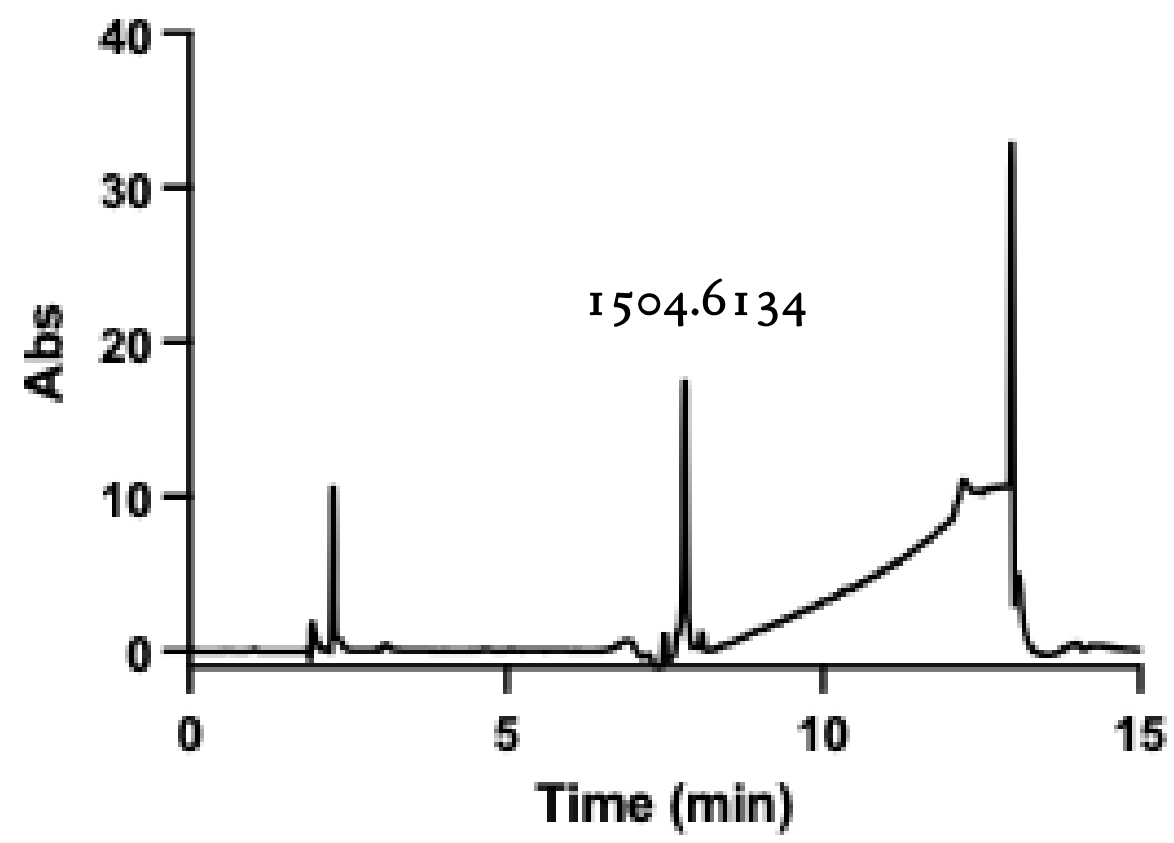

Chemical formula: $C_{67}H_{90}FN_{17}O_{16}S_3^+$

Calculated $[M + H]^+$ = 1504.74

Observed $[M + H]^+$ = 1504.6134

Compound 14

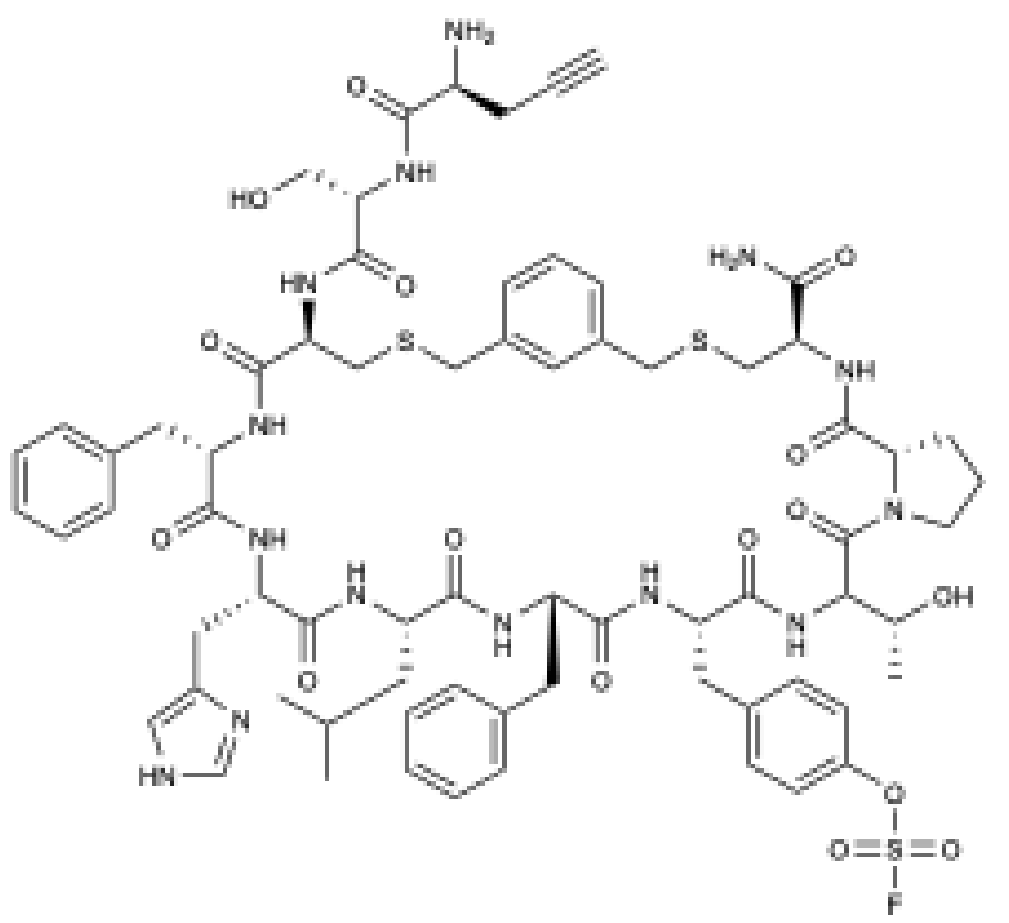

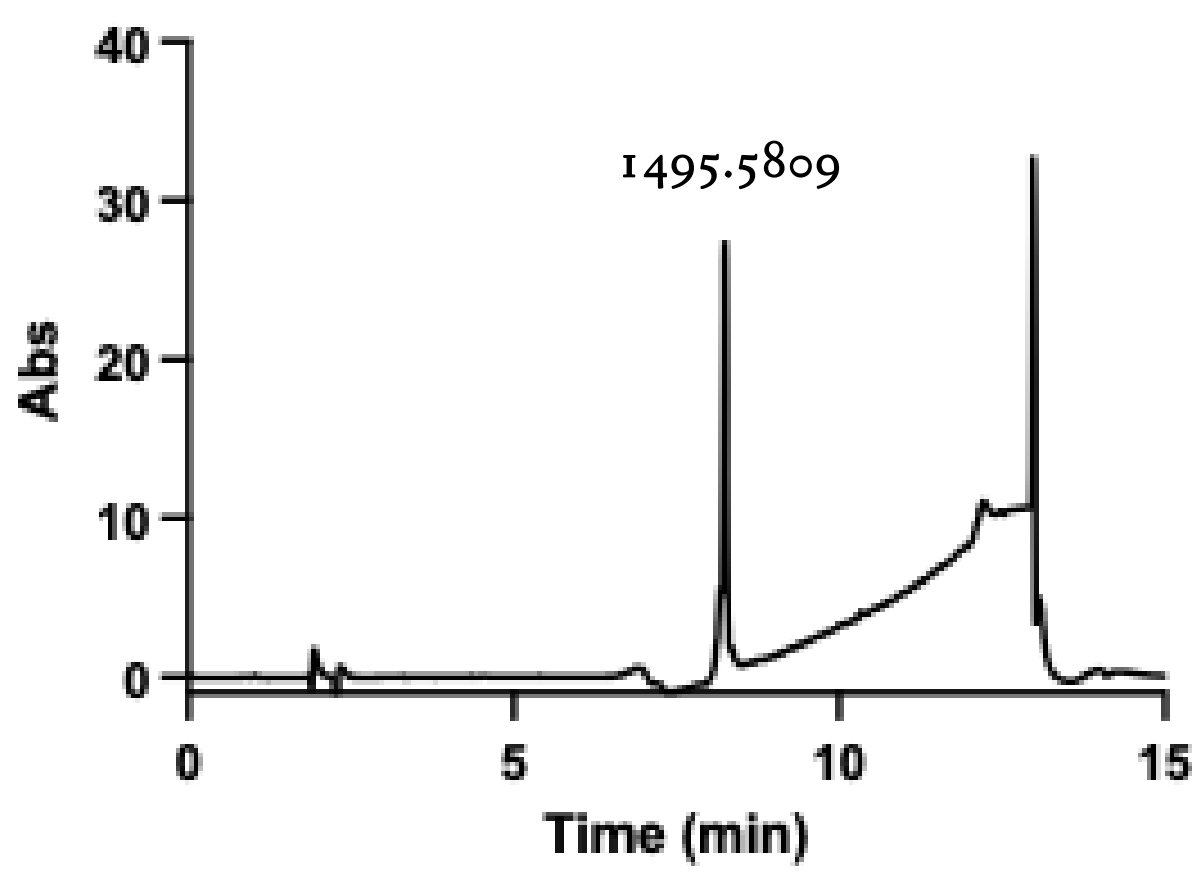

Chemical formula: $C_{70}H_{87}FN_{14}O_{16}S_3^+$

Calculated $[M + H]^+$ = 1495.73

Observed $[M + H]^+$ = 1495.5809

Compound 15

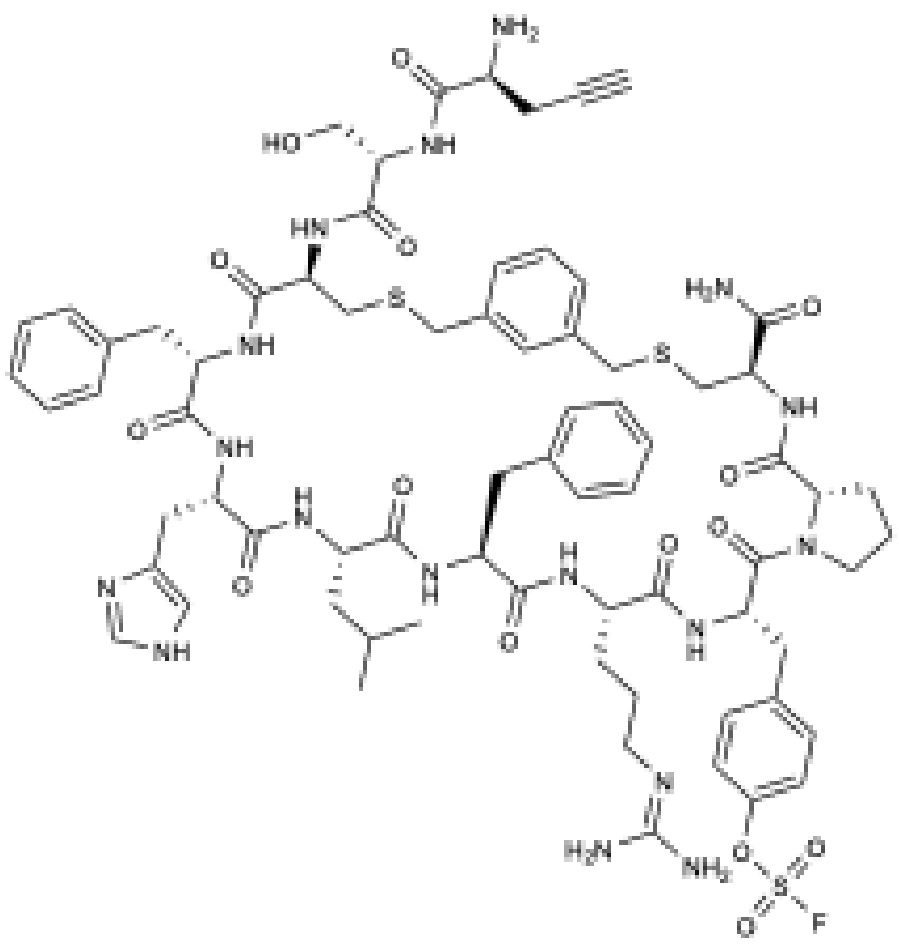

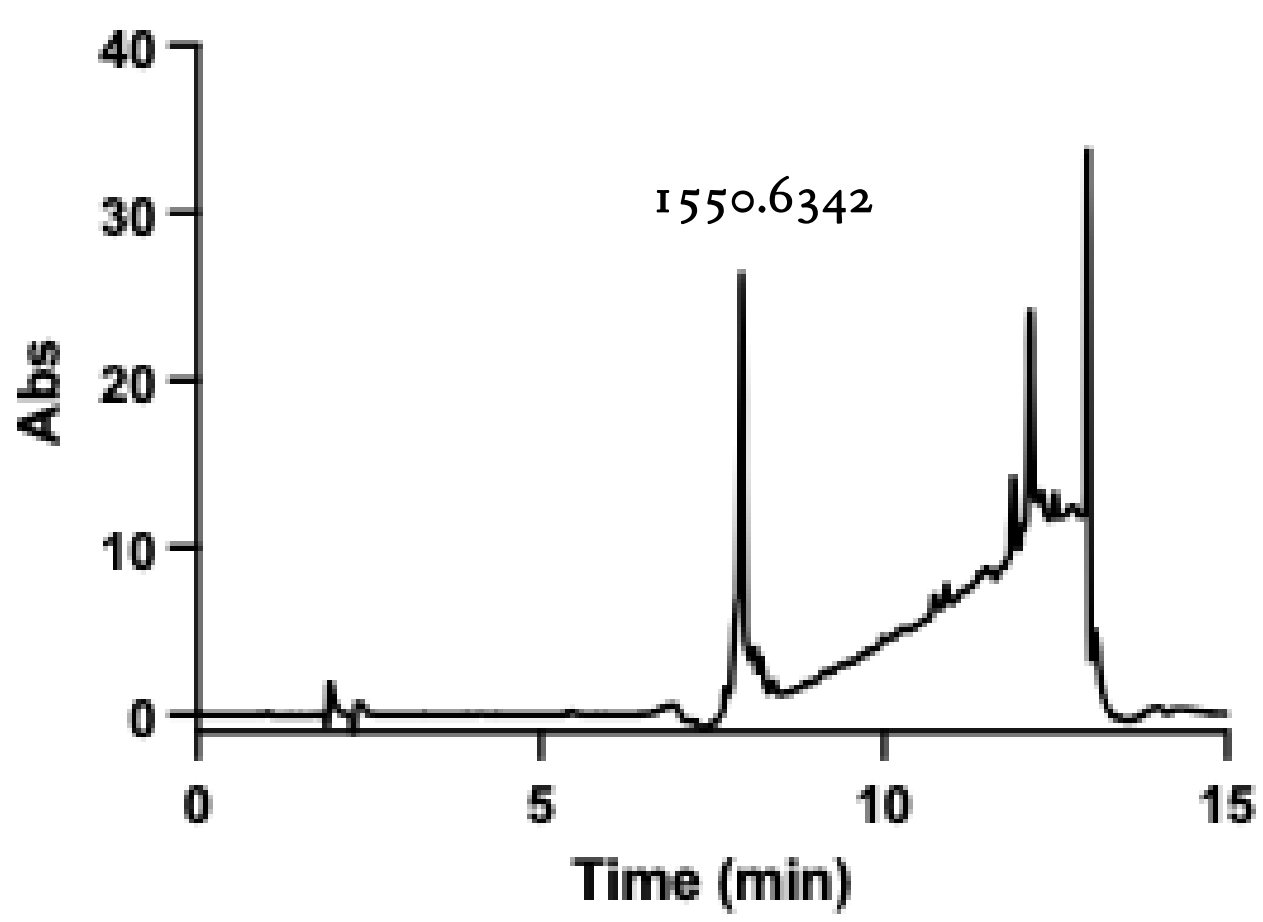

Chemical formula: $C_{72}H_{92}FN_{17}O_{15}S_3^+$

Calculated $[M + H]^+$ = 1550.81

Observed $[M + H]^+$ = 1550.6342

Compound 16

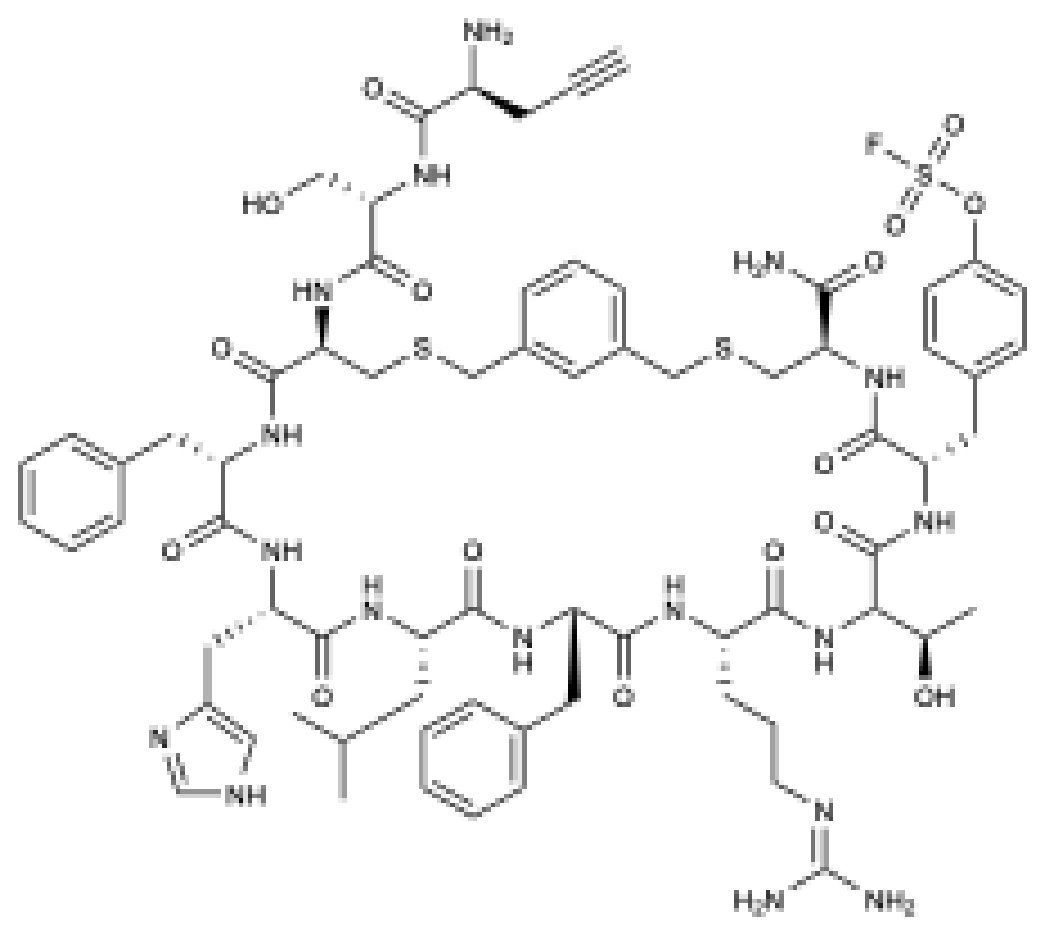

1554.6287

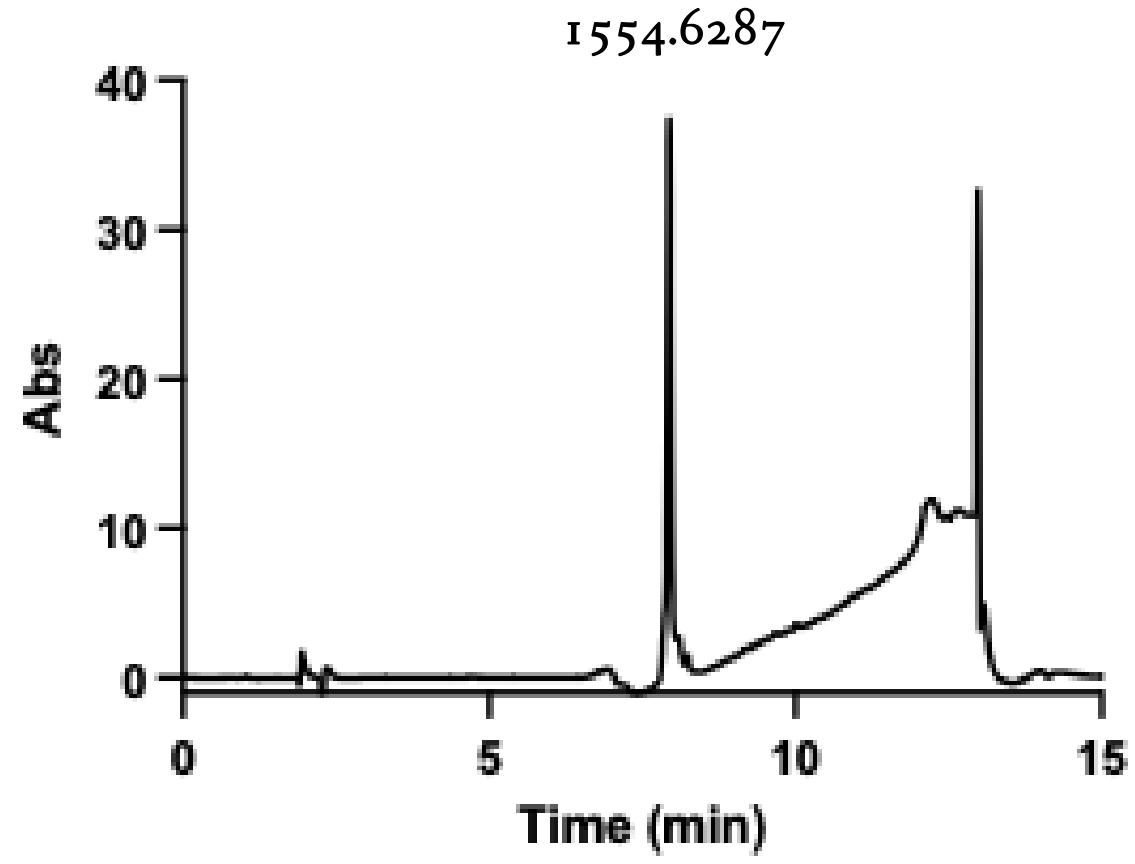

Chemical formula: $C_{71}H_{92}FN_{17}O_{16}S_3^+$

Calculated $[M + H]^+$ = 1554.80

Observed $[M + H]^+$ = 1554.6287

Compound 17

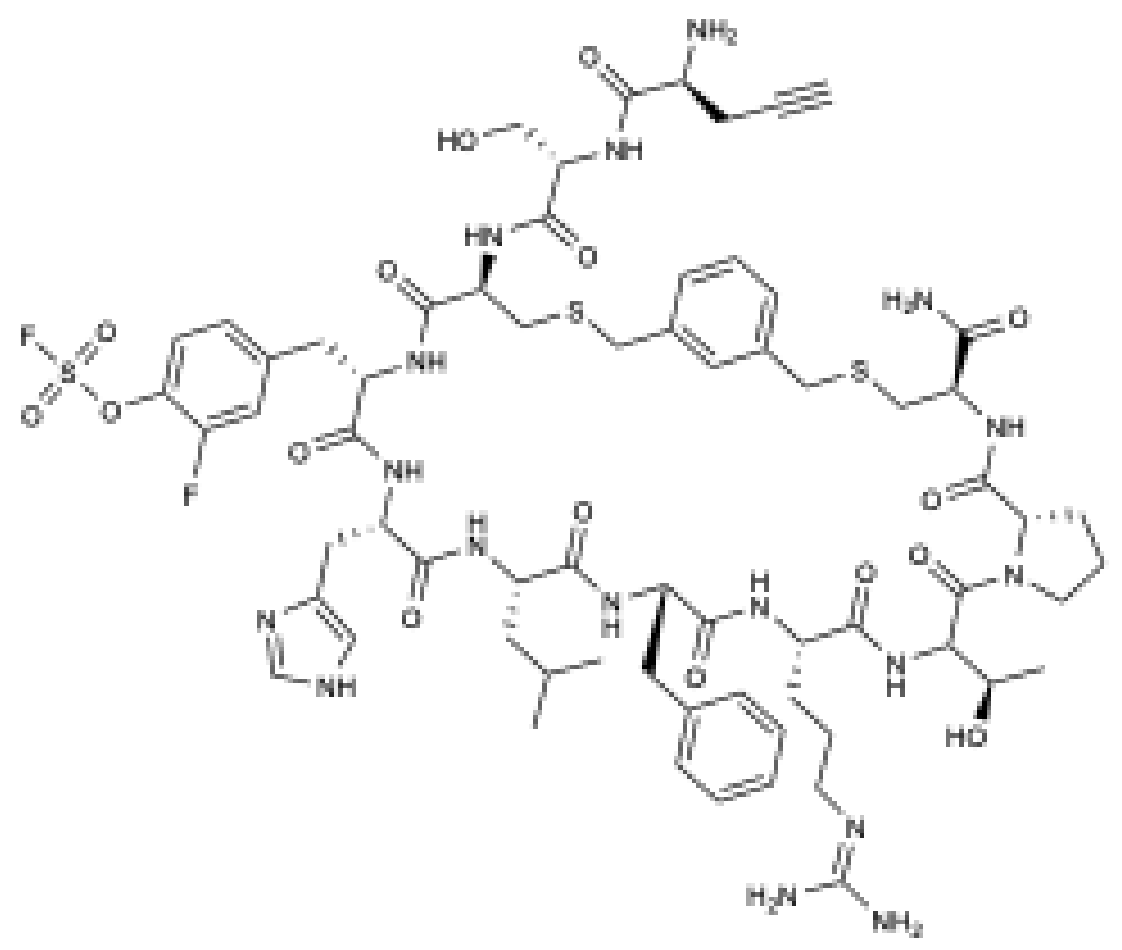

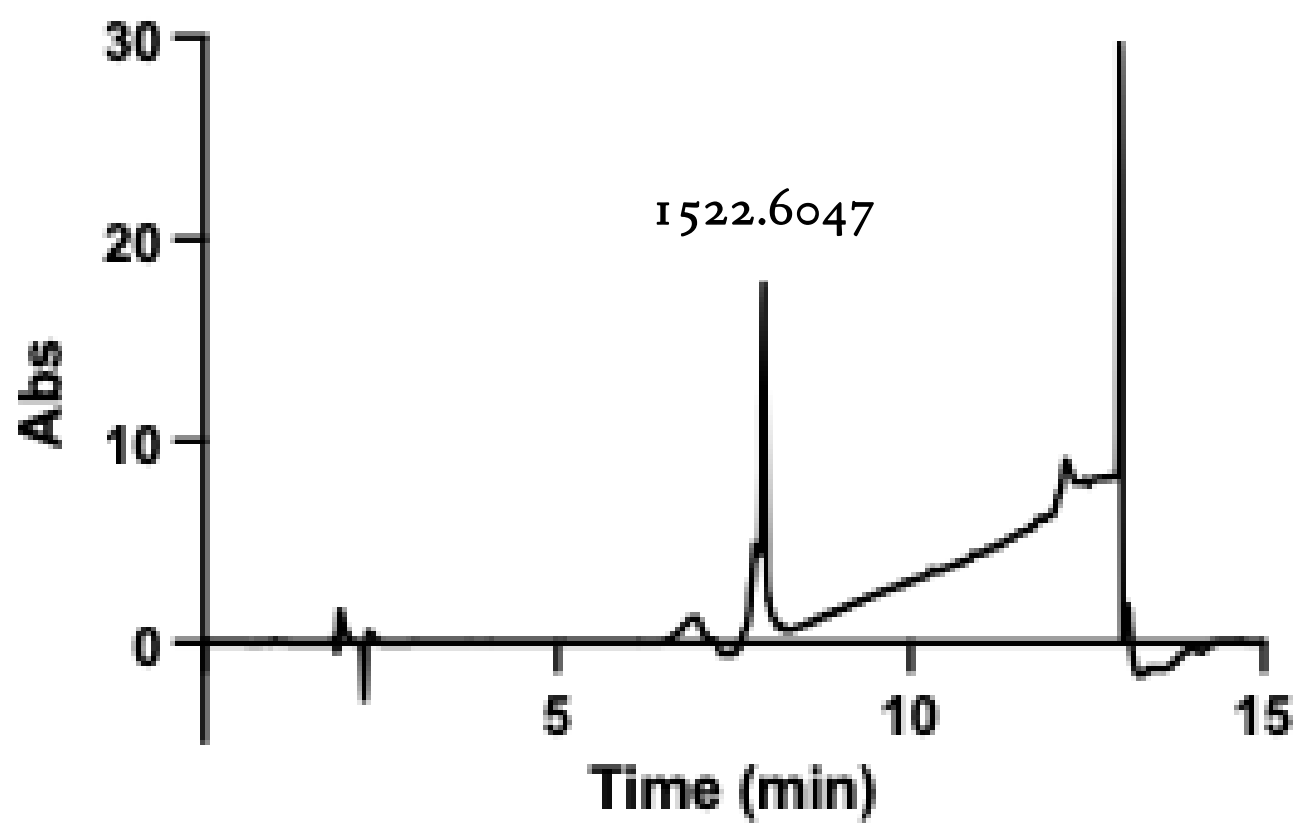

Chemical formula: $C_{67}H_{89}F_2N_{17}O_{16}S_3^+$

Calculated $[M + H]^+$ = 1522.73

Observed $[M + H]^+$ = 1522.6047

Compound 18

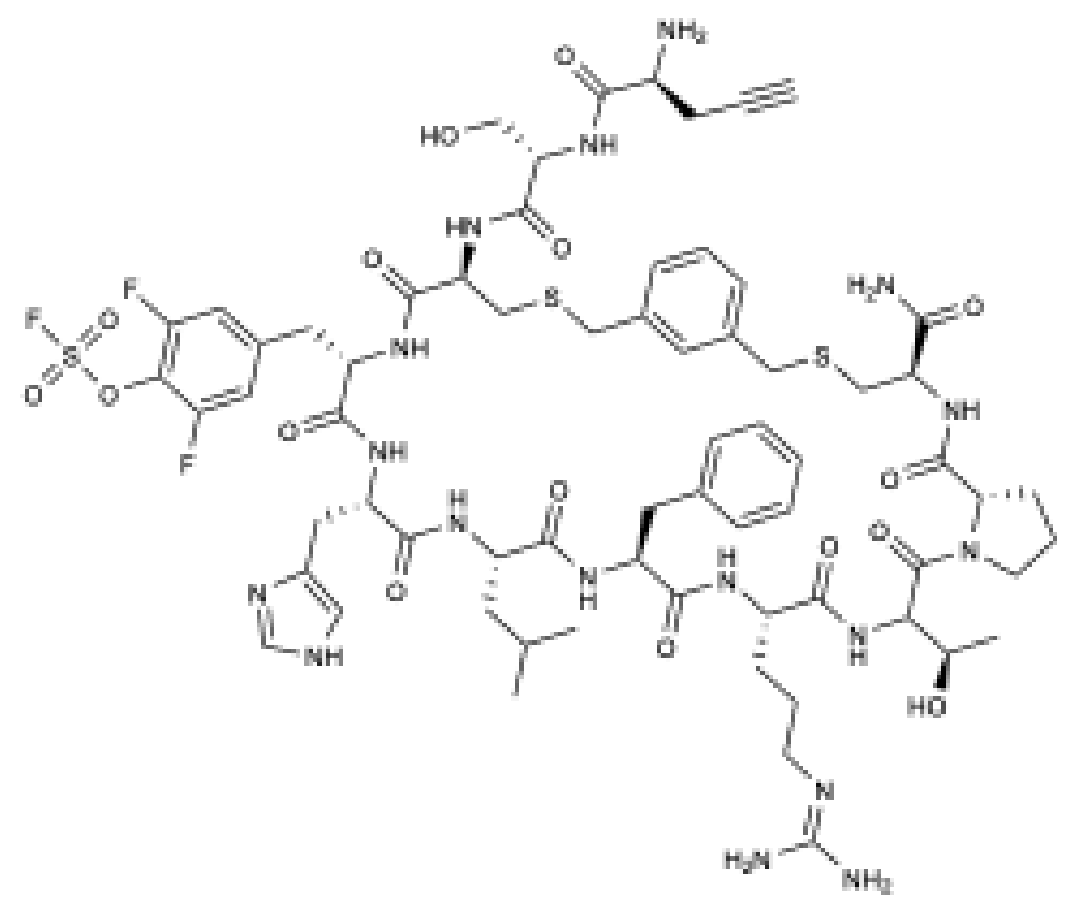

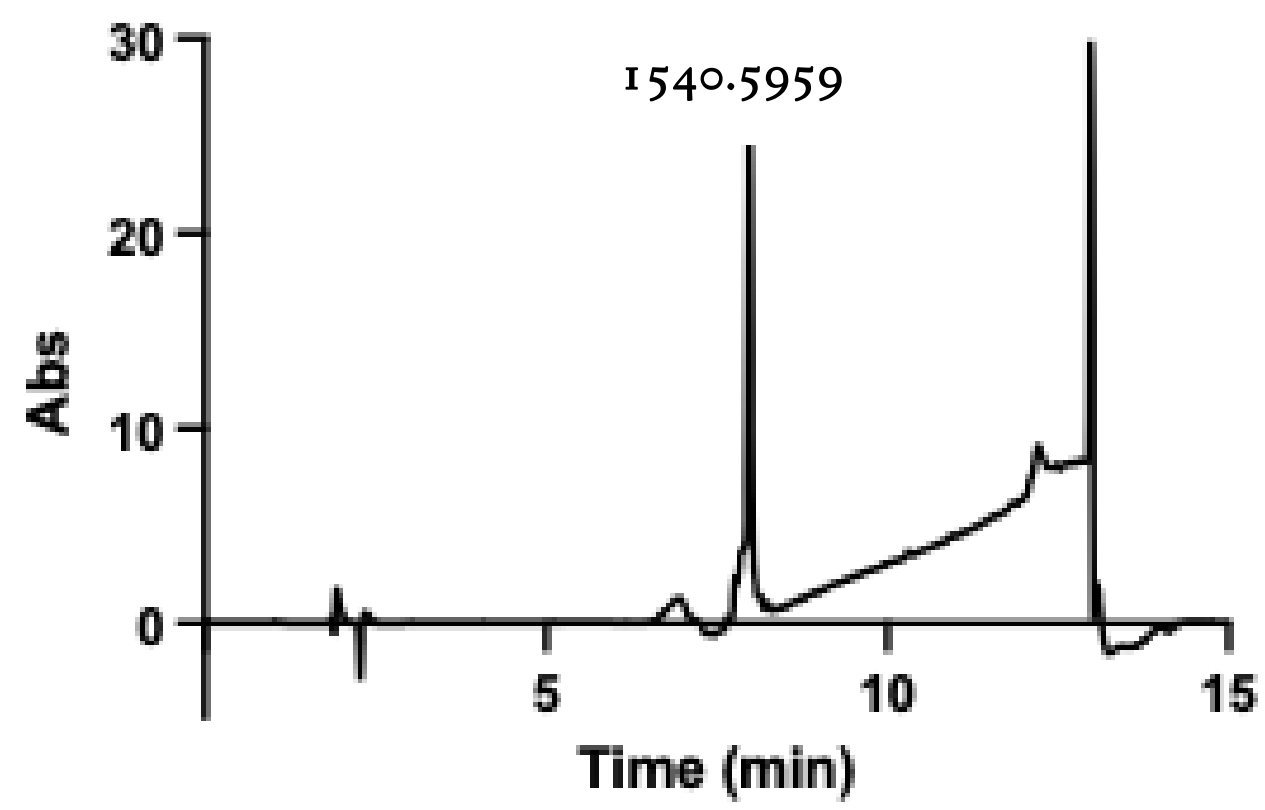

Chemical formula: $C_{67}H_{88}F_3N_{17}O_{16}S_3^+$

Calculated $[M + H]^+ = 1540.72$

Observed $[M + H]^+ = 1540.5959$

Compound 19

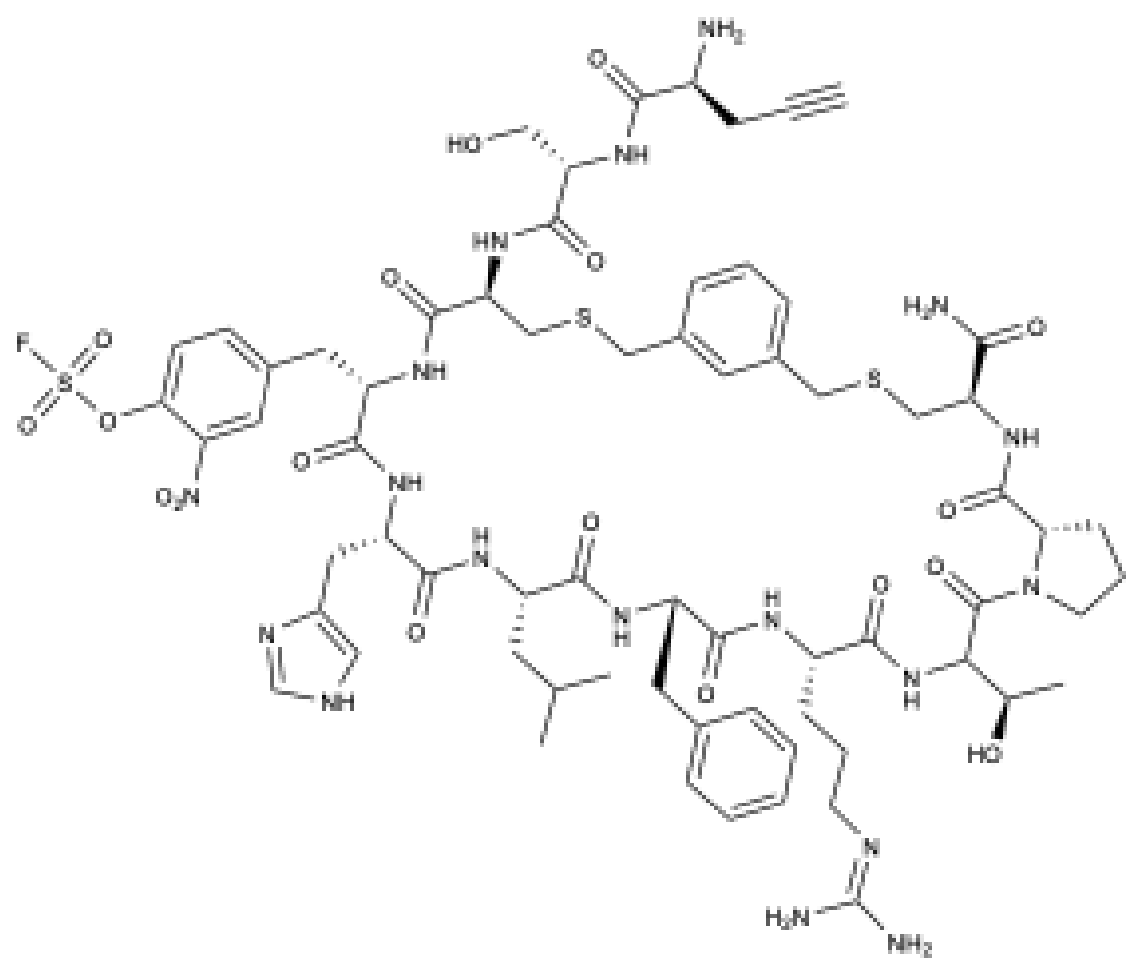

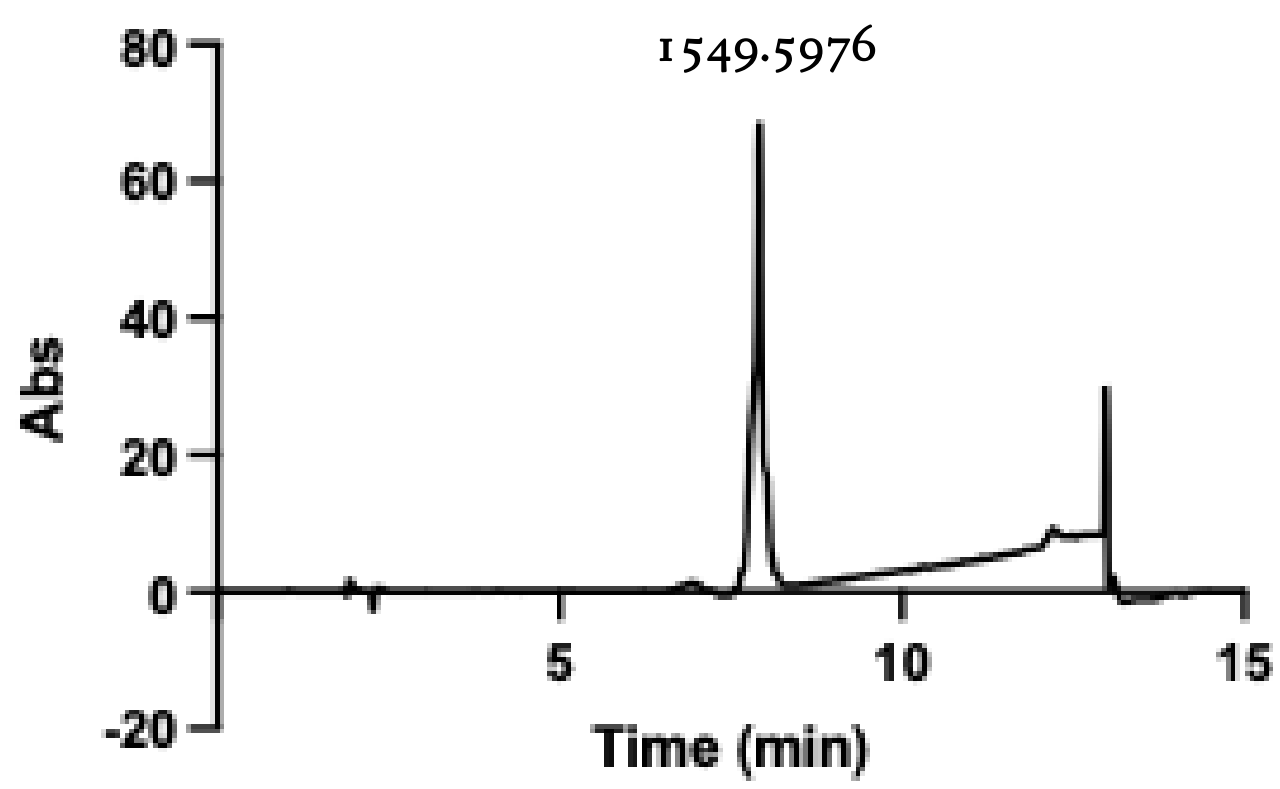

Chemical formula: $C_{67}H_{89}FN_{18}O_{18}S_3^+$

Calculated $[M + H]^+ = 1549.74$

Observed $[M + H]^+ = 1549.5976$

Compound 20

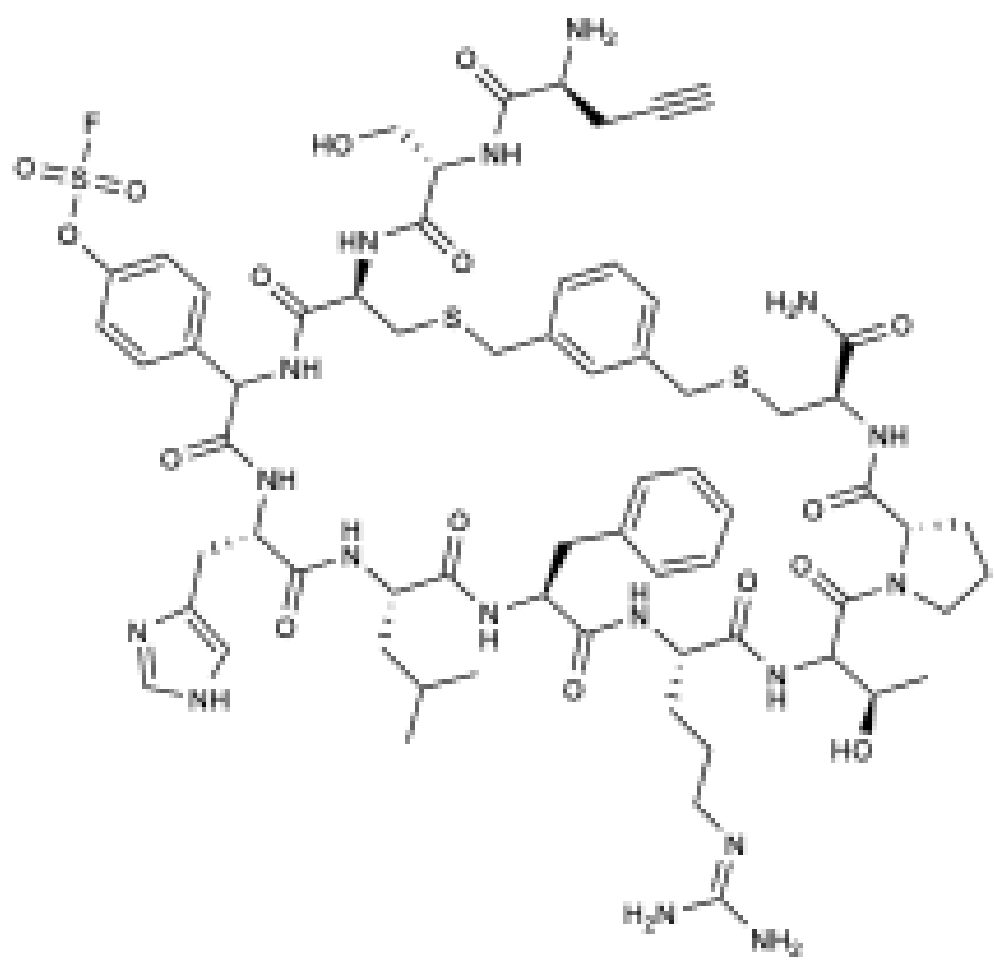

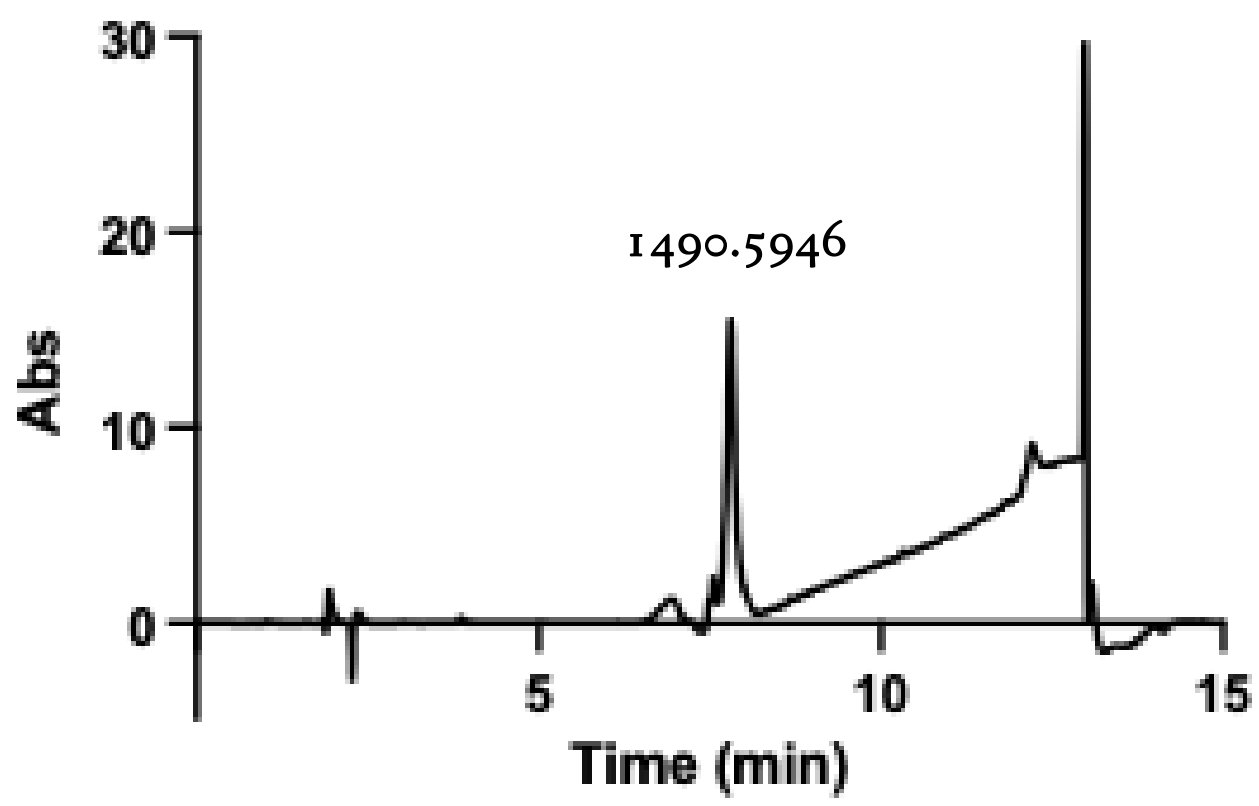

Chemical formula: $C_{66}H_{88}FN_{17}O_{16}S_3^{+}$

Calculated $[M + H]^+ = 1490.71$

Observed $[M + H]^+ = 1490.5946$

Compound 21

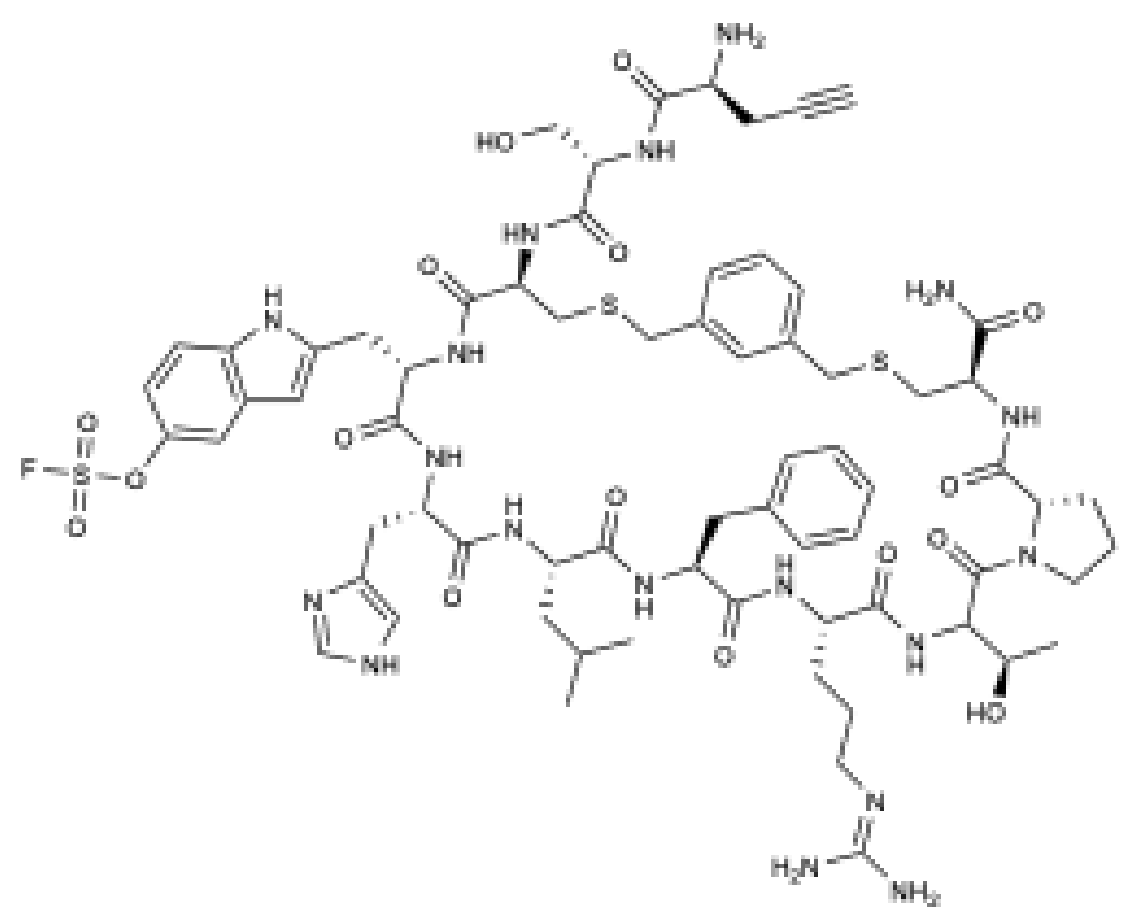

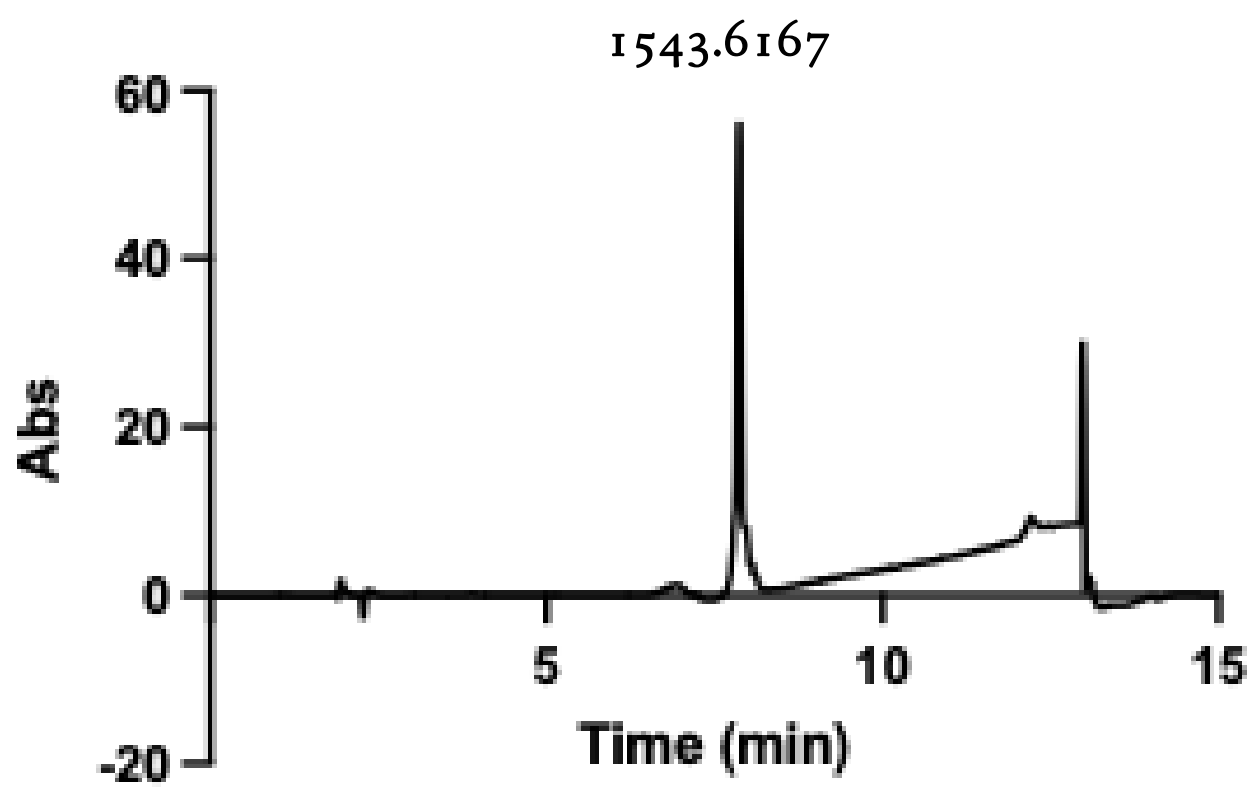

Chemical formula: $C_{69}H_{91}FN_{18}O_{16}S_3^+$

Calculated $[M + H]^+$ = 1543.78

Observed $[M + H]^+$ = 1543.6167

Compound 22

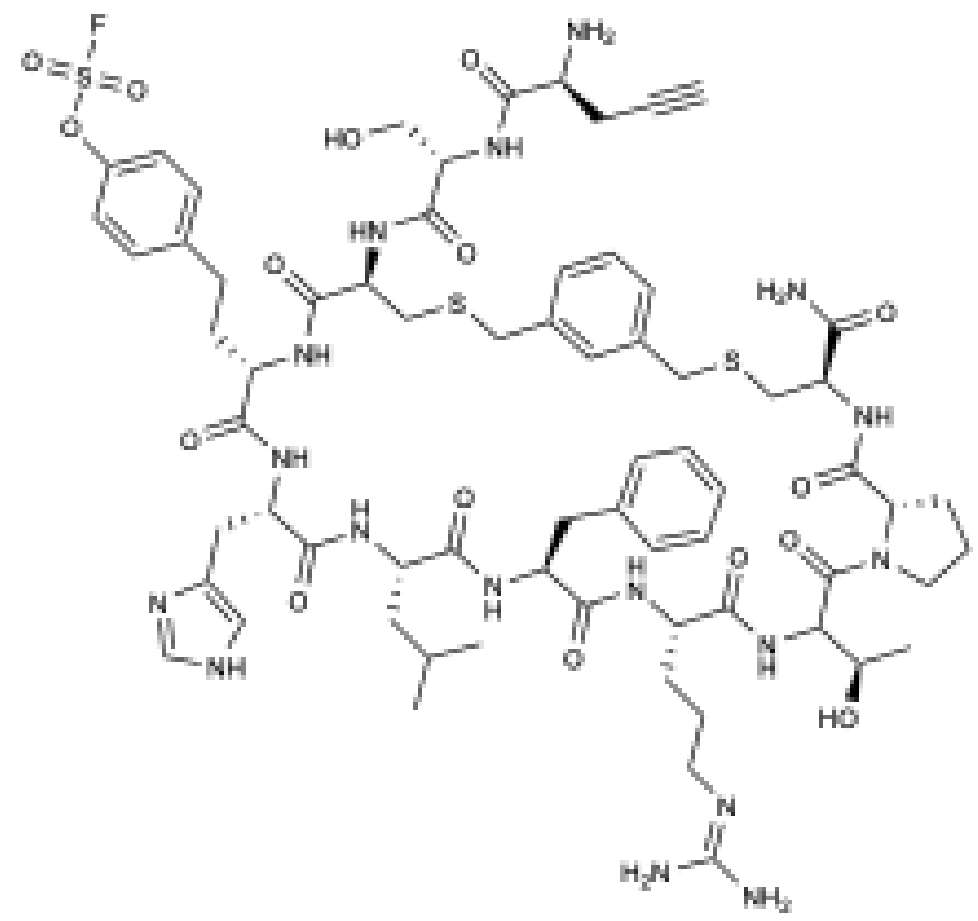

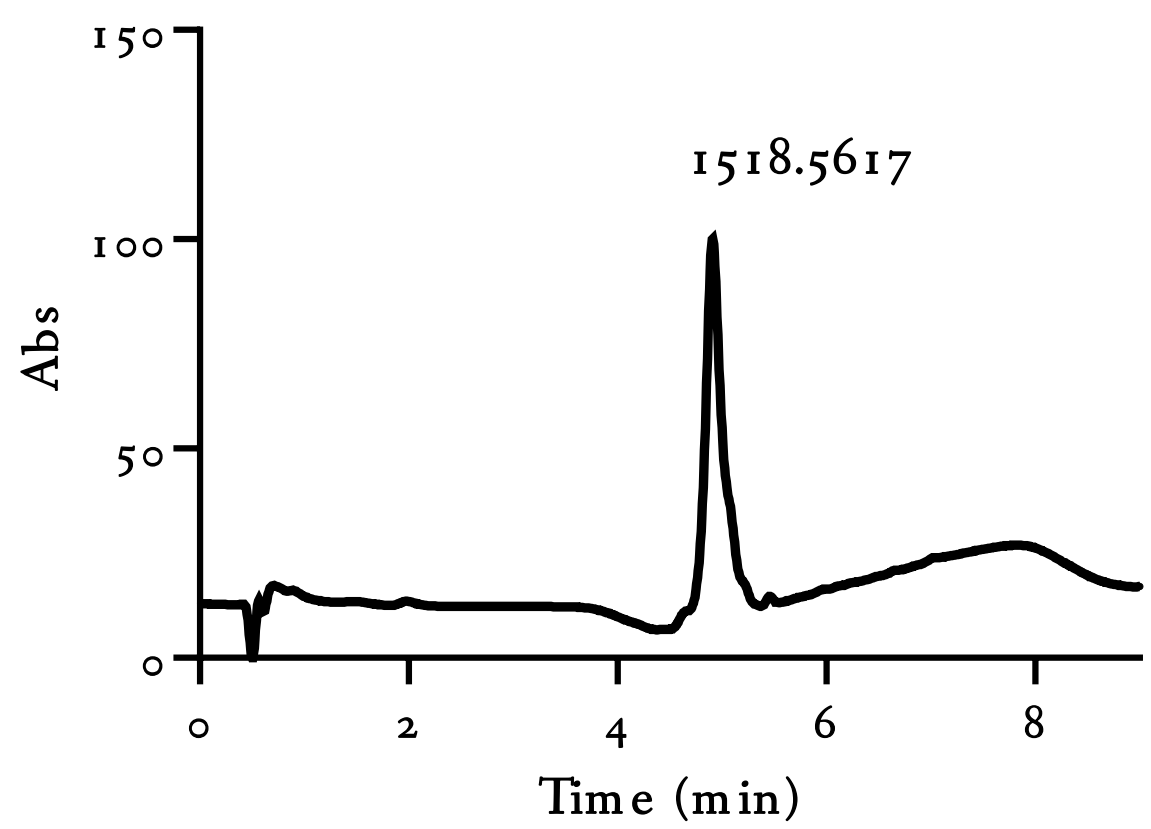

Calculated $[M + 2H]^{2+}$ = 1518.56

Observed $[M + 2H]^{2+}$ = 1518.5617

Compound 23

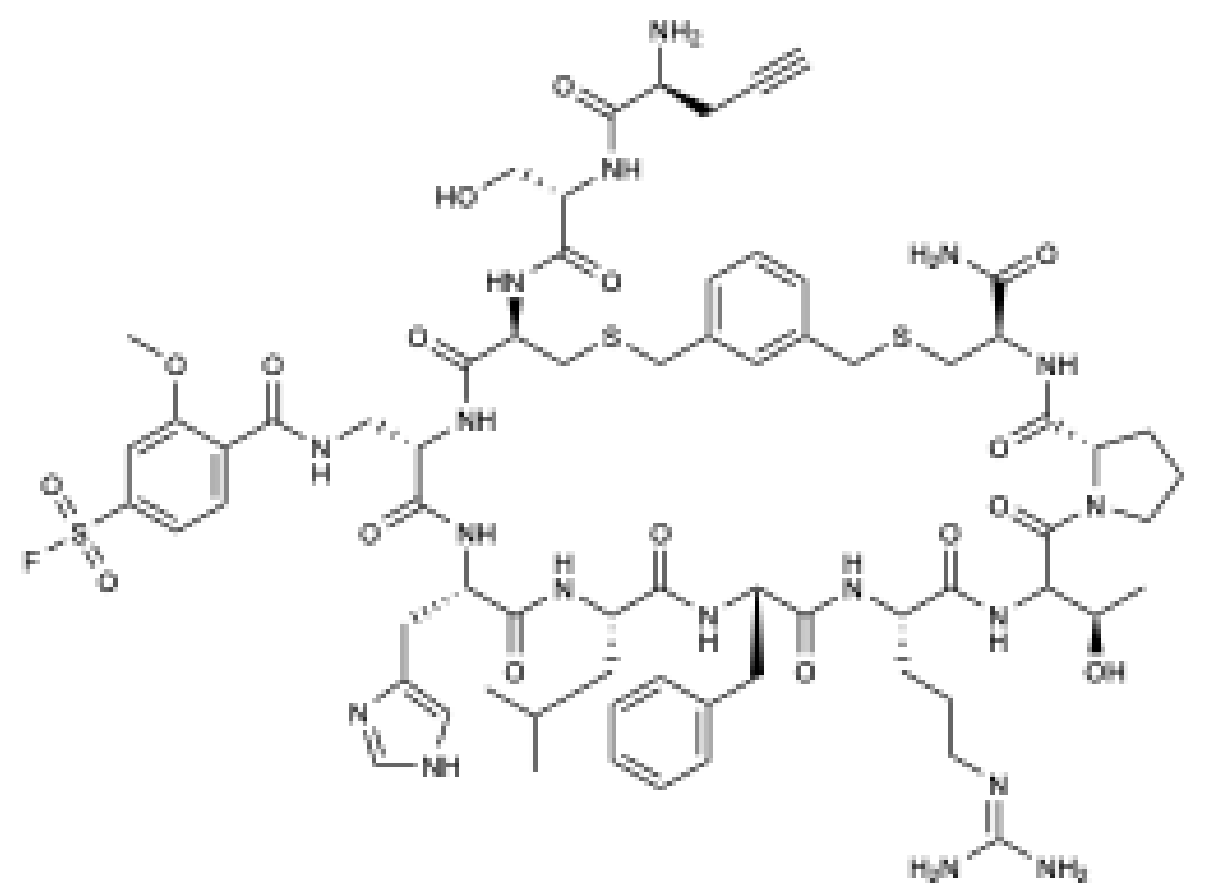

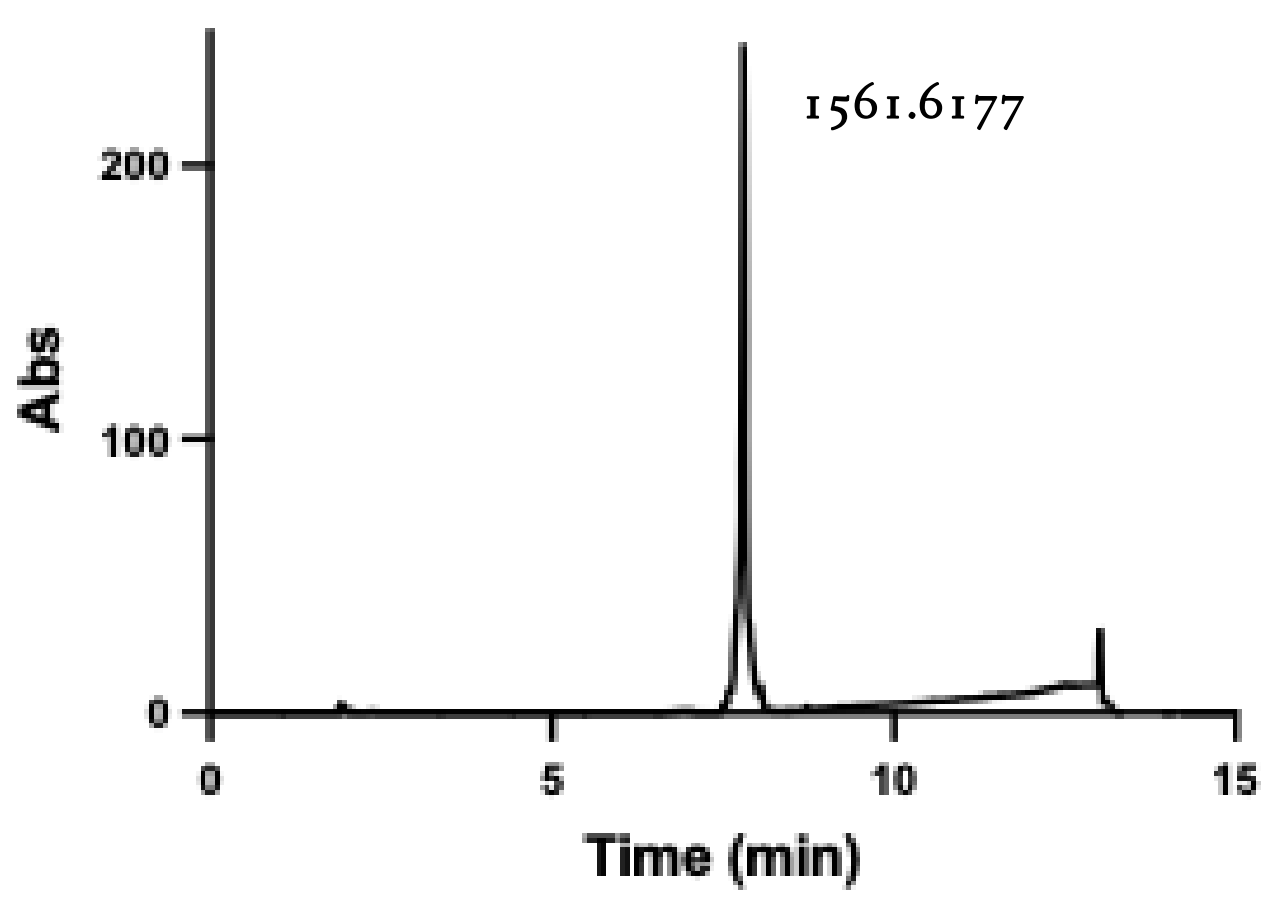

Chemical formula: $C_{69}H_{93}FN_{18}O_{17}S_3^+$

Calculated $[M + H]^+$ = 1561.79

Observed $[M + H]^+$ = 1561.6177

Compound 24

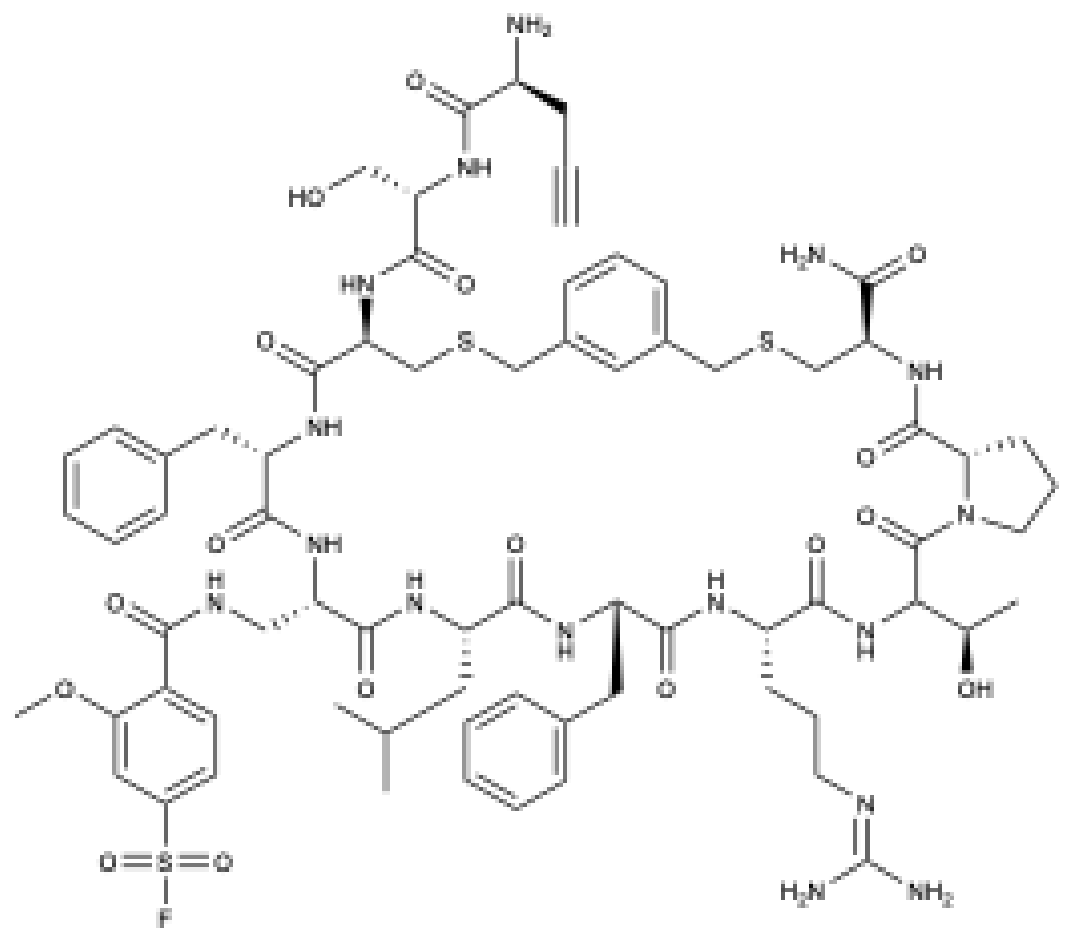

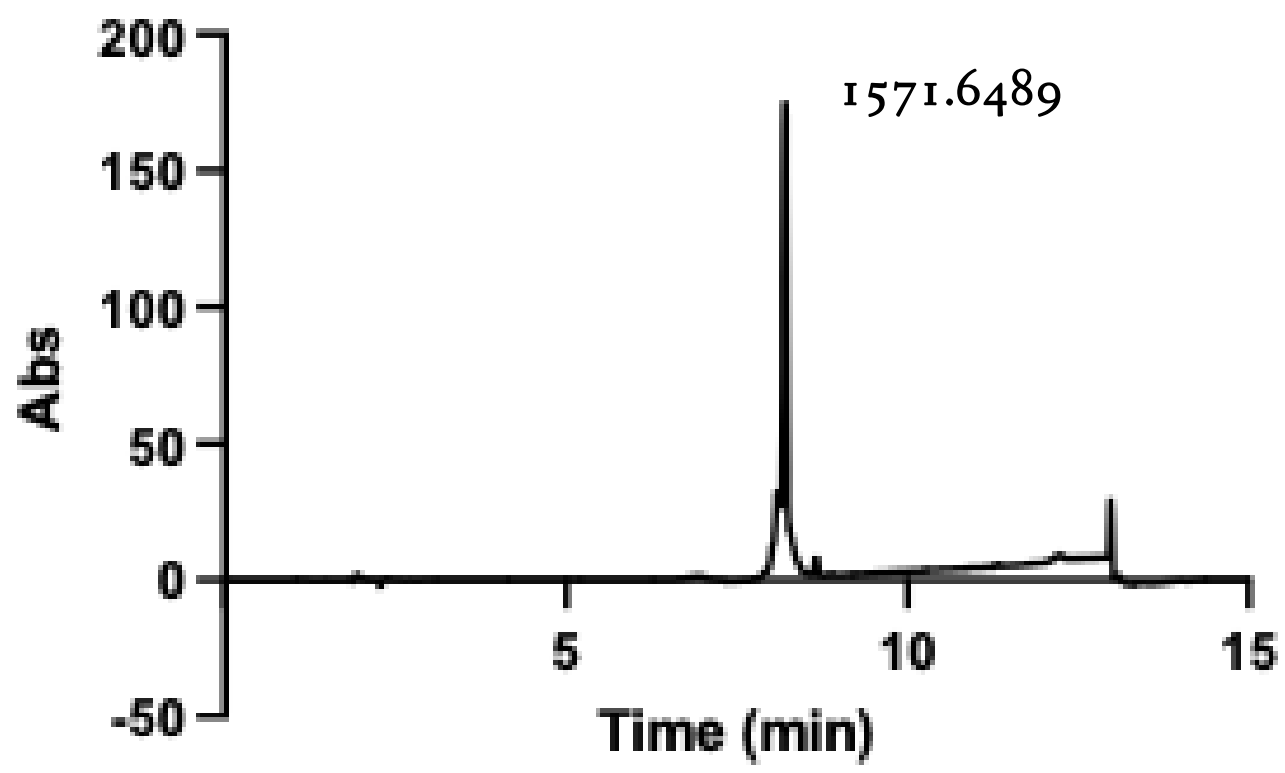

Chemical formula: $C_{72}H_{95}FN_{16}O_{17}S_3^+$

Calculated $[M + H]^+ = 1571.83$

Observed $[M + H]^+ = 1571.6489$

Compound 25

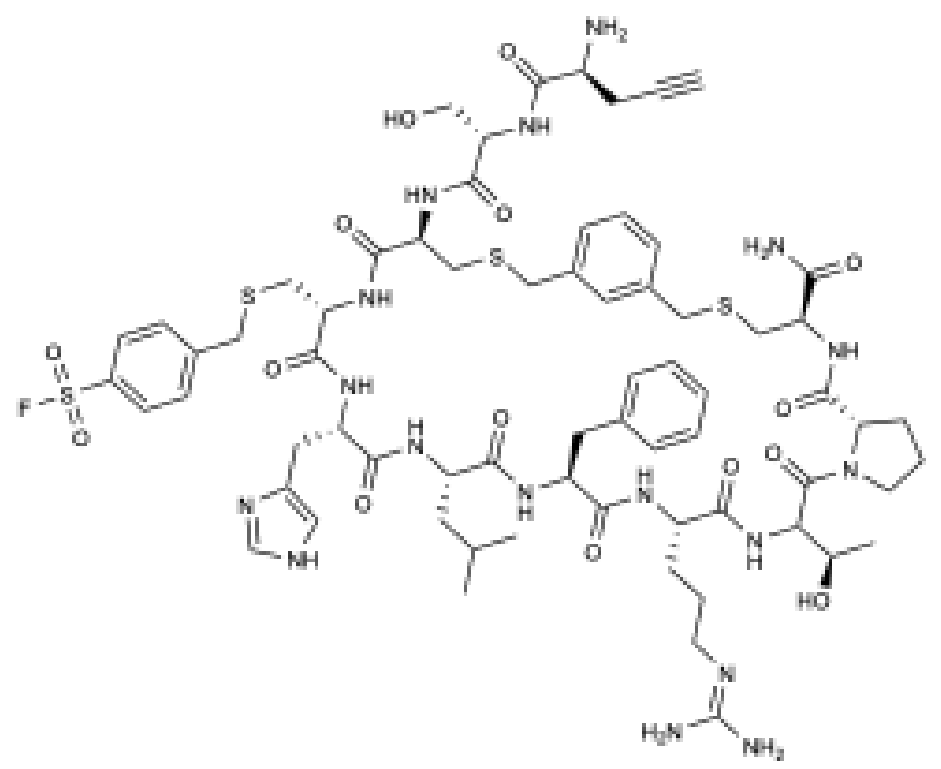

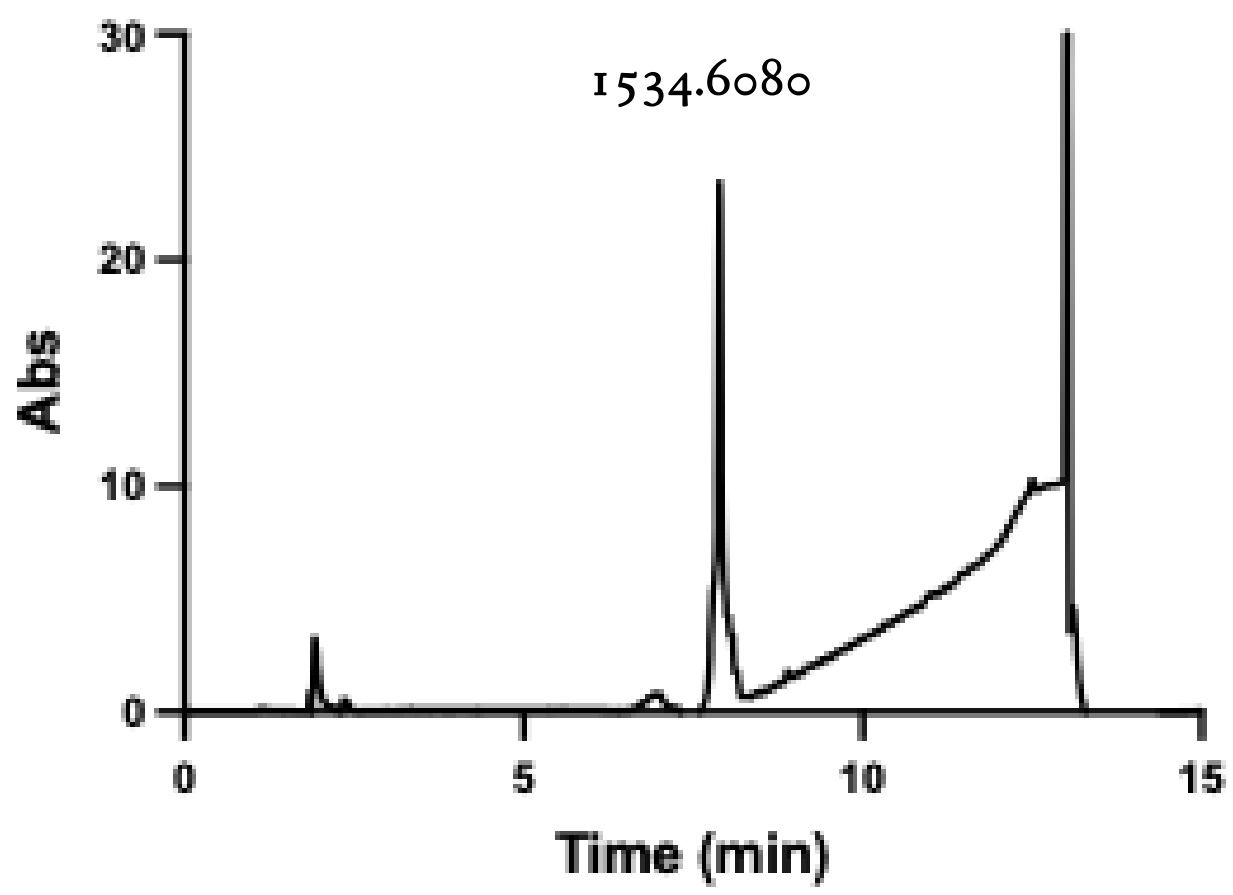

Chemical formula: $C_{68}H_{92}FN_{17}O_{15}S_4^+$

Calculated $[M + H]^+$ = 1534.83

Observed $[M + H]^+$ = 1534.6080

Compound 26

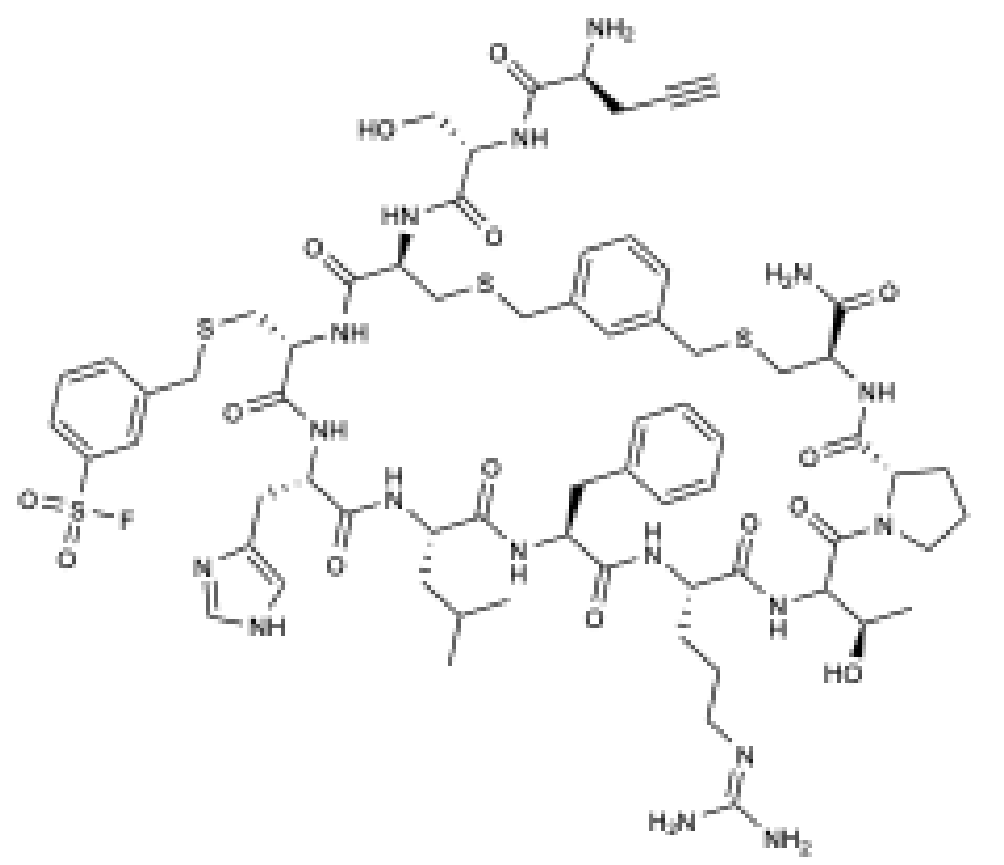

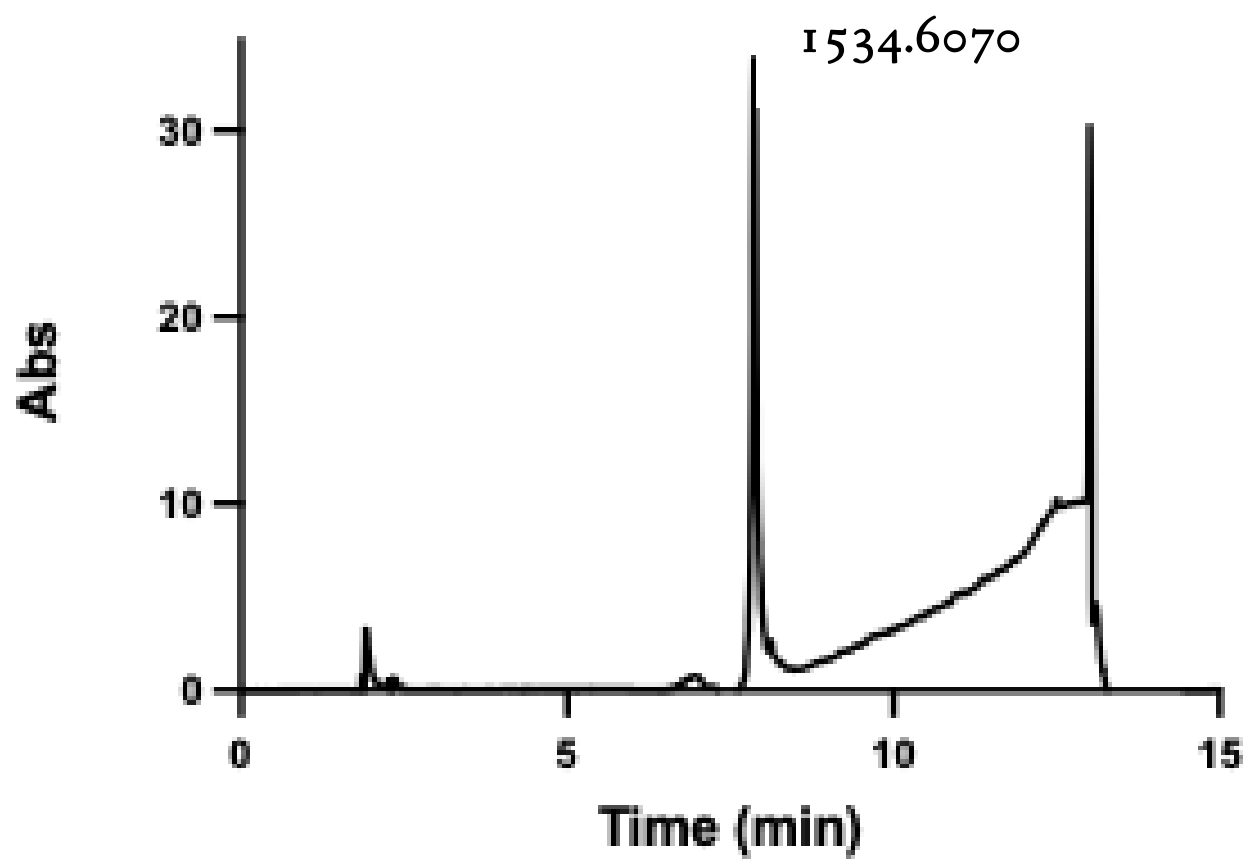

Chemical formula: $C_{68}H_{92}FN_{17}O_{15}S_4^+$

Calculated $[M + H]^+ = 1534.83$

Observed $[M + H]^+ = 1534.6070$

Compound 27

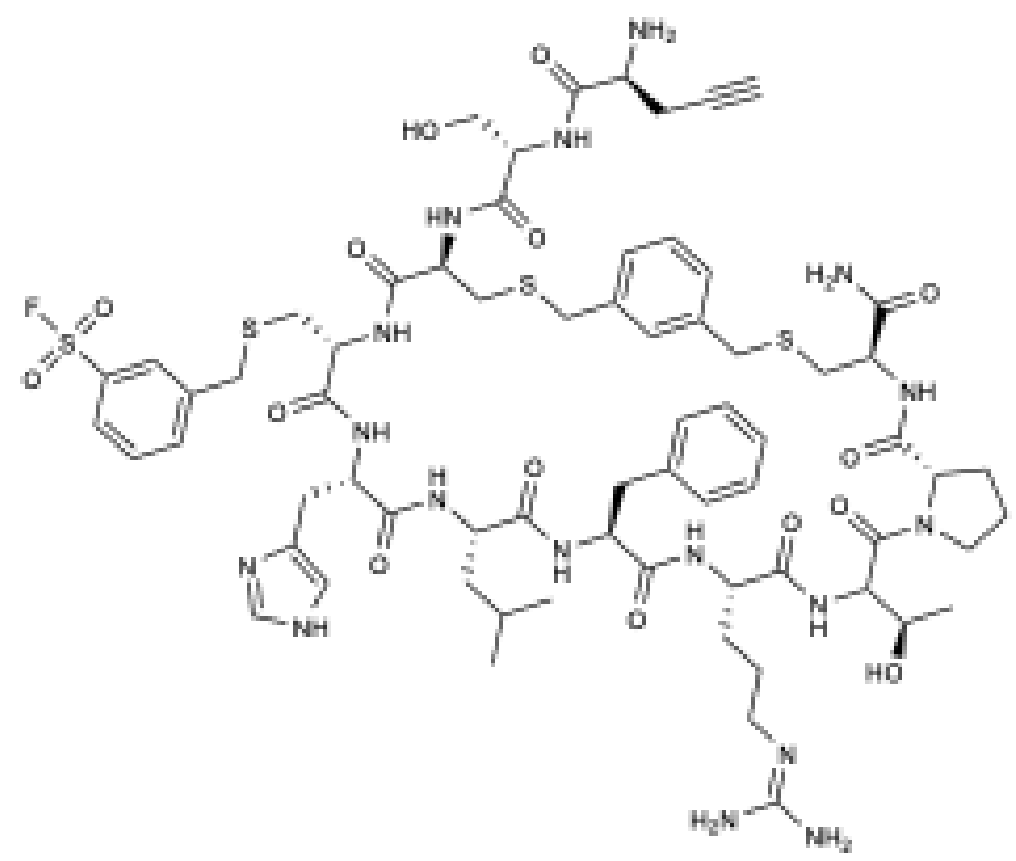

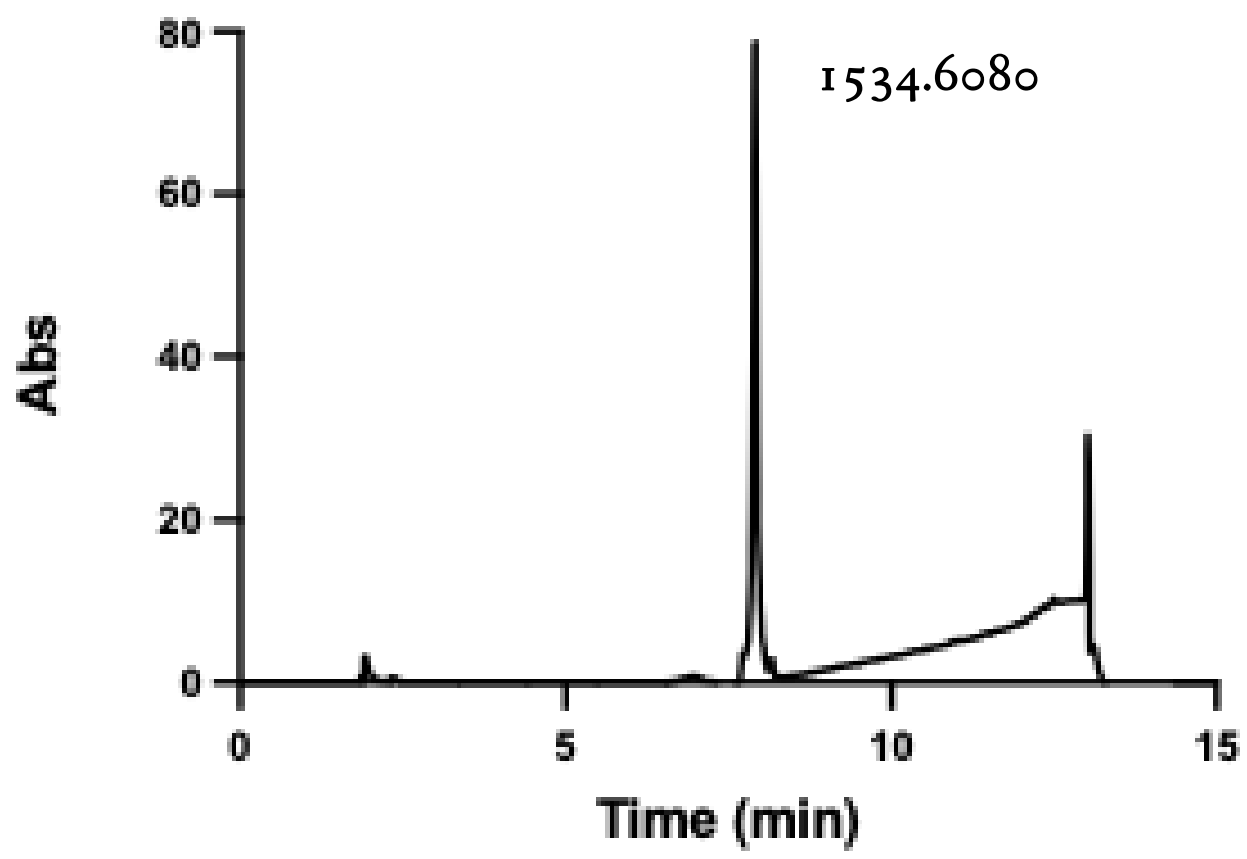

Chemical formula: $C_{68}H_{92}FN_{17}O_{15}S_4^+$

Calculated [M + H]+ = 1534.83

Observed [M + H]+ = 1534.6080

Compound 28

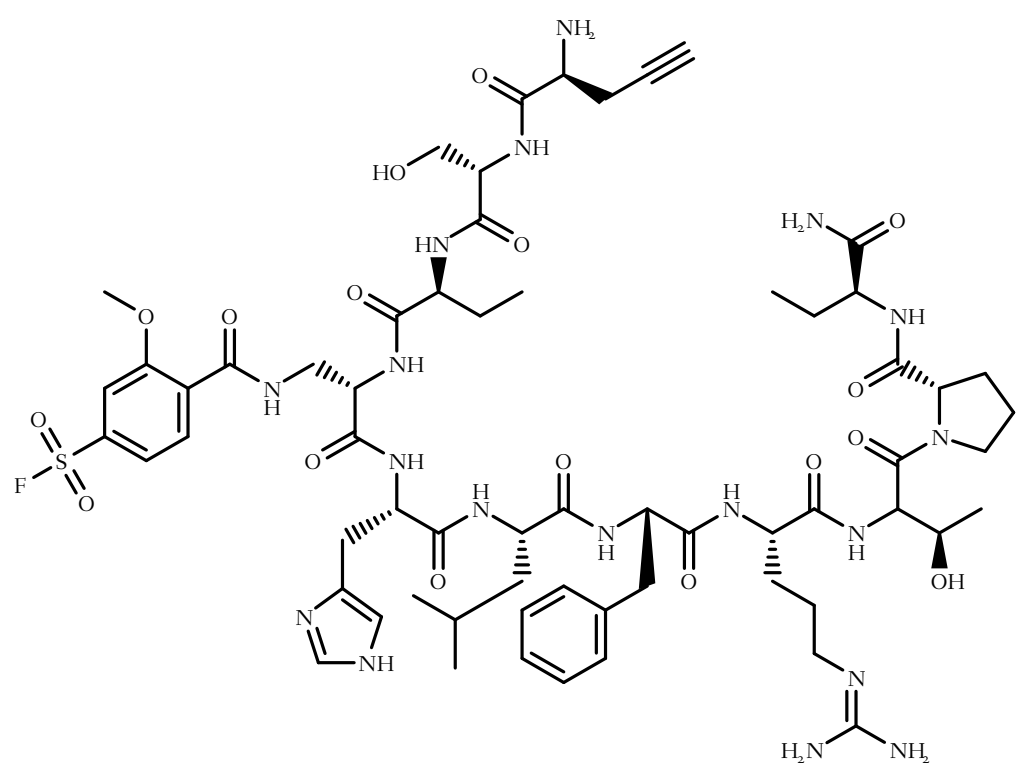

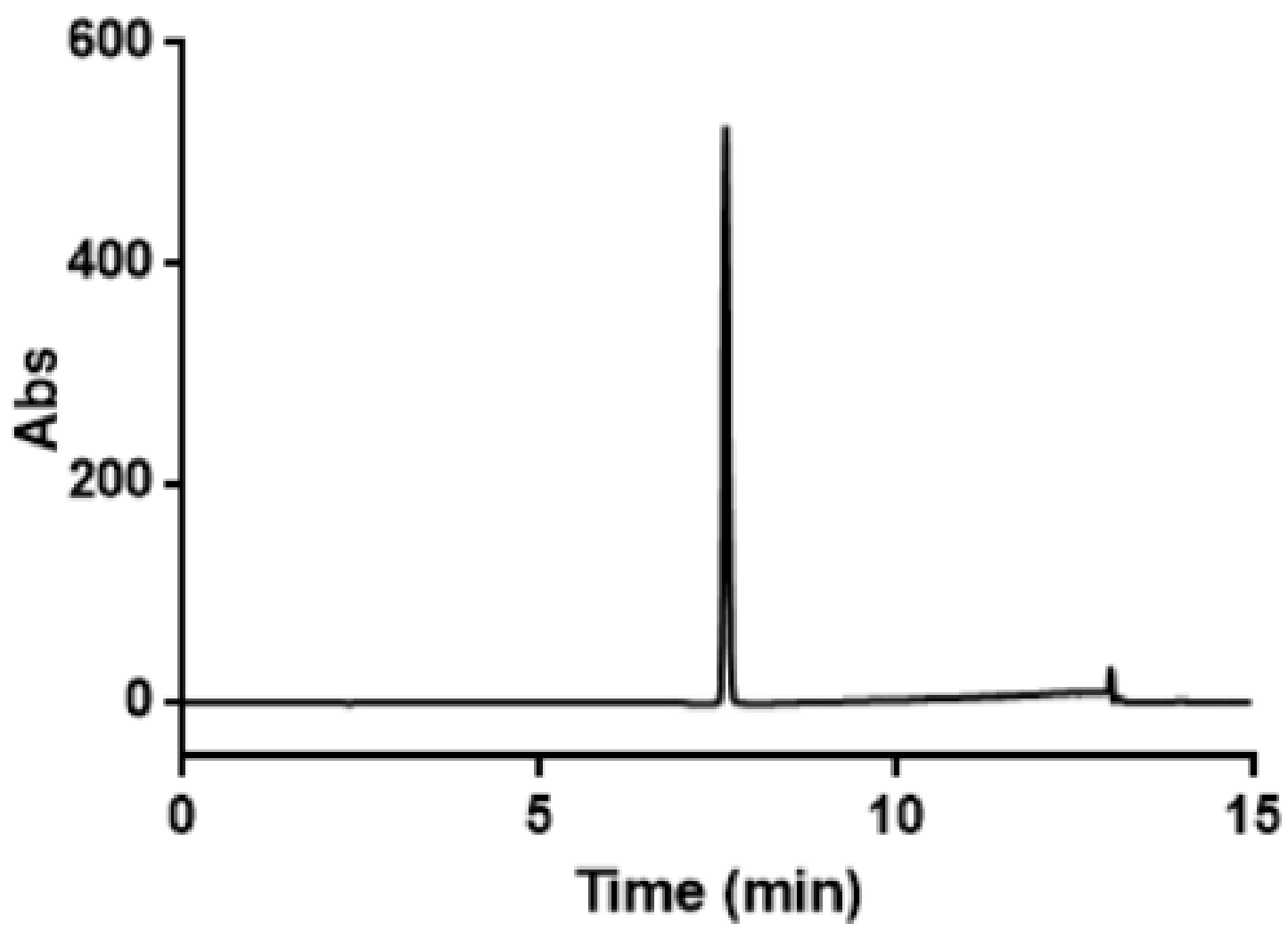

Chemical Formula: $C_{63}H_{91}FN_{18}O_{17}S$

Calculated $[M + 2H]^{2+}$ = 712.85

Observed $[M + 2H]^{2+}$ = 712.8591

Compound 29

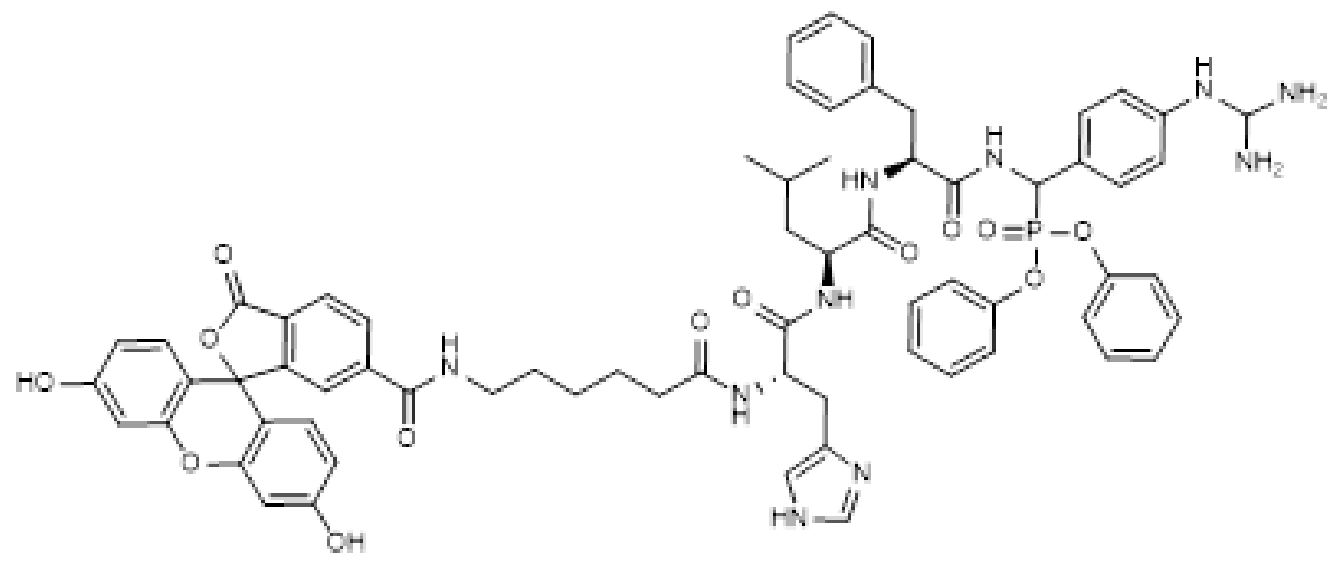

Chemical Formula: $C_{67}H_{69}N_{10}O_{11}P$

Calculated $[M + 2H]^{2+}$ = 633.24

Observed $[M + 2H]^{2+}$ = 633.2466 // 633.2409

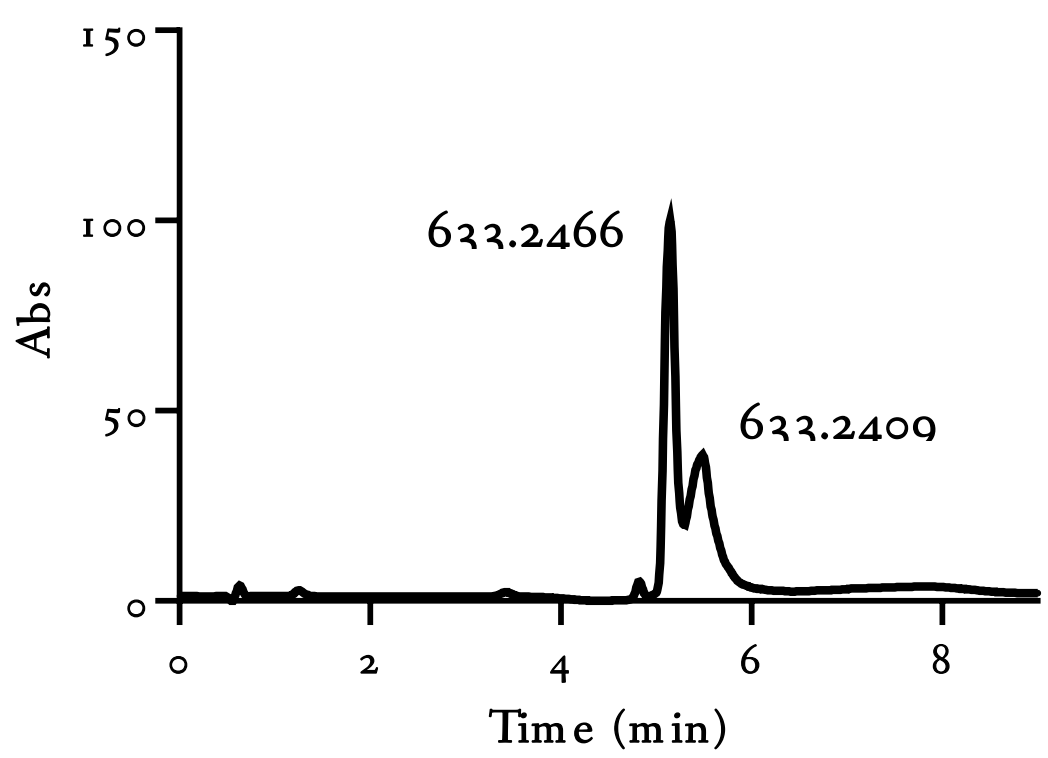

Compound 30

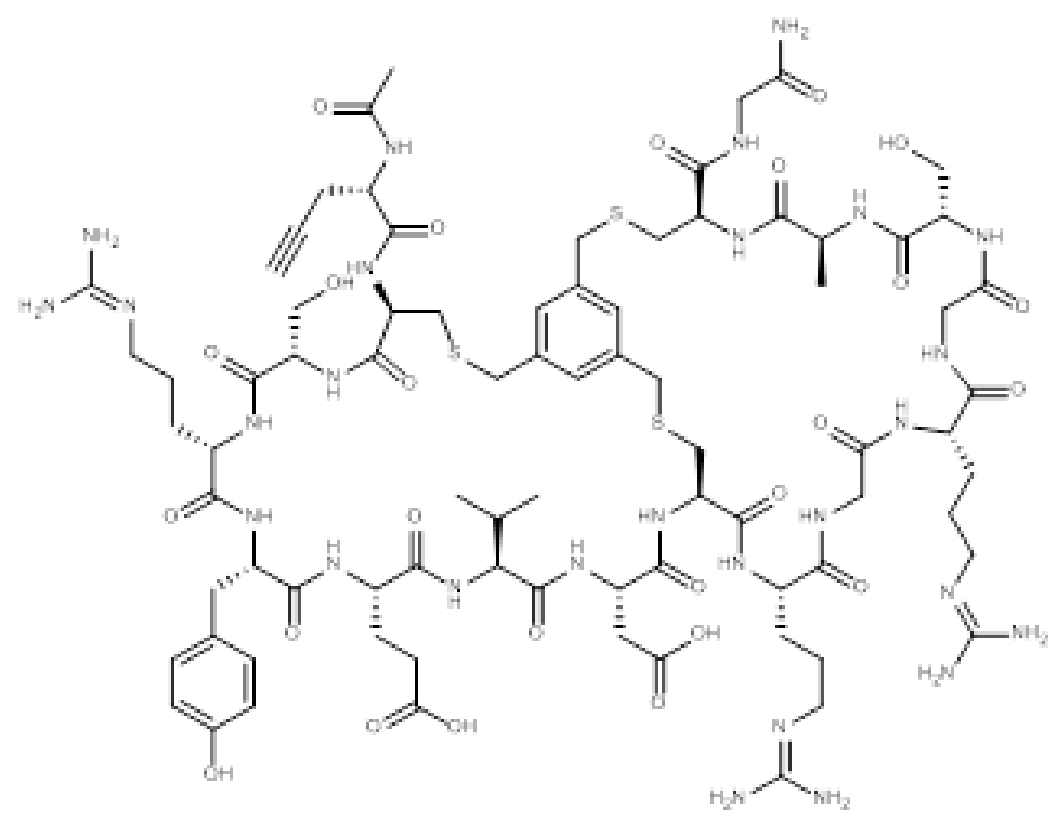

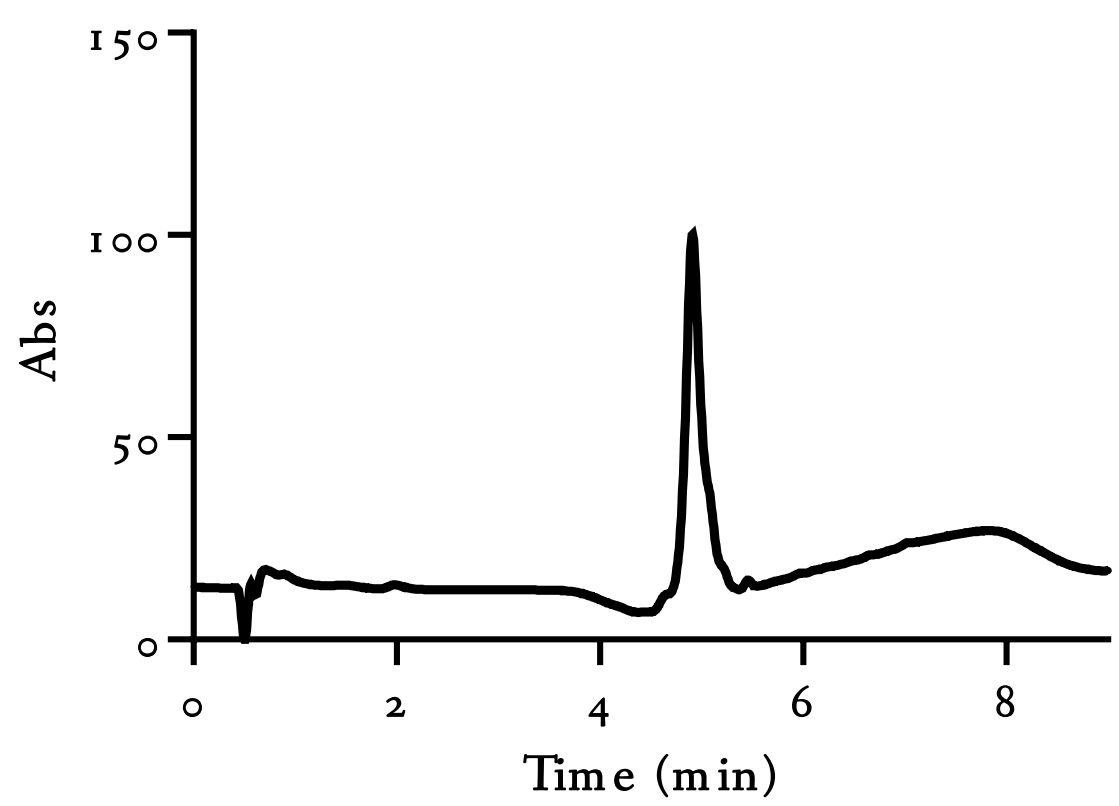

Calculated $[M + 2H]^{2+} = 985.41$

Observed $[M + 2H]^{2+} = 985.4191$

Compound 31

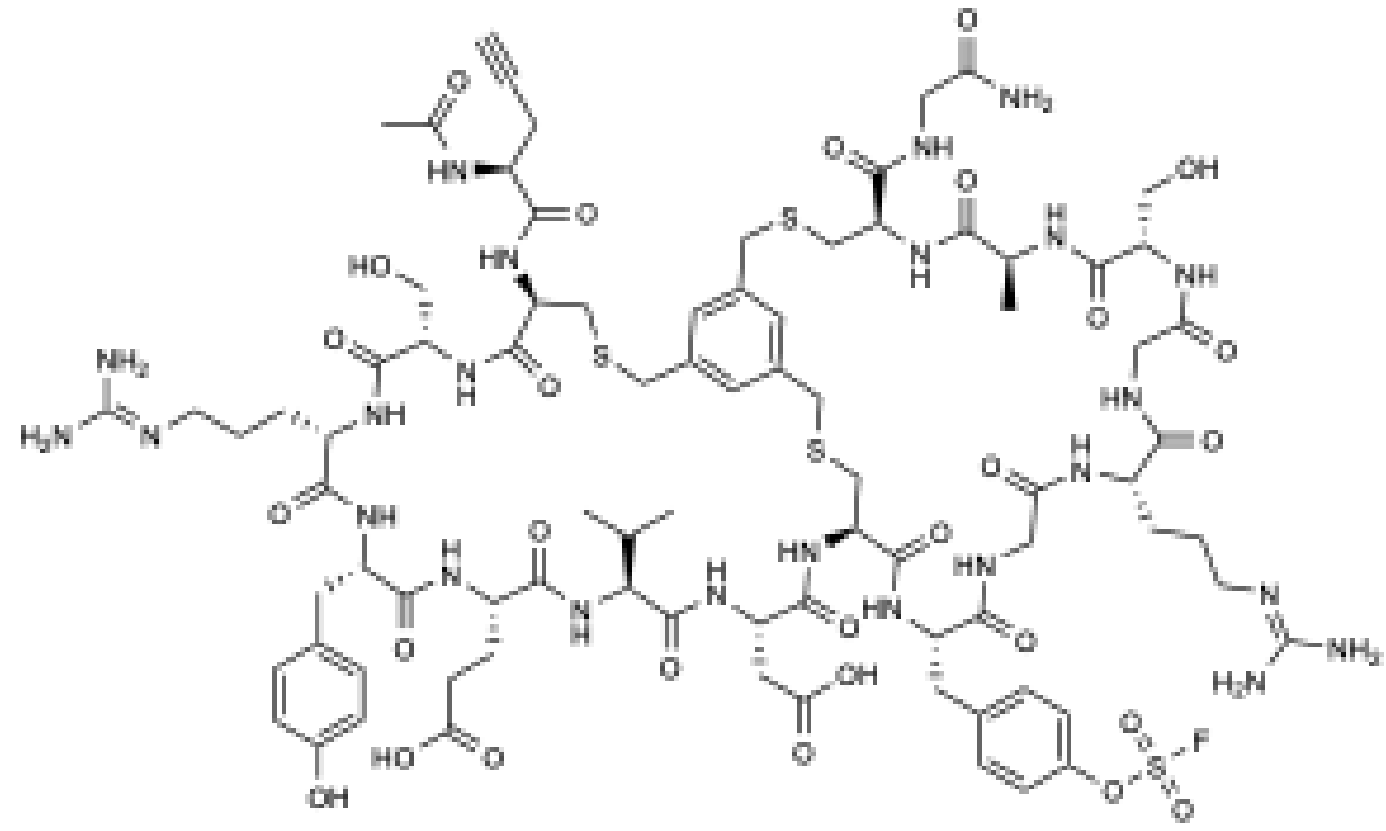

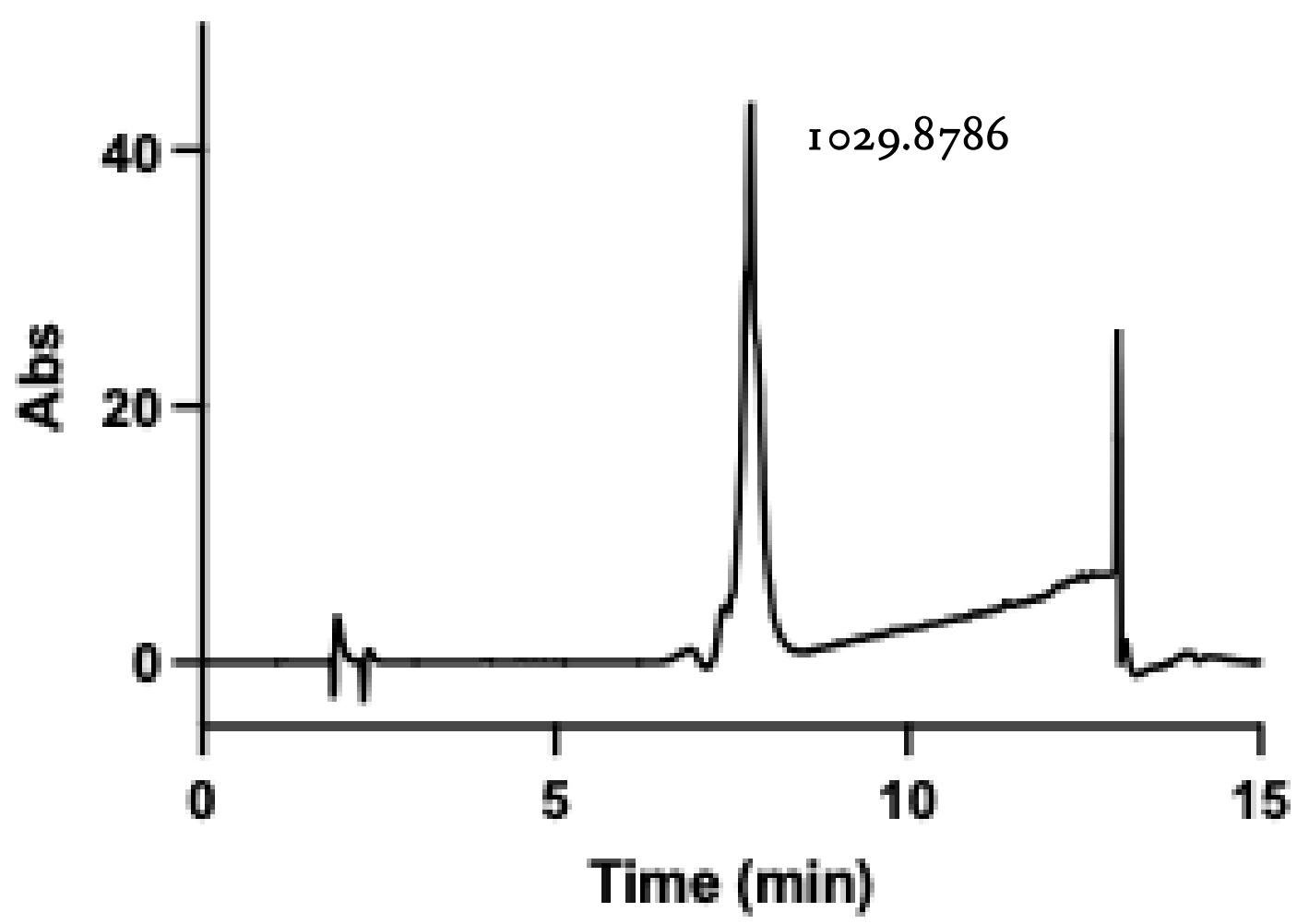

Chemical formula: $C_{84}H_{117}FN_{24}O_{28}S_4^{2+}$

Calculated $[M + 2H]^{2+}$ = 1029.36

Observed $[M + 2H]^{2+}$ = 1029.8786

Compound 32

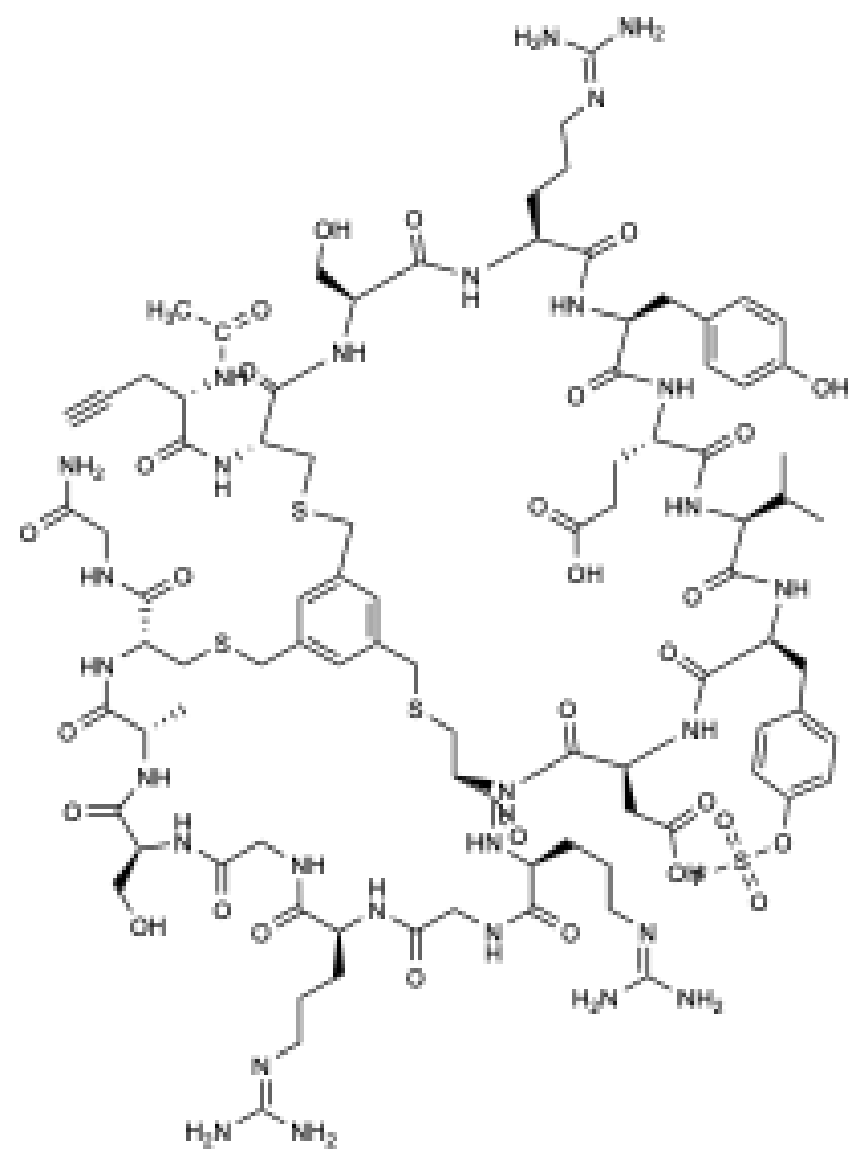

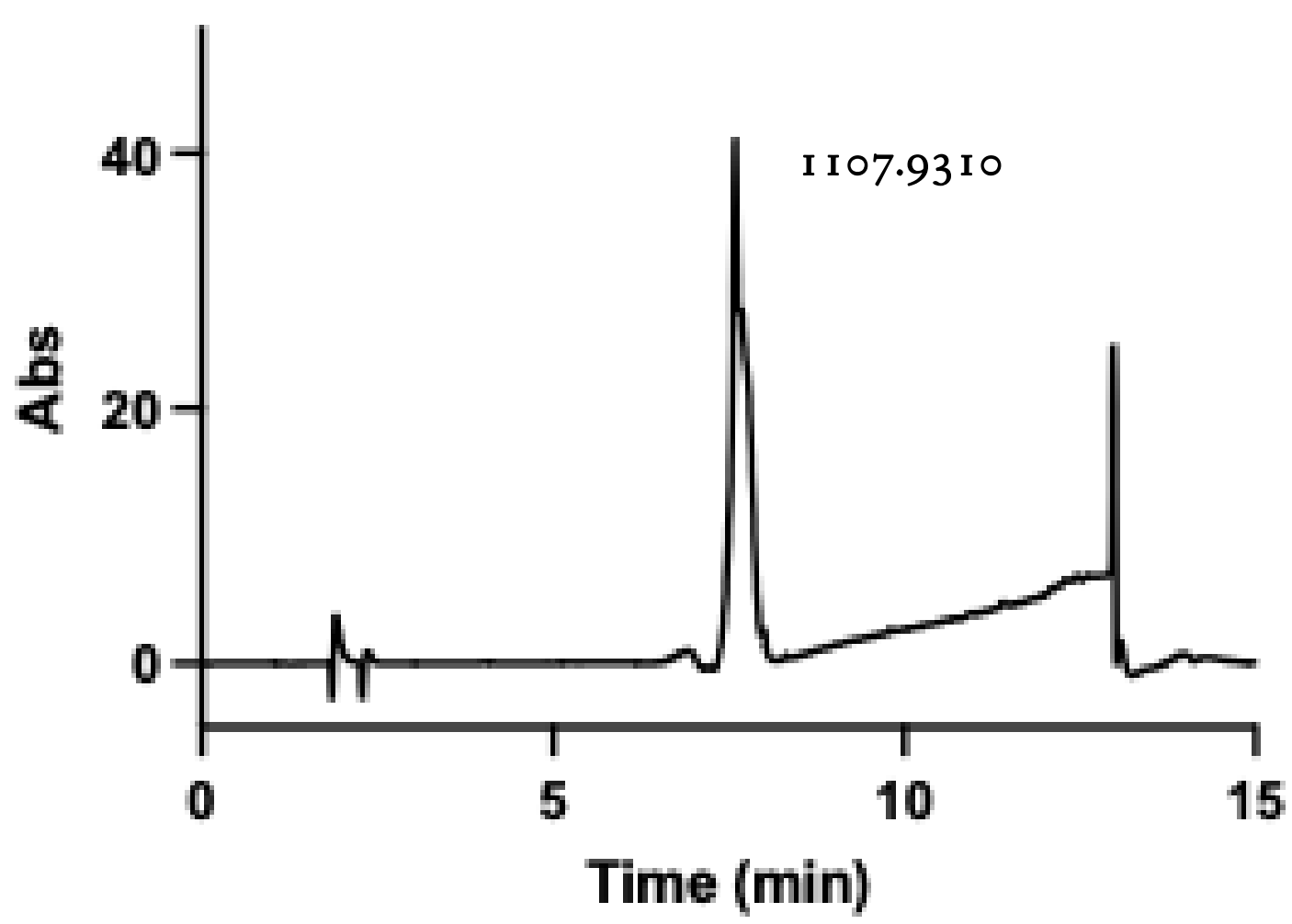

Chemical formula: $C_{90}H_{129}FN_{28}O_{29}S_4{}^{2+}$

Calculated $[M + 2H]^{2+}$ = 1107.41

Observed $[M + 2H]^{2+}$ = 1107.9310

Compound 33

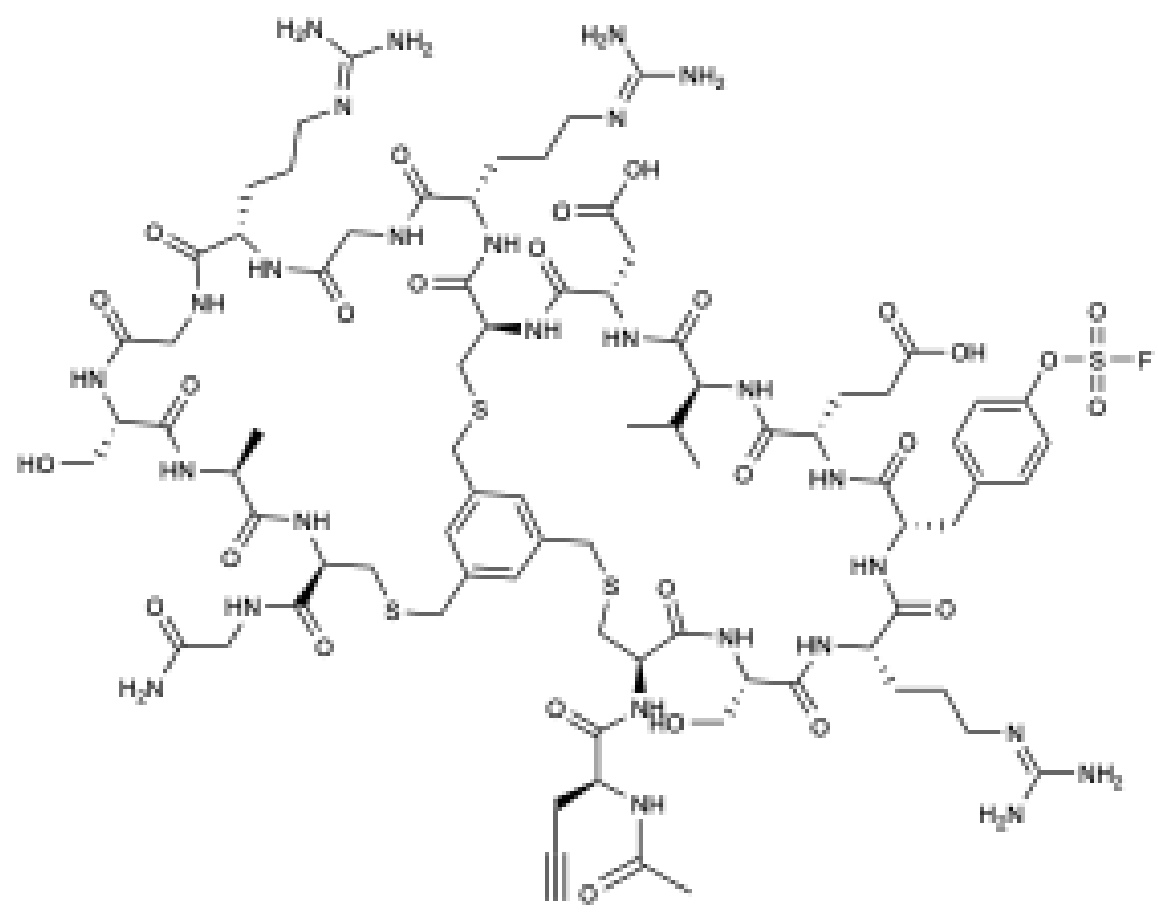

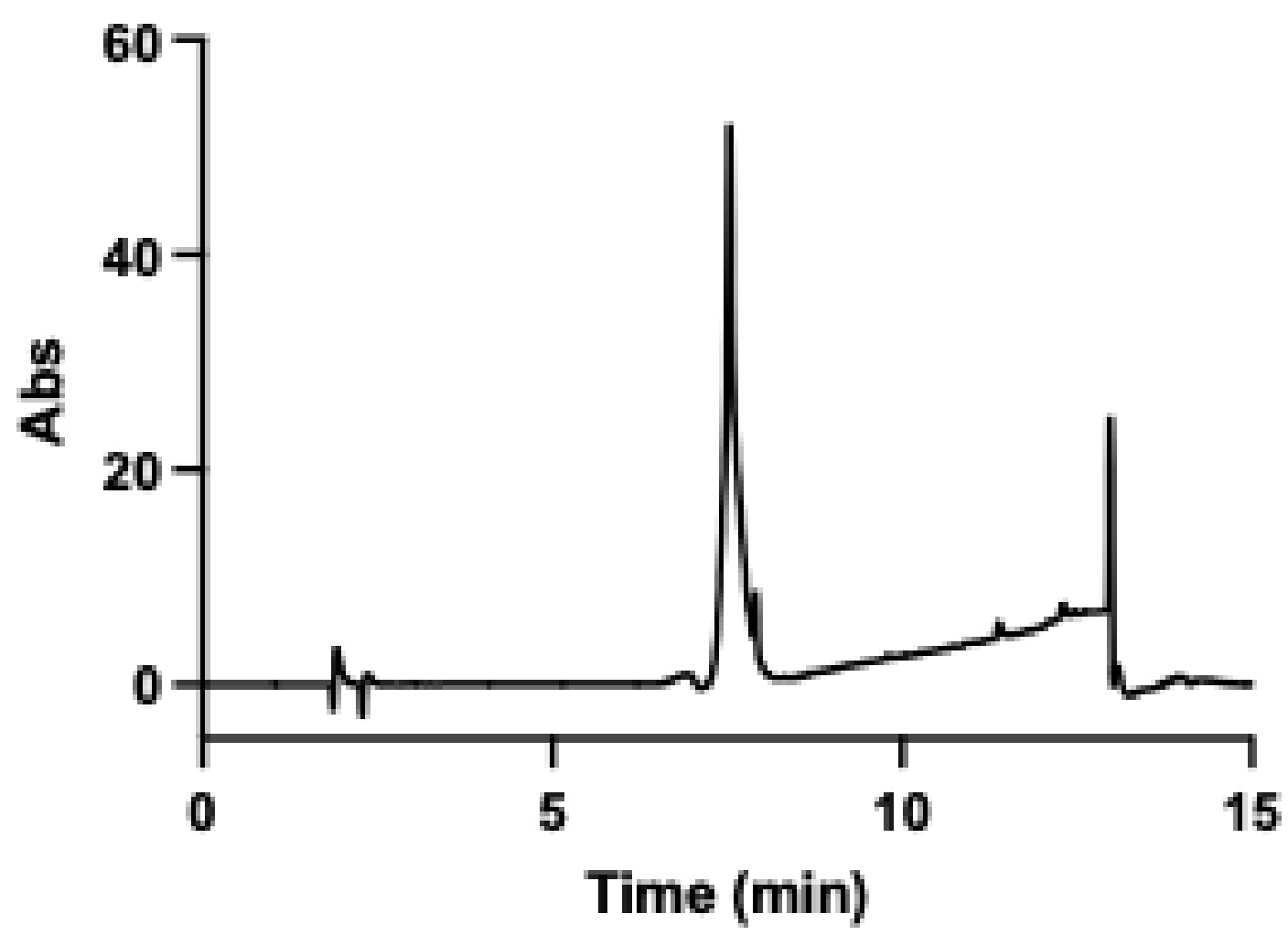

$C_{81}H_{120}FN_{27}O_{27}S_4$

Calculated $[M + 2H]^{+2}$ 1026.39

Observed $[M + 2H]^{2+}$ = 1026.3946

Compound 34

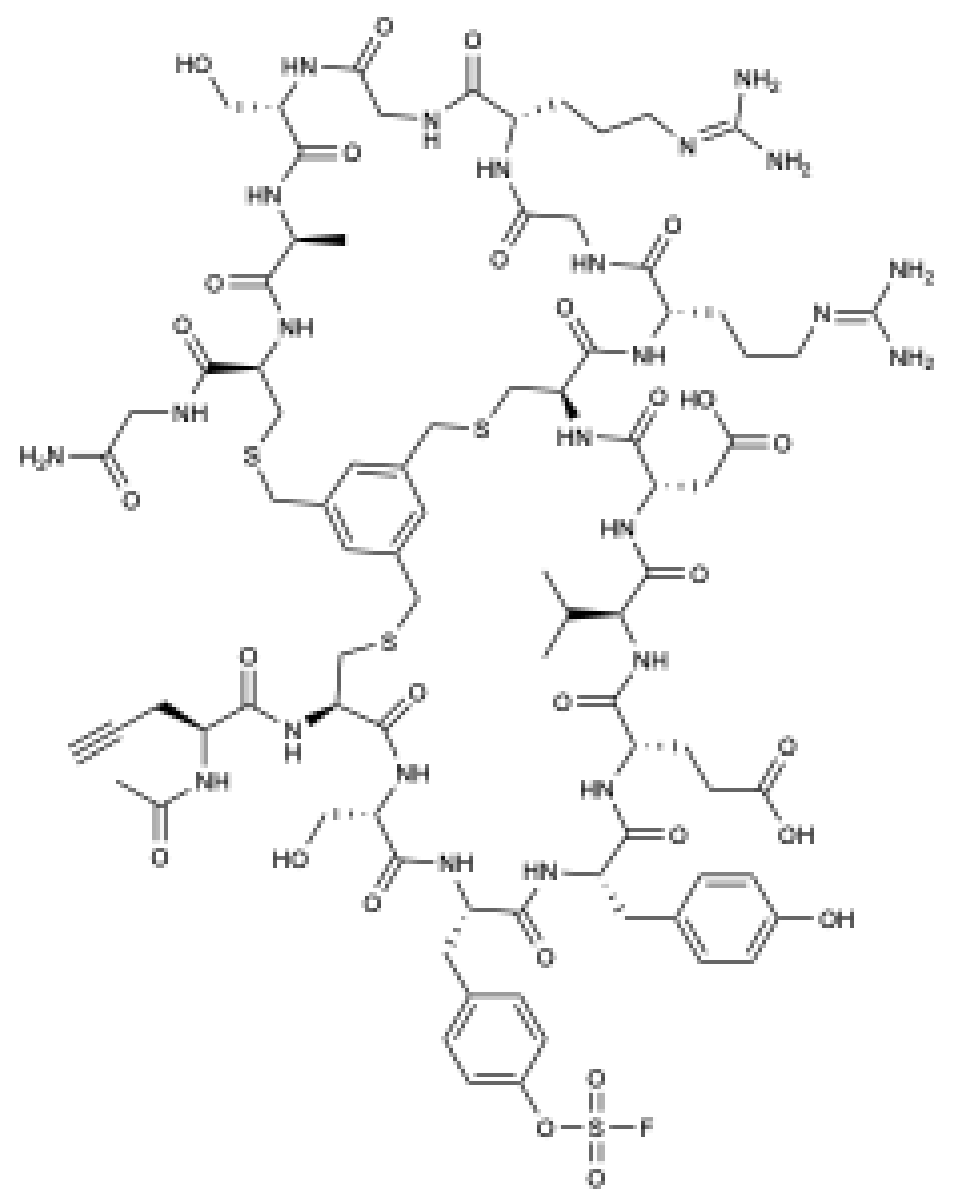

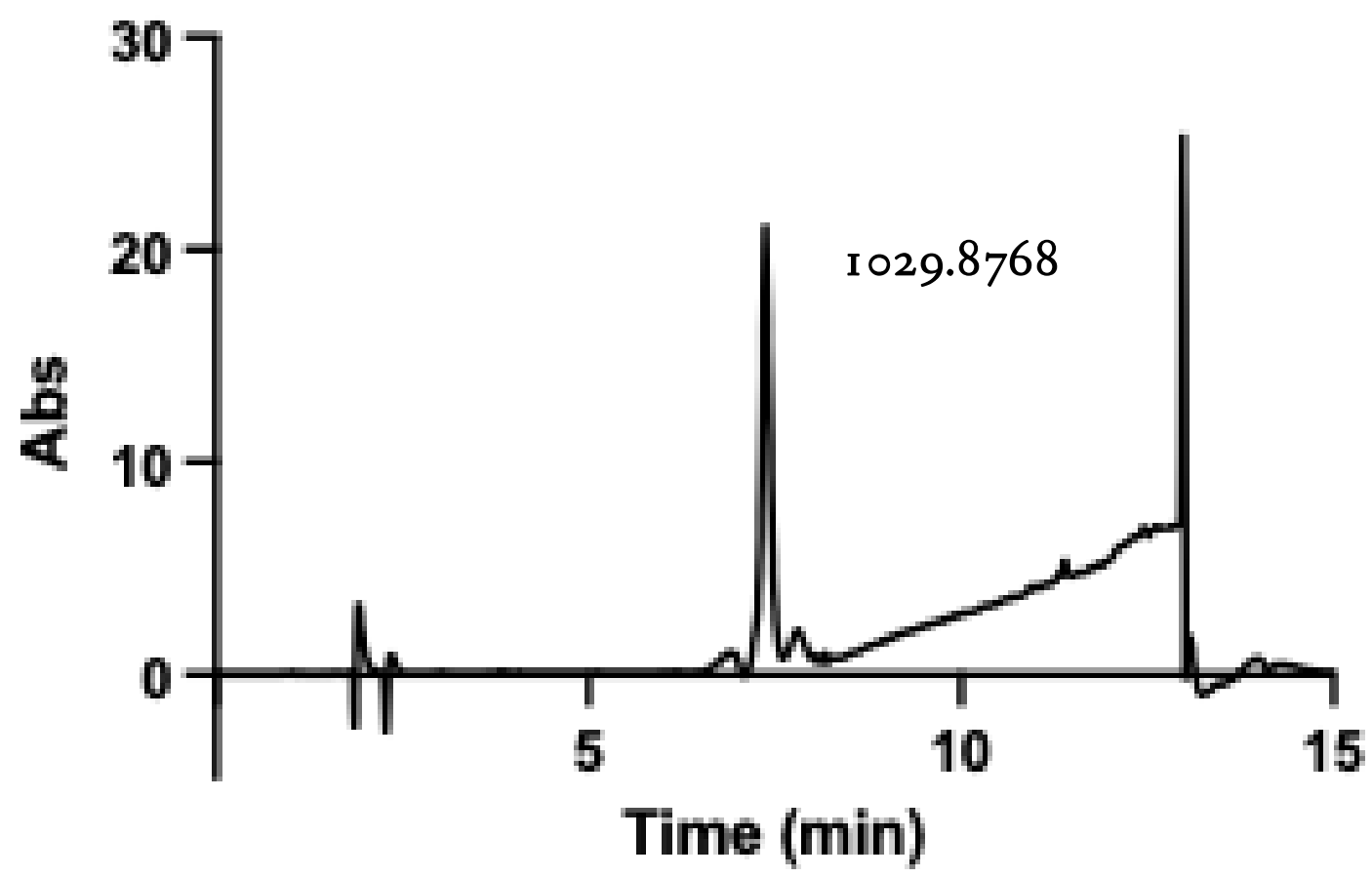

Chemical formula: $C_{84}H_{117}FN_{24}O_{28}S_4^{2+}$

Calculated $[M + 2H]^{2+}$ = 1029.36

Observed $[M + 2H]^{2+}$ = 1029.8768

6 References

1. S. Chen, S. Lovell, S. Lee, M. Fellner, P. D. Mace and M. Bogyo, Identification of Highly Selective Covalent Inhibitors by Phage Display, *Nat. Biotechnol.*, 2021, *39*, 490–498
2. V. Baeriswyl, H. Rapley, L. Pollaro, C. Stace, D. Teufel, E. Walker, S. Chen, G. Winter, J. Tite and C. Heinis, Bicyclic Peptides with Optimized Ring Size Inhibit Human Plasma Kallikrein and Its Orthologues while Sparing Paralogous Proteases, *ChemMedChem*, 2012, *7*, 1173–1176.
3. J. Tang, C. L. Yu, S. R. Williams, E. Springman, D. Jeffery, P. A. Sprengeler, A. Estevez, J. Sampang, W. Shrader, J. Spencer, W. Young, M. McGrath and B. A. Katz, Expression, Crystallization, and Three-dimensional Structure of the Catalytic Domain of Human Plasma Kallikrein, *J. Biol. Chem.*, 2005, 280, 41077–41089
